# Reactions of *N*,*O*- and *N*,*S*‑Azoles and -Azolines with Ozone: Kinetics and Mechanisms

**DOI:** 10.1021/acs.est.5c01323

**Published:** 2025-06-27

**Authors:** Simon A. Rath, Valentin Rougé, Julie Tolu, Daniel Rentsch, Maria Lia Halder, Urs von Gunten

**Affiliations:** † Eawag, Swiss Federal Institute of Aquatic Science and Technology, Dübendorf CH-8600, Switzerland; ‡ School of Architecture, Civil and Environmental Engineering (ENAC), Ecole Polytechnique Fédérale Lausanne (EPFL), Lausanne CH-1015, Switzerland; § 28501 Empa, Swiss Federal Laboratories for Materials Science and Technology, Dübendorf CH-8600, Switzerland

**Keywords:** ozonation, aromatic and nonaromatic *N*-heterocycles, thiazole, oxazole, oxazoline, thiazoline

## Abstract

*N*,*O*- and *N*,*S*-azoles and -azolines are common functional groups in pharmaceuticals, agrochemicals and natural products. Their fate during ozone-based water treatment processes is unknown due to a lack of kinetic and mechanistic information on their reactions with ozone. Apparent second-order rate constants *k*_O_3__ of 12 model compounds were determined at pH 7: oxazoles react 2 orders of magnitude faster (*k*_O_3__ = 9 × 10^2^–5 × 10^4^ M^–1^s^–1^, depending on their substituents) than thiazoles (*k*_O_3__ = 1 × 10^1^–2 × 10^3^ M^–1^s^–1^). The low *k*_O_3__ of thiazoles limits their degradability during ozonation. Only small yields of reactive oxygen species (^•^OH, H_2_O_2_, and ^1^O_2_) were observed during ozonation of oxazoles, suggesting that all oxygen atoms from ozone are incorporated into the products. Oxazoles and thiazoles react initially by a Criegee-type reaction at the C=C double bond, followed by two reaction branches, leading to two observed product groups: (1) carboxylates and cyanate; (2) formate, amide and CO_2_. For thiazoles, thiocarboxylic acids were identified as intermediates, reacting further to sulfate and carboxylic acids, forming ^1^O_2_. The nonaromatic 2-methyloxazoline is unreactive toward ozone. 2-Methylthiazoline reacts fast (*k*_O_3__ = 2 × 10^4^ M^–1^s^–1^), forming ^1^O_2_, leading to ring-opening and formation of dimerization products which react further to *N*-acetyltaurine. These results enhance the understanding of the ozone reactivity of heterocycles and help predict transformation product formation.

## Graphical abstract

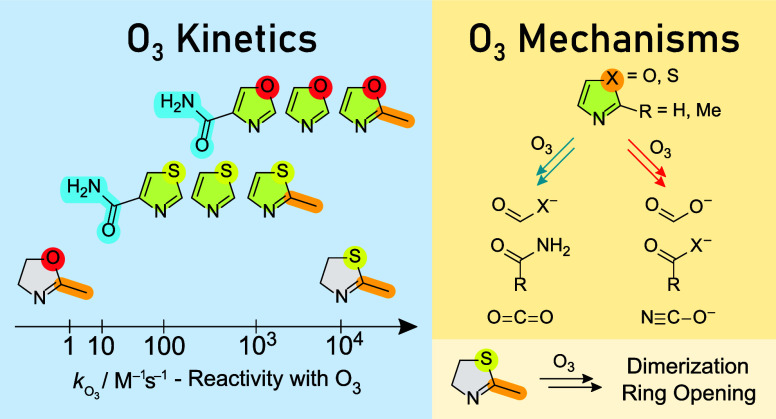

## 1 Introduction

Ozonation is applied in drinking water, enhanced wastewater treatment, and water reuse for disinfection and micropollutant abatement.^1–6^ During this process, ozone (O_3_) and hydroxyl radicals (^•^OH) react with micropollutants, leading to their abatement. Rather than complete mineralization, ensuing transformation products are formed which are controlled by reaction kinetics and mechanisms.^2,3,7^ Typically, ozonation reduces the intended bioactivities of micropollutants.^8–13^ However, in some cases toxicity increased after ozonation of wastewaters, due to reactions of ozone and ^•^OH with matrix components such as dissolved organic matter resulting in the formation of aldehydes and ketones, which are degraded again during biological post-treatment, reducing toxicity.^14–18^ To predict the extent of micropollutant abatement and transformation product formation, detailed knowledge of the reaction kinetics and mechanisms is crucial.^19–22^

Previous studies elucidated reaction kinetics and mechanisms for many classes of organic compounds.^2,20,23^ For instance, tertiary amines react with ozone with second-order rate constants (*k*_O_3__) ranging from 10^3^ to 10^8^ M^–1^s^–1^ for the neutral form, to form *N*-oxides.^10,24,25^ Primary and secondary amines have slightly lower *k*_O_3__ and mainly form nitro-compounds.^24,26^ Similar to tertiary amines, organic sulfides have *k*_O_3__ in the order of about 10^6^ M^–1^s^–1^ and are transformed into sulfoxides by oxygen atom transfer.^27–29^

However, the ozone reactivity of aromatic and nonaromatic heterocycles, particularly nitrogen-containing heterocycles, is not yet sufficiently understood. Previous studies have shown that heterocyclic compounds exhibit a wide range of reactivities toward ozone despite their structural similarities.^23,30,31^ Cyclic saturated amines have very similar chemical properties as noncyclic amines and react with ozone by a variety of pathways, including electron-transfer, oxygen transfer or H-abstraction.^30^ A very different kind of reactivity is expected for unsaturated nitrogen-containing heterocycles due to their predominantly aromatic nature. For six-membered aromatic heterocycles like pyridine (Scheme S1.1, Supporting Information), the reactivity with ozone is very low, despite the free electron-pair at the nitrogen (*k*_O_3_+pyridine_ = 1.6–3.2 M^–1^s^–1^).^31–33^ In contrast, five-membered aromatic *N*-heterocycles, where the lone-pair of the nitrogen is part of the aromatic electron system exhibit a very different reactivity: pyrrole and imidazole exhibit a high reactivity (*k*_O_3_+pyrrole_ = 1.4 × 10^6^ M^–1^s^–1^; *k*_O_3_+imidazole_ = 2.3 × 10^5^ M^–1^s^–1^) while pyrazole is much less reactive (*k*_O_3_+pyrazole_ = 56 M^–1^s^–1^) despite having the same initiating reaction: an ozone attack at the C=C double bond (Scheme S1.1).^34^ Pyrrole reacts either through the Crigee mechanism,^2^ leading to ring opening products, or forms derivatives of maleimide. Imidazole primarily undergoes a Criegee-type reaction, forming low-molecular weight compounds like formate, cyanate and formamide.^34^ The reaction mechanism of the least reactive species pyrazole is less understood, but likely involves either hydroxylation of the C=C double bond or a Criegee-type reaction.^34^

These five-membered nitrogen-containing heterocycles are an important class of functional groups because of their widespread use in medicinal and agricultural fungicides (for example thiabendazole, etoxazole, pimprinine), insecticides (for example thiamethoxam, chothianidin) and other pharmaceuticals (for example thiangazole, oxaprozin).^35^ Additionally, they are present in various natural products such as aerucyclamides, which are secondary metabolites of cyanobacteria containing *N*,*O*- and *N*,*S*-azoles, potentially produced during cyanobacterial blooms.^36^

Currently, there is only limited data available for *N*,*O*- and *N*,*S*-azole reactions with ozone (for chemical structures see Table 1). The second-order rate constants for three isoxazole­(1,2-oxazole)-containing antibacterial compounds were determined previously (Scheme S1.1).^37^ Two of these, where the isoxazole is likely the primary reactive site, exhibit low reactivities, similar to pyrazole (3,5-dimethylisoxazole: *k*_O_3__ = 54 M^–1^s^–1^ acetylsulfamethoxazole: *k*_O_3__ = 260 M^–1^s^–1^).^37^ Benzothiazole (a 1,3-thiazole, Scheme S1.1) also shows low reactivity (*k*_O_3__ = 2.3 M^–1^s^–1^).^38^ For the aerucyclamides containing 1,3-oxazole (aerucyclamide C, microcyclamide 7806A/B) a moderate reactivity was observed (*k*_O_3__ ≈ 5 × 10^3^ M^–1^s^–1^), while aerucyclamides A and B (containing 1,3-thiazoles, -oxazolines and -thiazolines) again show much lower reactivities (*k*_O_3__ < 10^2^ M^–1^s^–1^) (Scheme S1.1).^36^ Other azole-containing chemicals have not yet been studied, highlighting the need for further investigations to assess their fate during ozonation.

**Table 1:**
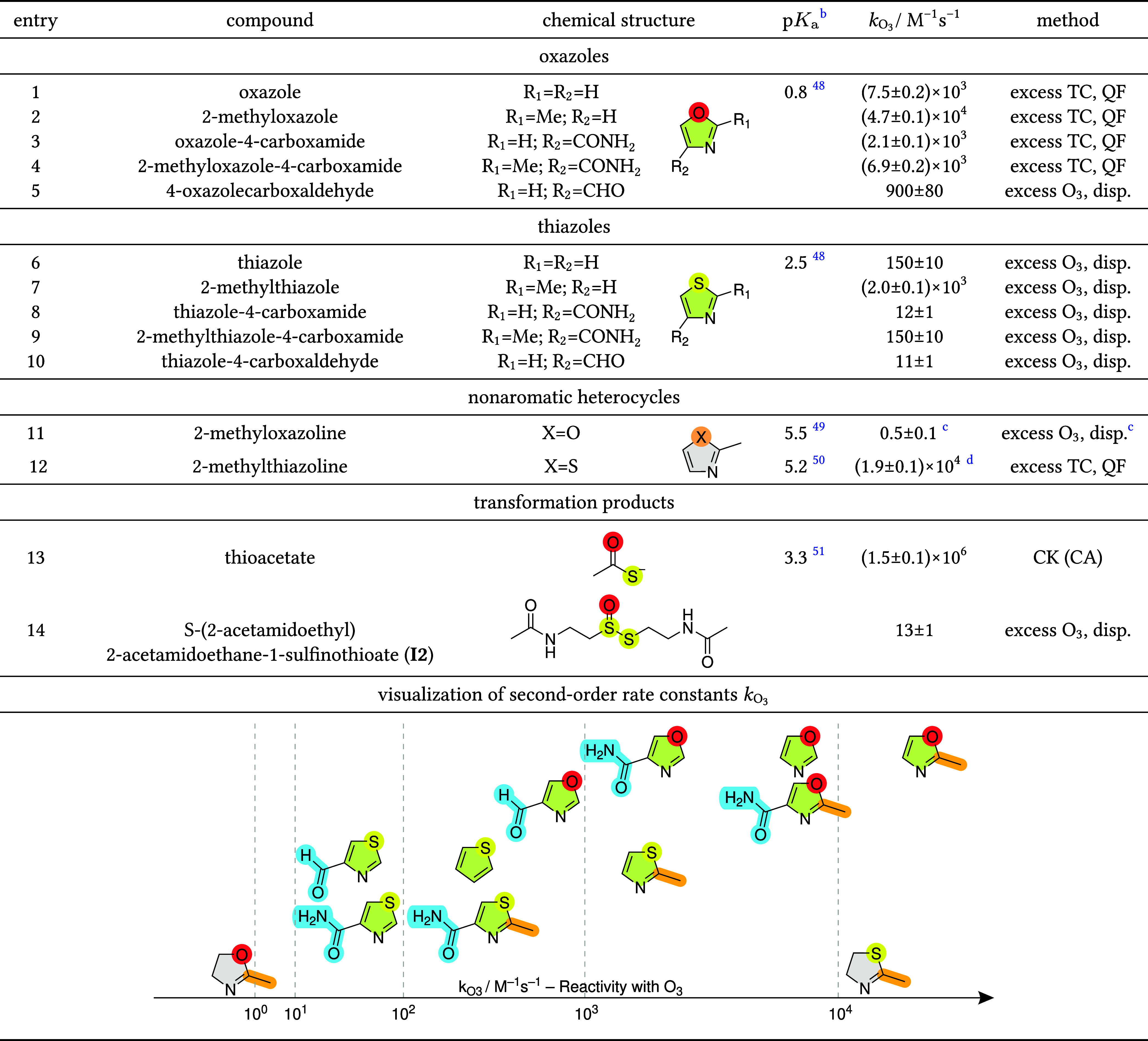
Apparent Second-Order Rate Constants at pH 7 for the Reactions of Ozone with the Selected Model Compounds and Selected Transformation Products (See Sections 3.2.2 and 3.2.4)­^a^

a The error margins of *k*_O_3__ represent the standard deviation. Reaction kinetics were measured in phosphate buffered solution (10 mM, pH 7) in the presence of *tert*-butanol (10 mM) as an ^•^OH scavenger, by (i) excess target compound (TC) in a quench-flow (QF) reactor by monitoring of the ozone decrease with the indigo method (duplicates), (ii) in excess of ozone and monitoring both the ozone and target compound decrease with the cinnamic acid method (measurement of target compound and benzaldehyde by LC) by taking samples by a dispenser (triplicates), or (iii) competition kinetics (CK) with cinnamic acid as competitor (duplicates). Color code: oxygen: red; sulfur: yellow; aromatic ring: green; non-aromatic ring: grey; electron-donating substituent: orange; electron-withdrawing substituent: cyan.

b p*K*_a_ of corresponding acid MH^+^, if available.

c Ozone decrease was measured spectrophotometrically as described in Section S3.1.

d This second-order rate constant might be slightly overestimated (< factor 2), due to the reaction of ozone with the initial transformation product (**I1**, see Section 3.2.4). A 10-fold excess of substrate should limit this effect, but it cannot be excluded (see Section 3.2.4). Moreover, a faster reacting impurity in the substrate partly consumed ozone at the beginning of the reaction.

The objectives of this study were to determine kinetics and mechanisms of reactions between ozone and *N*,*O*- and *N*,*S*-azoles and the corresponding nonaromatic azolines. Oxazole, thiazole, and substituted analogues as well as 2-methyloxazoline and 2-methylthiazoline were selected as model compounds (for structures see Table 1). Apparent second-order rate constants for their reactions with ozone were determined at pH 7 and the formation of reactive oxygen species (ROS) and transformation products were investigated. Identification and quantification of transformation products involved different analytical techniques, including ion chromatography (IC) with conductivity detection (CD), liquid chromatography (LC) and IC coupled to high-resolution tandem mass spectrometry (HRMS/MS) and inductively coupled plasma tandem mass spectrometry (ICP-MS/MS) as well as nuclear magnetic resonance (NMR). Based on these findings, reaction mechanisms for the reaction of ozone with target compounds were proposed.

## 2 Materials and Methods

All chemicals used in this study, including purities and suppliers, and the generation of ozone stock solution are provided in Section S2. All experiments were performed at pH 7.0 ± 0.1 in 5 or 10 mM phosphate buffer (23 ± 1 °C) with 10 mM *tert*-butanol to quench ^•^OH.

Apparent second-order rate constants for ozone reactions with the target compounds were determined using three methods under pseudo first-order conditions, depending on the magnitude of the second-order rate constant and the stoichiometry of the reaction (see Table 1): (1) a dispenser setup with excess ozone and monitoring of target compound abatement (*k*_O_3__ < 10^3^ M^–1^s^–1^),^39^ (2) a quench-flow setup with excess of target compound and monitoring the ozone decrease (*k*_O_3__ = 10^3^ to 10^5^ M^–1^s^–1^),^40^ and (3) competition kinetics experiments with cinnamic acid (competitor) (*k*_O_3__ > 10^5^ M^–1^s^–1^).^37^ Details are provided in Section S3.

Ozone dosing experiments were performed by adding the required volumes of an ozone stock solution (0.01–0.5 mM) to azole, azoline or thioacetate solutions (20 mL, approximately 0.1 mM). To determine the dosed ozone accurately, for every ozone dose, a separate 20 mL reaction vessel with cinnamic acid was ozonated (unless stated otherwise), as described previously.^41^ All experiments including additional experiments with thiazole and 2-methylthiazoline are described in detail in Section S4.

Details of the analytical methods are provided in Section S5. Azoles, azolines and benzaldehyde were quantified by LC-UV. Ionic transformation products (acetate, formate, sulfate, etc.) were quantified by IC-CD. Acetamide was quantified by LC-HRMS/MS. *m*/*z* of one transformation product was obtained by IC-HRMS/MS. The organic reaction products of 2-methylthiazoline were quantified by LC-ICP-MS/MS,^42–44^ and identified by LC-HRMS/MS and NMR. Singlet oxygen (^1^O_2_) was quantified by detecting the phosphorescence emitted by ^1^O_2_ at 1270 nm by a near-infrared photomultiplier tube, as described previously.^24^ ^•^OH were quantified by *tert*-butanol: formaldehyde, the reaction product with a reported yield of ∼50%, was quantified by derivatization with 2,4-dinitrophenylhydrazine and quantification of 2,4-dinitrophenylhydrazone via LC-UV, as described previously.^45^ Hydrogen peroxide (H_2_O_2_) was quantified by ^1^O_2_ measurement (see above) produced from the reaction of HO_2_^–^ with HOCl.^29,46,47,48,49,50,51^

## 3 Results and Discussion

### 3.1 Ozone Reaction Kinetics

Apparent second-order rate constants *k*_O_3__ at pH 7 were determined for five oxazole derivatives, five thiazole derivatives and for 2-methyloxazoline and 2-methylthiazoline (Table 1, entries 1–12). Due to the low p*K*_a_ values of the azoles and azolines (0.8–5.5) the reactions of the generally faster-reacting neutral species are dominant at pH 7.^2^ Additionally, *k*_O_3__ for two detected transformation products, thioacetate and *S*-(2-acet-amido-ethyl)­2-acetamidoethane-1-sulfinothioate (**I2**) were determined (Table 1, entries 13–14). For thioacetate, the dominant reactive species is the deprotonated, negatively charged.^2^

Unsubstituted oxazole reacts about 1.5 orders of magnitude faster with ozone than thiazole (Table 1, entries 1 and 6), though both are significantly less reactive than the *N*,*N*-analogue imidazole (*k*_O_3_+imidazole_ = 2.3 × 10^5^ M^–1^s^–1^).^34^ Despite thiazoles’ higher electron density, its greater aromaticity may decrease its reactivity toward ozone due to the stabilization of the π-electron system.^52–55^ A similar trend was observed for the reactions of ^1^O_2_ with oxazoles and thiazoles with *k*_^1^O_2_+oxazole_ 2 orders of magnitude higher than *k*_^1^O_2_+thiazole_.^56^ In contrast, although imidazole is more aromatic than oxazole,^53–55^ in this case the lower electronegativity of nitrogen compared to oxygen in oxazole increases electron density at the C=C double bond in imidazole, leading to its higher ozone reactivity.^57^

Oxazoles are less reactive than furans, which have second-order rate constants for the reactions with ozone in the range of 10^5^ to 10^6^ M^–1^s^–1^.^58,59^ This is likely because furans contain two conjugated C=C double bonds and only one heteroatom in the ring, resulting in higher electron density in the C=C double bonds.^57^ The same trends were observed for pyrrole and imidazole (*k*_O_3_+pyrrole_ = 1.4 × 10^6^ M^–1^s^–1^; *k*_O_3_+imidazole_ = 2.3 × 10^5^ M^–1^s^–1^).^34^

A distinct substituent effect is evident for oxazoles and thiazoles: a methyl-group at the 2-position enhances the reactivity by approximately 1 order of magnitude due to elevated electron density (Table 1, entries 2 and 7). Conversely, electron-withdrawing groups (carboxaldehydes or carboxamides at the 4-position, Table 1, entries 3, 5, 8 and 10) reduce reactivity by 1 order of magnitude. When both types of substituents are present, their opposing effects neutralize each other resulting in reactivities, similar to the unsubstituted oxazole and thiazole (Table 1, entries 4 and 9). This highlights the importance of substitution for the ozone reactivity of aromatic heterocycles and is in line with the reactivities of phenols, olefins and furans.^57,58,60^

The ozone-reactivities of the nonaromatic heterocycles 2-methyloxazoline and 2-methylthiazoline are very different (Table 1, entries 11–12). While 2-methylthiazoline reacts readily with ozone (*k*_O_3_+2‑methylthiazoline_ = 1.9 × 10^4^ M^–1^s^–1^), 2-methyloxazoline has very low ozone reactivity (*k*_O_3_+2‑methyloxazoline_ = 0.5 M^–1^s^–1^). The elevated *k*_O_3__ for 2-methylthiazoline compared to its aromatic analogue, 2-methylthiazole (*k*_O_3_+2‑methylthiazole_ = 2.0 × 10^3^ M^–1^s^–1^), may be explained by the electronic configuration of the sulfur: in thiazole, the sulfur is part of the aromatic electron system, in thiazoline, this system does not exist. Nevertheless, 2-methylthiazoline is much less reactive than thioethers, with apparent second-order rate constants in the range of 10^5^ to 10^6^ M^–1^s^–1^.^27,32,61^ This may be due to conjugation with the electron-withdrawing nitrogen reducing the electron density at the sulfur. A similar deactivation is reported for the thiocarbamate pesticide molinate (Scheme S1.1), containing a thioether with a neighboring amide (*k*_O_3_+molinate_ ≈ 5 × 10^2^ M^–1^s^–1^).^62^

For the determined *k*_O_3__ of aerucyclamides (Scheme S1.1), as expected the oxazole-containing aurucyclamide C and microcyclamides 7806A/B exhibit the highest reactivities (*k*_O_3__ ≈ 5 × 10^3^ M^–1^s^–1^).^36^ Contrary to the results for 2-methylthiazoline, the thiazoline-containing aerucyclamide A exhibits a far lower reactivity (*k*_O_3__ ≈ 88 M^–1^s^–1^).^36^ An explanation for this discrepancy is currently missing.

### 3.2 Transformation Products and Reaction Pathways for the Reactions of Azoles and Azolines with Ozone

To determine ozone reaction mechanisms, yields of ROS (^1^O_2_, H_2_O_2_ and ^•^OH) and transformation products were determined.^2,24,29,34,45,63^ The corresponding results are summarized in Table 2.

**Table 2:** Reaction Stoichiometries, and Yields of Reactive Oxygen Species (ROS) and Transformation Products for the Reactions of Oxazole, 2-Methyloxazole, Thiazole, 2-Methylthiazole and 2-Methylthiazoline with Ozone^a^

compound	[ozone]:[azole] stoichiometry^b^	ROS and transformation product yields in %^c^								
		^•^OH	H_2_O_2_	^1^O_2_	formate	cyanate	acetate	sulfate	acetamide	thioacetate
oxazole	1.1	<1	5	1	194	112				
2-methyloxazole	1.0	<1	7	1	112	6	8		89	
thiazole	3.7	<1	1	30	154	94		88		
2-methylthiazole	2.6	<1	2	29	105	55	39	59	26	11
2-methylthiazoline^d^	0.5–1.0^e^	<1	1	88^f^	^d^					

a The experiments were performed at pH 7 (Phosphate Buffer, 5 mM) in the Presence of *tert*-Butanol (10 mM)

b The reaction stoichiometry was determined from the inverted slopes of the linear regression of the substrate concentration after ozonation with molar [ozone]:[azole] ratios <0.9. The linear regressions are shown in Figures 1, 2 and 3a as dashed lines.

c The yields of the ROS are expressed relative to the consumed ozone (mol/mol %) and the yields of the transformation products are expressed relative to the azole consumption (mol/mol %). The yields of the transformation products were calculated from the slopes of their formation divided by the slopes of abatement of the parent compounds with molar [ozone]:[azole] ratios <0.9 (Figures 1 and 2). The determined error values obtained from duplicate experiments are smaller than 5% of the determined yields and are not shown for clarity.

d The organic products and the mechanism of the oxidation of 2-methylthiazoline are discussed separately in Section 3.2.4.

e The stoichiometry of this reaction depends on the reaction conditions and is discussed in Section 3.2.4.

f This value was obtained at an [azoline]_0_:[O_3_]_0_ = 1.1. At other ratios, different values were obtained (Section S10.1).

#### 3.2.1 Reaction of Oxazole or Thiazole with Ozone

##### 3.2.1.1 ROS

The reaction of ozone with oxazole only yields trace amounts of ^1^O_2_ and ^•^OH, along with 5% H_2_O_2_ (Table 2). This implies that nearly all oxygen atoms of ozone are integrated into the transformation products (see Section 3.2.3). The formation of H_2_O_2_ could arise from Criegee-type reactions at the C=C double bond,^64^ however the expected carbonyl products were not detected. The reaction of ozone with thiazole similarly resulted in only minor yields of ^•^OH and H_2_O_2_. In contrast to the reaction of oxazole, about 30% of ^1^O_2_ was produced based on consumed ozone (Table 2). The molar reaction stoichiometry [ozone]:[thiazole] of 3.7 (Figure 1b, blue dotted line), indicating that approximately one equivalent of ^1^O_2_ is produced per ∼4 equivalents of ozone.

**Figure 1:**
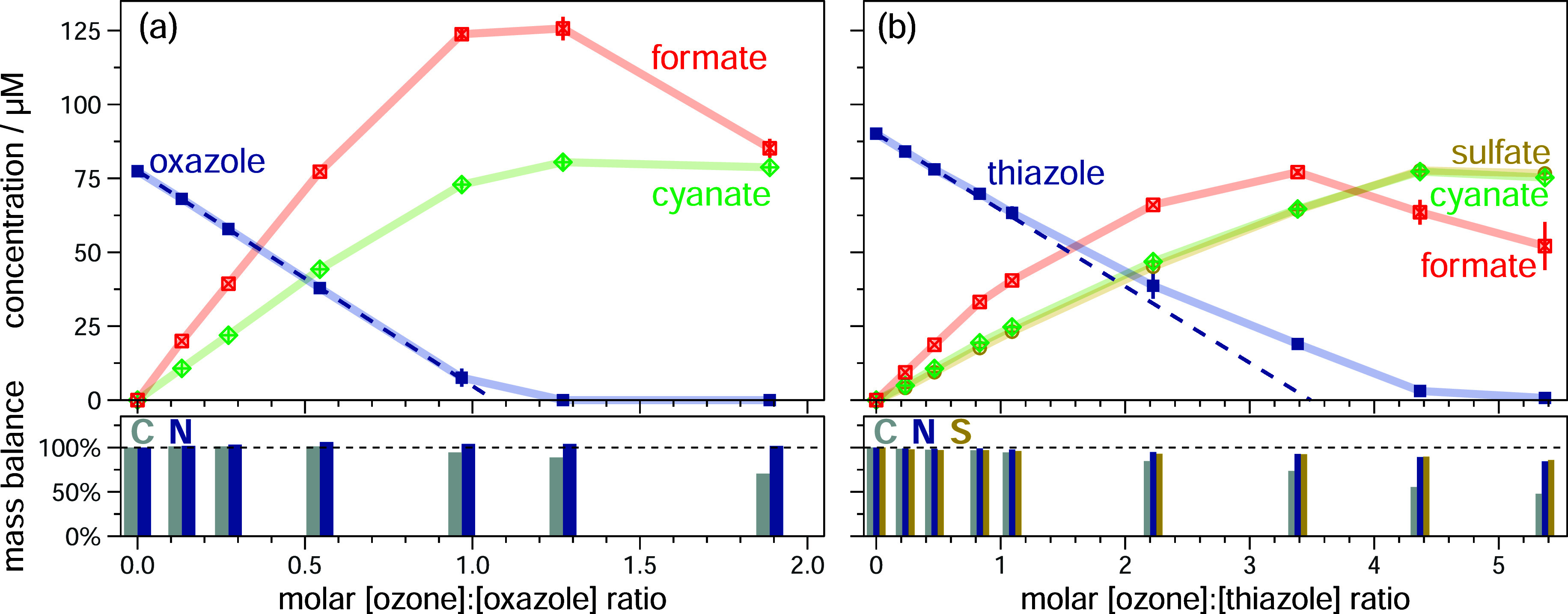
Abatement of (a) oxazole and (b) thiazole and formation of detected transformation products as a function of the molar [ozone]:[azole] ratio. Results are shown as averages from duplicate experiments, error bars represent the upper and lower value (if not visible, the range falls within the symbol size). The C, N and S mass balances for the transformation of (a) oxazole and (b) thiazole are shown at the bottom, which are obtained by the sum of the compound concentrations multiplied by the number of respective atoms in the compound at every ozone dose relative to the initial concentration of azole. The experiments were performed in the presence of the ^•^OH scavenger *tert*-butanol. Experimental conditions: [azole] = ∼0.1 mM; ozone doses = 0–0.5 mM; [*tert*-butanol] = 10 mM; [phosphate buffer (pH 7)] = 5 mM.

##### 3.2.1.2 Oxidation Products

The abatement of oxazole and thiazole and the formation of transformation products as a function of the molar [ozone]:[azole] ratios are shown in Figure 1. In contrast to thiazole (see above), oxazole reacts with a stoichiometry of 0.9 with ozone (Figure 1a, blue dotted line). Both reactions yield formate (oxazole: 194%, thiazole: 154%) and cyanate (oxazole: 112%, thiazole: 94%), and in the case of thiazole, 88% sulfate is formed additionally. All product formations are directly correlated to substrate decrease, indicating either a direct formation from the substrate or a very fast secondary reaction with ozone.

Formate (*k*_O_3_+formate_ = 46 M^–1^s^–1^)^65,66^ competes with the abatement of azoles by ozone, especially in the case of thiazole (*k*_O_3_+thiazole_ = 148 M^–1^s^–1^, Table 1), as its reactivity is only about a factor of 3 lower. This competition explains the deviation from linearity with increasing azole abatement (Figure 1b). The higher demand of ozone in the case of thiazole indicates that sulfate is formed via fast-reacting intermediates. To isolate the initial ozone attack on thiazole, an experiment with up to 100-fold excess of thiazole was performed (details in Section S6). Even under these conditions, the sulfate yield remained almost constant (90% of the original), with only a modest increase in formate and cyanate yields (121% and 122%, Figure S6.1). This indicates that subsequent sulfate formation from intermediates is much faster than the initial ozone attack on thiazole. Due to the sulfur atom in thiazole, thioformate could be a possible intermediate, in analogy to formate for oxazole (see also Sections 3.2.2 and S8 for 2-methylthiazole). However, no efforts were made to quantify it due to the almost complete mass balance.

The mass balances for carbon, nitrogen and sulfur for oxazole and thiazole remain nearly complete until significant amounts of formate are consumed by ozone (formation trend with a maximum and decrease at elevated ozone doses, Figure 1, red squares). Ozonation of formate results in CO_2_,^2,66^ which was not monitored. Given the completeness of the mass balance, the formation of formamide, another potential product (see Section 3.2.3), was not investigated, since it is a very minor product, if formed at all. Further oxidation of the nitrogen to nitrate was only observed in traces (<1%).

All identified products of the unsubstituted azoles are analogues to those observed during imidazole ozonation.^34^ Because of their differing chemical compositions, the ozonation of imidazole results in the formation of formamide, whereas oxazole produces formate. Thiazole requires two to three more equivalents of ozone to oxidize sulfur to sulfate and generates a second equivalent of formate.

##### 3.2.1.3 Ozonation of Oxazole-4-carboxamide

Additionally, the ozonation of oxazole-4-carboxamide has been studied (Section S7). Compared to unsubstituted oxazole, additional cyanate formation was observed (yields: formate: 151%; cyanate: 147%, Figure S7.1). Thus, a similar reaction can be assumed, but the additional amide group forms cyanate during ozonation. The missing ∼25% mass balance has not been identified (Figure S7.1, bottom). A mechanism, according to the discussion in Section 3.2.3 is proposed in Scheme S7.1.

#### 3.2.2 Reactions of 2-Methyloxazole and 2-Methylthiazole with Ozone

##### 3.2.2.1 ROS

Similar to oxazole and thiazole, the yields of ROS in the reaction of ozone with 2-methyloxazole are also low, while 2-methylthiazole forms ^1^O_2_ with a yield of 29% (Table 1). Analogously, this means that with 2-methyloxazole all oxygen atoms of the ozone are incorporated into the products and with 2-methylthiazole ^1^O_2_ is formed in one of the three reaction steps during the conversion of organic sulfur to sulfate.

##### 3.2.2.2 Oxidation Products

The abatement of 2-methyloxazole and 2-methylthiazole and the formation of transformation products as a function of the molar [ozone]:[azole] ratios are shown in Figure 2. Analogous to oxazole, 2-methyloxazole is fully abated with one equivalent of ozone. 2-Methylthiazole has an [ozone]:[azole] stoichiometry of 2.6, which can be attributed to the oxidation of sulfur-containing intermediates (see Section 3.2.1). Similar to unsubstituted derivatives, the transformation products formate, acetate, cyanate and sulfate were identified (yields: Table 2). Moreover, acetamide was detected in both cases, and thioformate (not quantified, Section S8 and Figure S8.1a) and thioacetate (Figure S8.1b and Table 2) were identified as intermediates of 2-methylthiazole, which are further oxidized at higher ozone doses (Figures 2b and S8.2).

**Figure 2:**
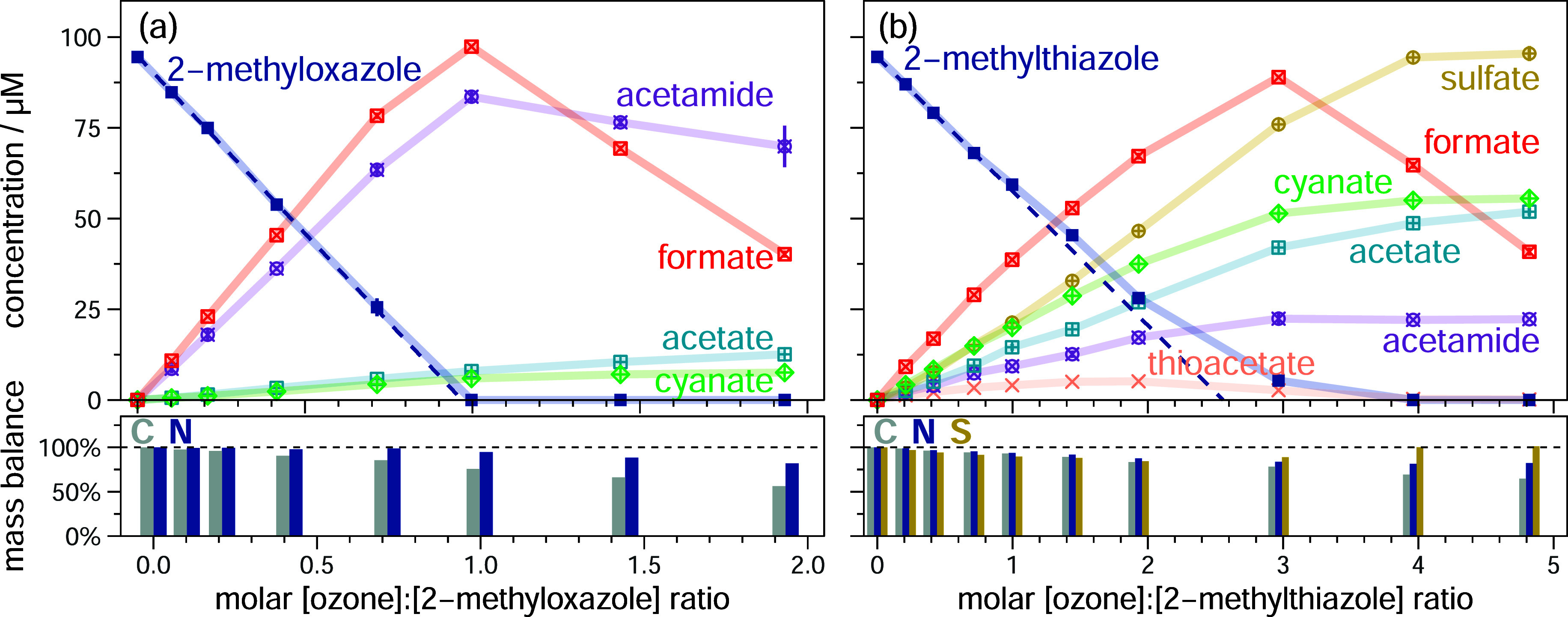
Abatement of (a) 2-methyloxazole and (b) 2-methylthiazole and formation of detected transformation products as a function of the molar [ozone]:[azole] ratio. Results are shown as average from duplicate experiments, error bars represent the upper and lower value (if not visible, the range falls within the symbol size). The C, N and S mass balances are shown at the bottom, which are obtained by the sum of the compound concentrations multiplied by the number of respective atoms in the compound at every ozone dose relative to the initial concentration of azole. The experiments were performed in the presence of the ^•^OH scavenger *tert*-butanol. Experimental conditions: [azole] = ∼0.1 mM; ozone doses = 0–0.5 mM; [*tert*-butanol] = 10 mM; [phosphate buffer (pH 7)] = 5 mM.

The formation of acetamide as a major product (2-methyloxazole: 89%; 2-methylthiazole: 26%; Table 2) was unexpected, as no equivalent product was observed for the unsubstituted analogues (see above). In contrast, the yields of the expected acetate (analogous to formate, due to the additional methyl group) and cyanate are quite low (2-methyloxazole: 8%, 6%; 2-methylthiazole: 39%, 55%, respectively; Table 2). The similar yields of acetate and cyanate suggest that their formation is interrelated, while acetamide seems to be the product of an alternative reaction pathway, with formate (approximately 100% yield) being formed in both pathways. The mechanistic implications are discussed in Section 3.2.3.

The lower [ozone]:[azole] abatement stoichiometry observed for 2-methylthiazole, compared to thiazole, can be attributed to its more than 10-fold higher reactivity (Table 1, entries 6 and 7). This reduces first the impact of ozone self-decay, and second the impact of secondary and tertiary reactions with sulfur-containing intermediates. One of these formed intermediates is thioacetate, which has been quantified (Figures 2b, S8.1b and S8.2; see Section S9 for the reaction of thioacetate with ozone). Because thioacetate is formed as an intermediate, reacting further to acetate and sulfate, acetate formation has initially a smaller slope, compared to cyanate, while at higher ozone doses, the same yields are reached (ca. 55%, Figure 2b). Similarly, the slope of sulfate formation at small ozone doses accounts only for a yield of 59%, but for higher ozone doses the sulfate yield increases, closing the sulfur mass balance (100% sulfate, Figure 2b, bottom, yellow bars). Additionally, the thioformate formation trend matches well with that of thioacetate (Figure S8.2). Furthermore, an intermediate that is oxidized at higher ozone doses was observed with IC-CD (Figure S8.3a,d). A *m*/*z* of 90.9861 was measured by IC-MS, corresponding to the sum formula [C_2_H_4_O_2_S] – [H^+^], equivalent to thioacetate + [O] (Figure S8.3b,c). This intermediate is also observed during ozonation of thioacetate (Section S9), and thus is likely an oxidation product thereof (for the discussion of its structure and formation, see Section S9). The formation of thioformate and this intermediate may partly explain the incomplete sulfur mass balance for molar [ozone]:[2-methylthiazole] ratios of 0.5–3 (Figure 2b, bottom).

##### 3.2.2.3 Reaction of Thioacetate with Ozone

The reaction of thioacetate is described in Section S9. In summary, it has a high reactivity with ozone (*k*_O_3_+thioacetate_ = 1.5 × 10^6^ M^–1^s^–1^, Table 1) and reacts with an ozone:thioacetate stoichiometry of 1.8, indicating a two-step reaction. The ^1^O_2_ yield at a molar [ozone]:[thioacetate] ratio of 1.9 was 71% relative to thioacetate and 38% relative to ozone, suggesting that one of the two oxidation steps yields ^1^O_2_ (Table S9.1 and Figure S9.1). The two products acetate and sulfate were quantified, reaching both about 100% yield at higher ozone doses (Figure S9.2), but sulfate is formed with a certain delay, indicating the presence of another sulfur-containing intermediate. Low concentrations of sulfite were detected with IC-CD but could not be quantified due to coelution with sulfate as a peak-shoulder (Figure S9.3). A second intermediate was observed with IC-CD and identified with IC-HRMS/MS as thioacetate + [O] (Figure S9.4) which is also observed in the oxidation of 2-methylthiazoline (see above and Figure S8.3). This intermediate is further oxidized at higher ozone doses (Figures S9.2 and S9.4d).

#### 3.2.3 Mechanism for the Reaction of 1,3-Azoles with Ozone

##### 3.2.3.1 General Mechanism

The observed products suggest that the ozonation mechanism of azoles has several pathways (Scheme 1). For all 1,3-azoles (**1**), the reactive center is the C=C double bond (reaction i), analogously to imidazole.^34^ The resulting primary ozonide **2** can cleave in two ways to form the corresponding zwitterions: reaction ii, pathway A (cyan/purple); and reaction viii, pathway B (red). The proportion of each pathway is likely determined by the ability of the resulting intermediates (**3** and **7**) to stabilize the positive charge (see below). In pathway A, intermediate **3** can undergo intramolecular cyclization to form the 5-membered ring **4** (reaction iii), which then rearranges, releasing (thio)­formate (reaction iv). The resulting carbonyl isocyanate **5** can hydrolyze at two positions: in pathway A1, hydrolysis of the carbonyl group forms the corresponding carboxylic acid and isocyanate (cyan, reaction v).^67^ In pathway A2 the isocyanate group is hydrolyzed to form the carbonyl-carbamic acid **6** (purple, reaction vi) which decarboxylates to form an amide and CO_2_ (reaction vii).^68^ This reaction is in agreement with the hydrolysis of acetyl-isocyanate (**5**) yielding acetamide and CO_2_.^69^ The formation of CO_2_ (same carbon-oxidation state +IV as cyanate in the other pathway), also explains the incomplete carbon mass balance, as CO_2_ was not determined. In pathway B, intermediate **7** can also cyclize intramolecularly to form the 5-membered ring **8** (reaction ix). This rearranges to cyanate and formic anhydride **9** (reaction x), hydrolyzing to formate and the corresponding (thio)­carboxylic acid (reaction xi). For thiazoles, the formed thiocarboxylic acids are further oxidized to the corresponding carboxylic acids and sulfate, releasing ^1^O_2_ in the process (Scheme 1, bottom).

**Scheme 1:**
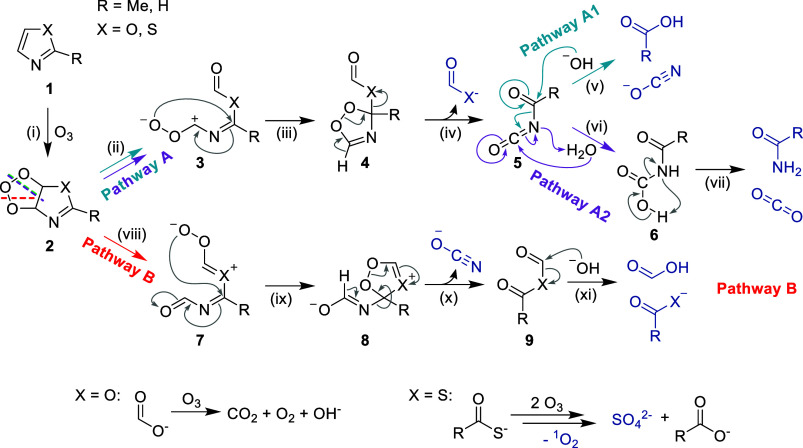
Proposed Reaction Mechanisms for the Reaction of 1,3-Azoles with Ozone based on the Identified and Quantified Reaction Products (Highlighted in Blue, according to Figures 1 and 2)­^a^

a The different reaction pathways are color-coded: pathway A1: cyan; pathway A2: purple; pathway B: red.

Overall, pathways A1 (cyan) and B (red) lead to the same final reaction products (two carboxylic acids, isocyanate and, in the case of thiazoles, sulfate). Pathway A2 (purple) results in a separate set of products (formic acid, the corresponding amide, CO_2_ and, in the case of thiazoles, sulfate).

##### 3.2.3.2 Influence of Methyl-Substitution at Position 2

For oxazole, ozonation yields only formate and cyanate (pathway A1 or B), while 2-methyloxazole mainly yields formate and acetamide, the product of pathway A2. The methyl substitution affects the product formation by changing the hydrolysis pathway of the isocyanate **5** (pathways A1, reaction v; and A2, reaction vi). Both types of hydrolysis, with attack on the carbonyl (pathway A1) or on the isocyanate (pathway A2), have been described previously.^67,68^ The kinetics of hydration reactions of carbonyl-groups strongly depend on the substitution, e.g. the hydrolysis rate of acetaldehyde is 2 orders of magnitude higher than for acetone.^70^ The hydrolysis of acetic formic anhydride (a structurally closely related compound to formyl isocyanate) is >100 times faster than for acetic anhydride (the analogue to acetyl isocyanate).^67^ From the two possible intermediates (**5**), formyl isocyanate (**5**, R = H) and acetyl isocyanate (**5**, R = Me), only the latter is described in the literature. Its reaction with water produces acetamide and CO_2_ (reaction vii), thus, the hydrolysis occurs at the isocyanate-carbon.^69^ Formyl isocyanate (**5**, R = H) has only been studied theoretically^71^ and the mechanism of its decay is therefore speculative. However, due to the much faster hydrolysis of the aldehydic carbonyl-group, hydrolysis at this position is plausible, leading to formate and cyanate (reaction vi). This explains that only formate and cyanate are the observed products of oxazole as the result of pathways A1 and B. In the case of 2-methyloxazole the methyl-group hinders hydrolysis (pathway A1) and acetamide and CO_2_ are formed (pathway A2).

##### 3.2.3.3 Influence of the Heteroatom at Position 1

The different yields of acetamide in the ozonation of 2-methyloxazole (89%) and 2-methylthiazole (26%) cannot solely be explained by the hydrolysis of acetyl isocyanate (**5**) (pathways A1 or A2) because the heteroatom X (O or S) is already cleaved off (reaction iv) and not part of intermediate **5**. Moreover, in the ozonation of 2-methylthiazole, thioacetate has been detected, which cannot be explained by pathway A, clearly indicating the existence of another reaction branch, pathway B. The different acetate and formate yields of 2-methyloxazole and 2-methylthiazole must therefore be explained by the stability of intermediates **3** and **7**. Due to the poor ability of oxygen to stabilize positive charges, especially compared to sulfur, pathway A is preferred for oxazoles, while pathway B is more pronounced for thiazoles.

##### 3.2.3.4 Ozonation of Imidazole

In a previous study, reaction pathway A1 was proposed for imidazole.^34^ According to our current knowledge, pathway B would be more likely, because nitrogen has a strong ability to stabilize positive charges. Nevertheless, the predicted products are the same for both pathways.^34^

#### 3.2.4 Reaction of 2-Methylthiazoline with Ozone

##### 3.2.4.1 ROS

2-Methylthiazoline (**S1**, Scheme 2) does not have a C=C double bond and its reactivity with ozone is different from aromatic thiazoles. This becomes evident in the ^1^O_2_ yield of 88% at a molar ratio of [**S1**]_0_:[ozone]_0_ = 1.1 (Table 2). Attempts to measure ^1^O_2_ yields at excess of substrate resulted in lower ^1^O_2_ yields, indicating possible ^1^O_2_ quenching by 2-methylthiazoline, as discussed in Section S10.1. Other ROS were observed with very low yields (Table 2).

**Scheme 2:**
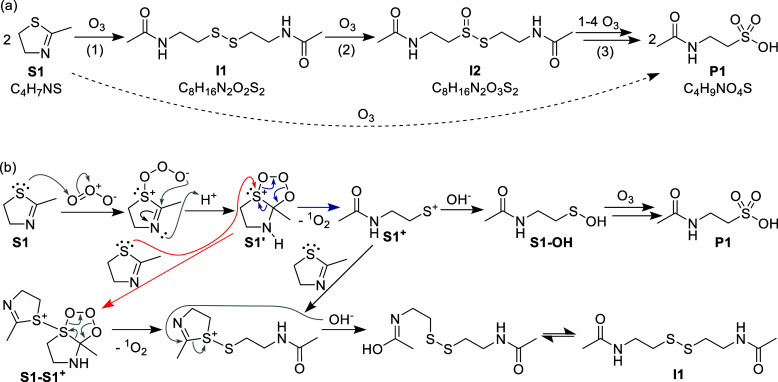
Reaction of 2-Methylthiazoline (**S1**) with Ozone; (a) Proposed Reaction Scheme to Disulfide **I1**, Which is Further Oxidized to the Organic Thiosulfinate Ester **I2** in a Second Step and Finally to *N*-Acetyltaurine (**P1**); (b) Proposed Detailed Reaction Mechanism for the Formation of **I1** (Bottom Pathways, Reaction (1)) and Possible Mechanism of the Formation of **P1**, without Dimerization (Blue, Upper Pathway)

##### 3.2.4.2 Quantification of Sulfur-Containing Products Using LC-ICP-MS/MS

During ozonation of 2-methythiazoline, none of the small, mostly ionic products of the organic azoles (see Sections 3.2.1 to 3.2.3) have been detected. In LC-UV-measurements (Figure S10.2a), two distinct product peaks were observed. To quantify the concentration of sulfur in the peaks, ozonated 2-methylthiazoline solutions were investigated by LC-ICP-MS/MS (in the following, all yields are based on the sulfur concentration). Apart from the substrate peak, and the previously found product peaks, two more sulfur-containing peaks were detected in the ozonated samples (an example chromatogram is shown in Figure S5.1b). Substrate **S1** is abated almost completely at a molar [ozone]:[**S1**] ratio of 1 (Figure 3a). A major (**I2**), a minor (**I1**), and a trace (**I3**) intermediate are formed. **I2** reaches a maximum yield of 76% at one equivalent of ozone, while **I1** has a maximum yield of 5% at 0.4 equiv of ozone (note that 2.1 ± 0.2% **I2** and 0.9 ± 0.4% of **I1** are present as impurities in the commercially available 2-methylthiazoline; see Section S5.5). Moreover, the trace intermediate **I3** has a maximum yield of 1.4% at molar [ozone]:[**S1**] ratio of 0.4–1.0. At higher ozone doses, all intermediates are fully consumed, leaving **P1** as the only final product (≥100% yield, Figure 3a).

**Figure 3:**
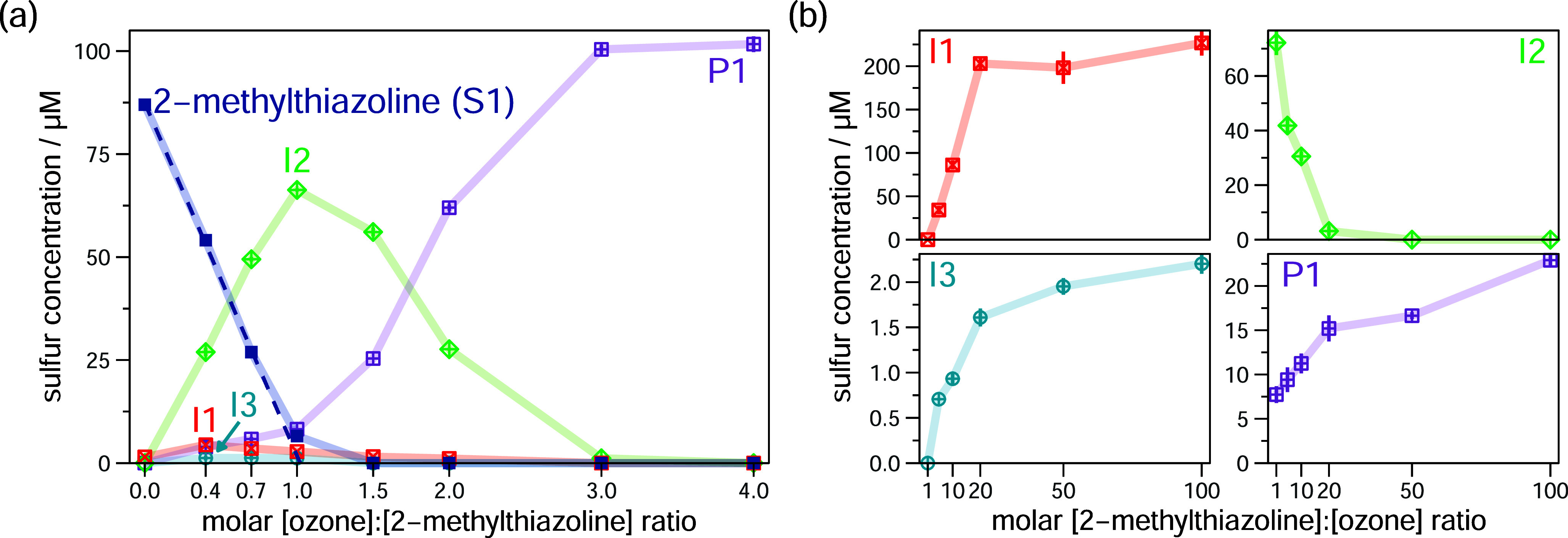
Abatement of 2-methylthiazoline during ozonation and formation of *S*-containing transformation products quantified by LC-ICP-MS/MS. (a) 2-Methylthiazoline abatement and formation of the intermediates **I1**, **I2** and **I3** and the final product **P1** (for chemical structures, see Scheme 2) as a function of the molar [ozone]:[**S1**] ratio in the presence of the ^•^OH scavenger *tert*-butanol. Experimental conditions: [2-methylthiazoline] = ∼0.1 mM; ozone doses = 0–0.4 mM; [*tert*-butanol] = 10 mM; [phosphate buffer (pH 7)] = 5 mM. Results are shown from a single experiment. Error bars represent the upper and the lower measured value of two injections (if not visible, the range falls within the symbol size). Ozone doses were not determined with a separate reaction vessel (see Section 2). The results of a replicate experiment with slightly different ozone doses are shown in Figure S10.3. (b) Product formation from the ozonation of 2-methylthiazoline as a function of the molar excess of 2-methylthiazoline for a constant ozone dose (0.1 mM). Results are shown as averages from triplicate experiments, error bars represent the corresponding standard deviations.

##### 3.2.4.3 Product Identification by LC-HRMS/MS and NMR

To elucidate the structures of **I1**, **I2**, **I3** and **P1**, their exact *m*/*z* values were measured by LC-HRMS/MS yielding the following sum formulas: C_8_H_16_N_2_O_2_S_2_ for **I1**, C_8_H_16_N_2_O_3_S_2_ for **I2**, C_8_H_14_N_2_O_3_S_2_ for **I3** and C_4_H_9_NO_4_S for **P1** (all data shown in Section S11: Figures S11.1, S11.3, S11.5 and S11.7). The sum formulas alone show that **I1**, **I2** and **I3** must be dimers, while **P1** is a highly oxidized monomer (the effect of the applied concentrations on dimerization is elucidated below). Attempts to analyze ^1^H NMR spectra of reaction solutions of ozonated 2-methylthiazoline revealed a large number of partially overlapping resonances that could not be analyzed in a target-oriented manner (data not shown). Therefore, a preparative-scale ozonation experiment with 2-methylthiazoline was performed and two clean products were isolated by preparative LC (Section S4.4). The LC-UV measurement of the two white solids matched with the compounds **I2** and **P1** (see Figure S10.2). NMR analysis of **I2** revealed resonances, corresponding to two closely related, yet distinct, 2-acetamidoethane species (complete NMR-data in Section S12.1). Given the sum formula, the compound must be the result of a dimerization and the two fragments must be connected by an asymmetric S_2_O-bridge like the thiosulfinic ester **I2** (Scheme 2a). MS^2^ spectra of **I2** support its suspected structure, notably the thiosulfinic ester fragment: 134.0276 (Figure S11.4).

The NMR data of the isolated compound **P1** were compared with those of commercially available *N*-acetyltaurine due to their identical molecular formula C_4_H_9_NO_4_S. All observed NMR resonances and 2D NMR cross signals aligned perfectly (Section S12.2). Therefore, **P1** is conclusively identified as *N*-acetyltaurine (Scheme 2). The annotation of the MS^2^ fragmentation pattern (Figure S11.9) confirmed the *N*-acetyltaurine structure and agreed well with the previously reported *N*-acetyltaurine fragmentation pattern.^72^ In the MS^1^ measurements the peak with *m*/*z* of 152.0377 corresponding to the sum formula C_4_H_10_NO_3_S could be attributed to the corresponding sulfinic acid of **P1** ([P1 – O + H]^+^, Figure S11.10). However, its retention time (Figure S11.8) and formation trend (Figure S11.11) matched with **P1**, suggesting that it could be a result of in-source fragmentation instead.

**I1** and **I3** have not been isolated for NMR characterization, due to their low yield at standard conditions (Figure 3a), and thus their structural elucidation solely relies on HRMS/MS data. The sum formula of **I1** corresponds to **I2** minus one oxygen (C_8_H_16_N_2_O_2_S_2_, see above). Comparison of MS^2^ spectra of **I1** and **I2** revealed the presence of similar fragments (Figures S11.2 and S11.4). The decisive exception is fragment 134.0276 which is attributed to the part of **I2** with the [O] addition on the sulfur and not found in the MS^2^ spectra of **I1**. Conversely, the presence of 150.0047 from **I1** fragmentation (Figure S11.2) suggests a nonoxidized S–S bond and is consistently not found in the MS^2^ spectra of **I2**. A disulfide dimer structure was therefore proposed for **I1** (Scheme 2a).

No structure for **I3** is proposed, but according to the sum formula C_8_H_14_N_2_O_3_S_2_, it must be a dimer of **S1** with three additional oxygen atoms (and no hydrolysis, unlike **I1** and **I2**). Fragmentation of **I3** shows similarities with **I1** and **I2**, but also distinctly other fragments are observed (Section S11.3 and Figure S11.6). No further effort was made to elucidate the structure due to it is low yield (Figure 3a).

##### 3.2.4.4 Elucidation of Dimerization

Dimerization reactions are less likely at lower concentrations and therefore abatement of **S1** and the yields of **I1**, **I2** and **P1** at different initial concentrations of **S1** as a function of the molar [ozone]:[**S1**] ratio were investigated (Figure S10.4a–d). Figure S10.4e displays the measured concentration of the four compounds at a molar [ozone]:[**S1**] ratio of 1 as a function of the initial concentration of **S1**. Decreasing the initial concentration of **S1** leads to a decrease of the formation of both dimers **I1** and **I2** (Figure S10.4e). This is expected because the formation of dimers is favored at higher substrate concentrations. Furthermore, decrease in product abatement was observed with decreasing initial **S1** concentration, which could be due to systematic errors in the dosing of small ozone doses. Interestingly, despite a lower substrate abatement, an increased yield of sulfonic acid **P1** was observed (Figure S10.4d,e, purple), suggesting a reaction path from **S1** to **P1** without formation of dimers (Scheme 2a, dashed arrow).

Ozonation experiments with increasing molar [**S1**]:[ozone] ratios (1–100) at a constant ozone dose also provide further mechanistic information on the first reaction step (Figure 3b). Increasing the molar [**S1**]:[ozone] ratios from 1 to 20 leads to an increase of the **I1** yield from 0% to 203%, based on the ozone dose (Figure 3b, upper left panel). This confirms that intermediate **I1** is a dimer, containing two sulfur atoms, which is formed from the reaction of two equivalents **S1** with one equivalent of ozone. Concurrently, the yield of **I2** decreased from 72% to 3%. Therefore, at large substrate excess, **I2** is no longer formed and must be a result of a second reaction step with ozone. In summary, this results in the reaction pathway shown in Scheme 2a, in which two **S1** react with one ozone to **I1** which in turn reacts rapidly with a second ozone to **I2**. Trace intermediate **I3** shows a similar pattern as **I1**, suggesting that it might also be formed by the reaction of two **S1** with one ozone (Figure 3b, bottom left panel). Furthermore, the final product **P1** is still formed at high substrate excess (Figure 3b, bottom right panel), confirming the existence of a pathway from **S1** (Scheme 2a, dashed arrow).

##### 3.2.4.5 Second-Order Rate Constant *k*_O_3_+**I2**_

To determine the second-order rate constant for the reaction of **I2** with ozone, a preozonation of 2-methylthiazoline was performed to yield a considerable amount of **I2**. Subsequently, the decrease of **I2** in molar excess of ozone was monitored (as described in Section S3.1). The determined second-order rate constant is *k*_O_3_+I2_ = 13 M^–1^s^–1^ (Table 1, entry 14), highlighting the high ozone-stability of **I2**.

##### 3.2.4.6 Reaction Pathway and Proposed Mechanism

Compiling the above information, a potential reaction pathway is proposed in Scheme 2a. The obtained data indicates that two 2-methylthiazoline (**S1**) initially react with one ozone to the disulfide **I1** (reaction (1)). This is consistent with the known tendency of sulfur compounds to form disulfides. Even though the simple oxygen transfer to **S1** to form the sulfoxide has not been observed (as it would be expected for thioethers),^11,28,29,73,74^ two factors indicate the initial attack of ozone on the sulfur: first, the relatively high second-order rate constant for the reaction of 2-methythiazoline with ozone (1.90 × 10^4^ M^–1^s^–1^, Table 1, entry 12), especially compared to the oxygen analogue 2-methyloxazoline (0.5 M^–1^s^–1^, Table 1, entry 11), and second, the high ^1^O_2_ yield of 88% (relative to the ozone dose).^11,28,29,73,74^ The nearly equimolar ^1^O_2_ yield at conditions, where oxidation of **S1** to **I2** is expected indicates that both reactions (1) and (2) produce ^1^O_2_ (Scheme 2a).

The reason for the C–S bond breakage and the formation of disulfide is not entirely clear but it can be hypothesized that an ozone attack on the sulfur forms an ozonide zwitterion which could form the primary ozonide **S1′** by intramolecular attack (Scheme 2b). The positively charged sulfur could lead to a nucleophilic attack from another **S1** (Scheme 2b, red arrow), forming the sulfonium ion **S1–S1** ^**+**^. After cleavage of ^1^O_2_ in a retro–1,3-dipolar cycloaddition, the ensuing hydrolysis leads to the disulfide **I1**. Alternatively, the primary ozonide **S1′** could already cleave off ^1^O_2_ to form a sulfur-cation **S1** ^**+**^ (Scheme 2b, blue arrow), which subsequently reacts with another **S1**, again followed by hydrolysis to **I1**.

The structural formula of trace intermediate **I3** has not been elucidated, but its formation trend is very similar to the formation trend of **I1**, both in the standard ozonation and in high excess of substrate (at a very low yield, Figure 3a, cyan, and 3b, bottom left panel). This suggests, it is likely formed by reaction of two moles of 2-methylthiazoline with one mole of ozone.

The disulfide **I1** in turn reacts further with ozone to form the thiosulfinate ester **I2** (reaction (2), Scheme 2a). Ozonation of disulfides have been reported to form thiosulfinate esters via oxygen transfer (^1^O_2_ yield of ca. 100%).^29^ The second-order rate constant for the reaction of **I1** with ozone was not determined, but *k*_O_3__ = ∼2 × 10^5^ M^–1^s^–1^ is reported for similar disulfides.^29^ This is 10-fold higher than for 2-methylthiazoline (*k*_O_3__ = 1.90 × 10^4^ M^–1^s^–1^, Table 1, entry 12) and explains the low yield of **I1** in standard conditions (Figure 3a). These finding are also in line with the measured ^1^O_2_ yield of 88% at conditions, where reaction (1) and (2) occur simultaneously.

Reactions of thiosulfinate esters with ozone (reaction (3), Scheme 2a) have not been investigated previously. The low second-order rate constant of *k*_O_3_+**I2**_ = 13 M^–1^s^–1^ explains the high stability of **I2**. The oxygen on the disulfide-group deactivates the nucleophilicity of the adjacent sulfur and decreases its reactivity by 4 orders of magnitude, compared to the reaction from **I1** to **I2**. With higher ozone doses, **I2** is oxidized to two equivalents of *N*-acetyltaurine (**P1**). Possible intermediates like sulfinic acids have second-order rate constants on the order of 10^6^ M^–1^s^–1^ ^75^ and outcompete the oxidation of thiosulfinate ester **I2**, as soon as they are formed. The abatement of **I2** (Figure 3a) suggests that several equivalents of ozone are necessary to fully oxidize **I2** to **P1**. However, with the low *k*_O_3_+**I2**_ the self-decay of ozone might influence the observed stoichiometry.

The formation pathway of **P1** from **S1**, indicated by its formation at low ozone doses and high excess of **S1**, could proceed through the hydrolysis of the sulfur-cation **S1** ^**+**^ to the sulfenic acid **S1–OH** (Scheme 2b, upper pathway). Sulfenic acids are very unstable in aqueous solution and can be readily oxidized by abundant dissolved oxygen to form first the sulfinic and subsequently the sulfonic acid.

### 3.3 Practical Implications

The reactivity of aromatic azoles during ozonation is strongly influenced by their substituents, and therefore, the efficiency of abatement of azole-containing compounds during ozonation can be highly variable. Based on the similar second-order rate constants for oxazole-containing compounds as for other micropollutants, they are expected to be effectively abated during ozonation.^76^ For a typical specific ozone dose of about 0.5 mg O_3_/mg DOC applied in municipal wastewater effluents, about 70% of metoprolol (*k*_O_3__ = 2 × 10^3^ M^–1^s^–1^, pH 7, similar to 2-methylthiazole, Table 2)^77^ was abated.^78^ For deactivated thiazoles (e.g., thiazole-4-carboxamide), with 2 orders of magnitude lower second-order rate constants (Table 1, entry 8), an abatement of about 30% can be expected in municipal wastewater effluent for typical ozone doses (reference compound atrazine, *k*_O_3__ = 6 M^–1^s^–1^).^78,79^ In such cases, the abatement is limited and controlled mostly by ^•^OH reactions.^37^

The identified reaction mechanisms show that ozonation of azole-type compounds causes the cleavage of all three ring carbons, leading to fragments with highly oxidized terminal groups, such as carboxylic acids and amides. It is expected that products such as formate, acetate, or cyanate are biodegradable during biological post-treatment, which is typical after ozonation.^35,80,81^

The direct ozone reaction with oxazoline-containing compounds during ozonation is likely limited and driven primarily by ^•^OH reactions. However, oxazoline has only 3 C-H bonds for ^•^OH attack, which also most likely leads to low *k.*_OH_ in the order of ≤3 × 10^9^ M^–1^s^–1^. Overall, a similar behavior to iopromide (*k*_O_3__ < 0.8 M^–1^s^–1^, *k.*_OH_ = 3 × 10^9^ M^–1^s^–1^)^76^ can be expected, which was abated by 10%–15% during secondary wastewater effluent ozonation (specific ozone dose 0.5 mg O_3_/mg DOC).^78^ In contrast, thiazoline-containing compounds are more reactive toward ozone, with similar expected abatement as 2-methyloxazole. Under realistic water treatment conditions, the significance of the observed dimerization reactions is unclear, as the concentrations of individual compounds are usually low. Other nucleophiles in solution, such as organic nitrogen compounds, may interact with the intermediates. Furthermore, the formation pathway of sulfonic acids could be more significant under these conditions.

## Supplementary Material



## Notes

Chemical structures of mentioned compounds; additional experimental details and analytical methods (LC-UV, IC-CD, LC-HRMS/MS, IC-HRMS, LC-ICP-MS/MS, and NMR), complementary experiments for the ozonation of oxazole-4-carboxamide, 2-methylthiazole and 2-methylthiazoline, which are not shown in the main manuscript, the elucidation of the ozonation mechanism of thioacetic acid, and LC-HRMS/MS- and NMR-data for the elucidation of the chemical structures of the identified transformation products (PDF)
