# Supplementary material for: Reactions of N,O- and N,S‑Azoles and -Azolines with Ozone: Kinetics and Mechanisms
Source: Environ Sci Technol. 2025 Jun 27;59(27):14103–15. doi: 10.1021/acs.est.5c01323 (PMC12269093; doi:10.1021/acs.est.5c01323)
Supplement: Supplementary file 1 [file es5c01323_si_001.pdf]

Supporting Information

Reactions of N,O- and N,S-azoles and -azolines with ozone: kinetics and mechanisms

Simon A. Rath[a,b], Valentin Rougé[a], Julie Tolu[a], Daniel Rentsch[c], Maria Lia Halder[a] and Urs von Gunten[a,b]*

[a] Eawag, Swiss Federal Institute of Aquatic Science and Technology, CH-8600 Dübendorf, Switzerland

[b] School of Architecture, Civil and Environmental Engineering (ENAC), Ecole Polytechnique Fédérale Lausanne (EPFL), CH-1015 Lausanne, Switzerland

[c] Empa, Swiss Federal Laboratories for Materials Science and Technology, CH-8600 Dübendorf, Switzerland

* Corresponding author: Urs von Gunten, vongunten@eawag.ch

This PDF includes:

12 sections in 57 pages, containing 32 Figures, 12 Tables and 5 Schemes, providing chemical structures of mentioned compounds, additional experimental details and analytical methods (LC-UV, IC-CD, LC-HRMS/MS, IC-HRMS, LC-ICP-MS/MS, and NMR), complementary experiments for the ozonation of oxazole-4-carboxamide, 2-methylthiazole and 2-methylthiazoline, which are not shown in the main manuscript, the elucidation of the ozonation mechanism of thioacetic acid, and LC-HRMS/MS- and NMR-data for the elucidation of the chemical structures of the identified transformation products.

Contents

S1 Published second-order rate constants of selected chemical compounds.................................. 4

S2 Reagents and solutions 5

S2.1 Chemicals.. 5

S2.2 Compound stock solutions 5

S2.3 Generation of ozone stock solutions... 5

S3 Determination of second-order rate constants 6

S3.1 Dispenser setup for low second-order rate constants 6

S3.2 Quench-flow setup for moderately high second-order rate constants 7

S3.3 Competition kinetics for very high second-order rate constants 7

S4 Ozonation experiments for target compound abatement and determination of transformation products... 9

S4.1 Standard ozone dosing experiments.. 9

S4.2 Ozonation of thiazole with varying excess of target compound................................. 9

S4.3 Ozonation of 2-methylthiazoline with varying concentration of ozone and target compound 10

S4.4 Preparative-scale ozonation of 2-methylthiazoline for identification by NMR 10

S5 Analytical methods 11

S5.1 LC-UV 11

S5.2 IC-CD 14

S5.3 LC-HRMS/MS 15

S5.4 IC-HRMS/MS 16

S5.5 LC-ICP-MS/MS 17

S5.6 NMR spectroscopy 21

S6 Ozonation of thiazole in excess of substrate.. 22

S7 Reaction of oxazole-4-carboxamide with ozone 23

S8 Reaction of 2-methylthiazole with ozone: detection of thioacetate, thioformate with LC-HRMS and an intermediate with IC-CD and IC-HRMS.. 25

S9 Reaction of thioacetate with ozone 27

S10 Additional experiments on the reaction of 2-methylthiazoline with ozone 32

S10.1 Measurement of singlet oxygen ($^1O_2$) yield for the ozonation of 2-methylthiazoline at different molar ratios 32

S10.2 LC-UV Chromatograms of the ozonation of 2-methylthiazoline 34

S10.3 Duplicate experiment of the reaction of 2-methylthiazoline with ozone 35

S10.4 Ozonation of 2-methylthiazoline with varying initial concentrations of substrate and ozone 36

S11 LC-HRMS/MS-data for product identification of 2-methylthiazoline...................................... 37

S11.1 LC-HRMS/MS data of **I1** 37

S11.2 LC-HRMS/MS data of **I2** 40

S11.3 LC-HRMS/MS data of **I3** 42

S11.4 LC-HRMS/MS data of **P1** (*N*-acetyltaurine) and co-eluting substances............................ 44

S12 NMR-data for the identification of compounds **I2** and **P1** 48

S12.1 NMR data of **I2** (S-(2-acetamidoethyl) 2-acetamidoethane-1-sulfinothioate)................. 48

S12.2 NMR data of **P1** (N-acetyltaurine) 50

S13 References... 54

S1 Published second-order rate constants of selected heterocyclic compounds for the reaction with ozone

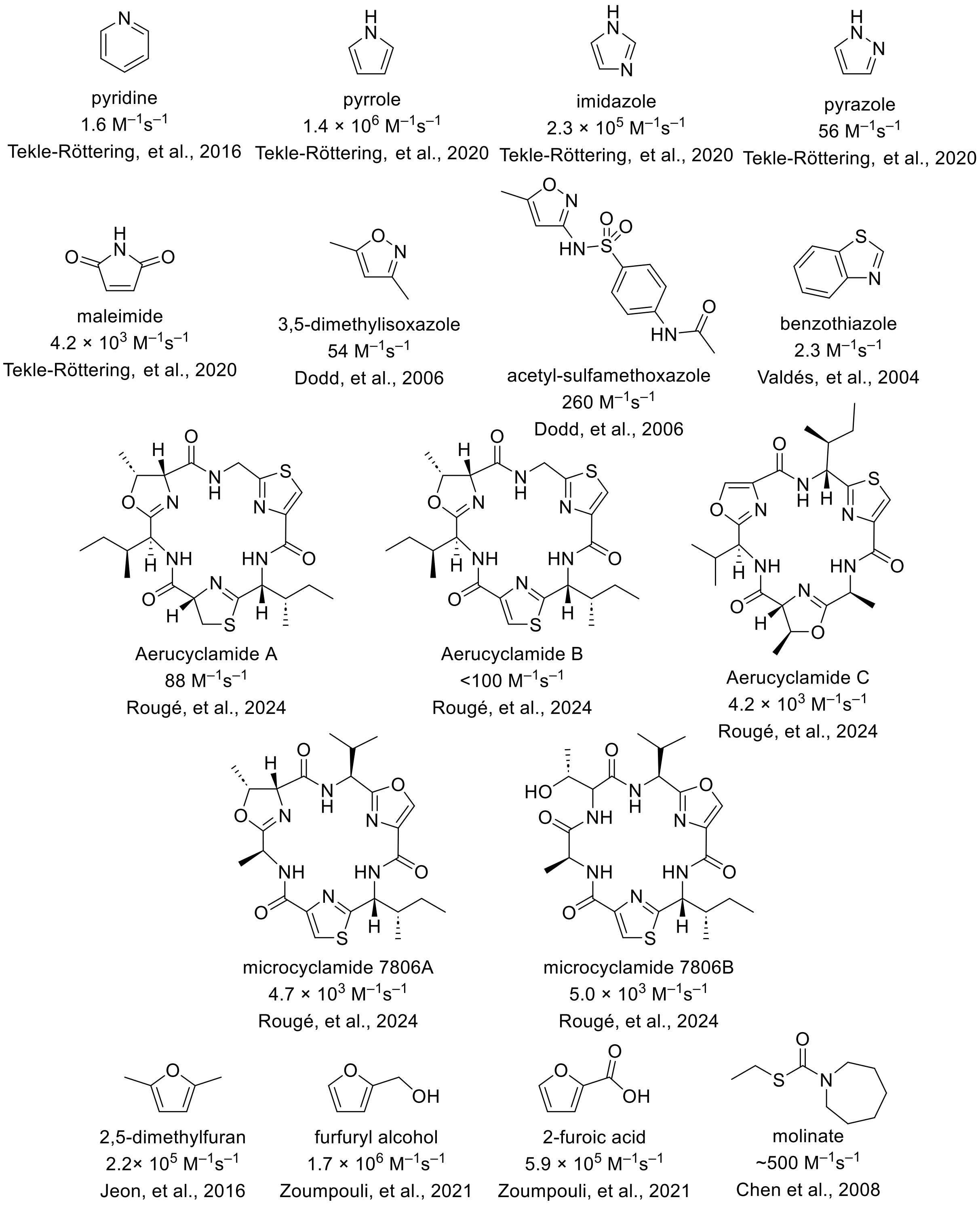

Scheme S1.1 – Chemical structures and the corresponding second-order rate constants of compounds (with references), discussed in Sections 1 and 3.1 of the main manuscript.[1–8]

S2 Reagents and solutions

S2.1 Chemicals

Sodium hydroxide (≥98%), sodium phosphate dibasic dihydrate (≥98%), sodium phosphate monobasic monohydrate (99%), phosphoric acid (≥85%), sodium carbonate (≥ 99.5%), trans-cinnamic acid, benzaldehyde (>99.5%), potassium indigotrisulfonate, *tert*-butanol (≥99.7%), sodium hypochlorite solution (6–14% active chlorine basis), hydrogen peroxide solution (purum p.a., ≥35%), 1,3-thiazole-4-carboxamide (95%), 2-methyl-1,3-thiazole-4-carboxamide (99.9%), 2-methyl-1,3-oxazole (95%), thiazole-4-carboxaldehyde (95%), oxazole-4-carboxamide (97%), 2-methyloxazole-4-carboxamide (95%), 4-oxazolecarboxaldehyde (97%), acetamide (>99%), thioacetic acid (96%), 2-(acetylamino)-ethanesulfonic acid (95%), sodium cyanate (96%), formate standard for IC (1000 mg/L ± 5 mg/L), acetate standard for IC (1000 mg/L nominal concentration), sulfate standard for IC (1000 mg/L ± 4 mg/L), nitrate standard for IC (1000 mg/L ± 4 mg/L) were obtained from Sigma-Aldrich (Switzerland).

Methanol (99.99%) LC/MS grade was obtained from Thermo Fisher Scientific (Switzerland).

2-Methylthiazoline (>98%), 2-methylthiazole (>98%), and 2-methyl-2-oxazoline (>98%) were obtained from TCI Chemicals (Japan), 2-methyl-1,3-oxazole (95%) was obtained from Enamine (Ukraine).

All aqueous solutions were prepared in ultrapurified water with a resistivity of >18.2 MΩ·cm (Arium® pro ultrapure water systems, Sartorius AG, Goettingen, Germany).

Hypochlorite solution was standardized spectrophotometrically (ε_{290nm} = 350 $M^{-1}cm^{-1}$).[9]

S2.2 Compound stock solutions

Compounds were dissolved in ultrapurified water at concentrations between 1 and 1000 mM. The solutions were used no longer than four weeks. For 2-methylthiazoline and thioacetic acid, the solutions were prepared freshly for every experiment. 1 mM indigo solution containing 0.1 vol% of phosphoric acid were stored at 4°C in the dark, and prepared freshly at least every 4 weeks.

S2.3 Generation of ozone stock solutions.

Ozone (O_3) stock solutions (1.6–1.9 mM) were prepared by sparging ozone-containing oxygen gas into ice cooled ultrapurified water.[10,11] Ozone was produced by an ozone generator (BMT 803 BT, BMT Messtechnik, Berlin) from pure oxygen (Carbagas, 99.995%). The ozone concentration of the stock solution was determined by direct UV absorbance at 260 nm (ε = 3200 $M^{-1}cm^{-1}$).[11]

S3 Determination of second-order rate constants

The methods for the determination of the second-order rate constants were chosen according to multiple factors: If a reaction stoichiometry ozone:azole of one and a high second-order rate constant (k > 10^3 $M^{-1}s^{-1}$) was expected, quench-flow with excess target compound was used, while lower second-order rate constants (k < 10^3 $M^{-1}s^{-1}$) and reactions with reaction stoichiometries ozone:azole > 1 were determined in a dispenser setup with excess of ozone (as described below). To reach a sufficient ozone concentration to measure the decrease of 2-methyloxazoline, a different preparation of the reaction solution was applied (see below). All kinetic experiments were performed at least in duplicate at 23±1 °C with 10 mM *tert*-butanol to quench hydroxyl radicals (•OH) at pH 7.0±0.1 in 10 mM phosphate buffer.

S3.1 Dispenser setup for low second-order rate constants

For measurement of low to moderate second-order rate constants (k_{O_3} < 10^3 $M^{-1}s^{-1}$), pseudo first-order conditions with a ten-fold excess of ozone were applied. A 250 mL glass bottle equipped with a bottle-top dispenser[12] was used as reaction vessel. Ozone stock solution was added to the target compound solution, containing 10 mM phosphate buffer (pH 7) and 10 mM *tert*-butanol. 1.5 mL samples were withdrawn through the dispenser and quenched with cinnamic acid (minimum 5-fold molar excess compared to ozone). The residual ozone concentration was determined by quantifying benzaldehyde, the reaction product of ozone with cinnamic acid, via LC-UV and the decrease of the target compound was also followed by LC-UV. Second-order rate constants were obtained by plotting the relative logarithmic residual concentration of the target compound ln(TC/TC_0) as a function of the ozone exposure $\int[O_3]dt$. The negative slope of the linear regression corresponds to the second-order rate constant. All experiments were conducted in triplicates.

$$\ln\left(\frac{[TC]}{[TC]_0}\right) = -k_{O_3+T,app}\int [O_3]\, dt$$

Very high ozone doses were required for the experiment with 2-methyloxazoline, due to its low second-order rate constant for the reaction with ozone. Therefore, the ozone, produced by an ozone generator (see above) was sparged through a 10 mM *tert*-butanol solution with 10 mM phosphate buffer (pH 7) until a concentration of $[O_3]$ = 1 mM was reached. The ozone concentration was followed continuously by an optical fiber (Avantes/AvaSpec-ULS2048CL-EVO) that was immersed in the ozone reactor.[13] The ozone sparging was stopped and 0.03 mM of 2-methyloxazoline were added. The reaction vessel was equipped with a dispenser and 1.5 mL were withdrawn at several time points (15s to 30min) and quenched with 0.1 mL of a 100 mM sodium sulfite solution. The residual concentration of methylthiazoline was determined by LC-UV and the ozone exposure $\int[O_3]dt$ from the measured data of the UV probe. The second-order rate constant was calculated as described above.

S3.2 Quench-flow setup for moderately high second-order rate constants

For measurement of moderately high second-order rate constants (k_{O_3} = 10^3 to 10^5 $M^{-1}s^{-1}$), the quench-flow system Biologic SFM-400/Q was used.[14] This system allows fast mixing of the reaction solutions via drive syringes. The speed of the syringes and the delay line determine the reaction time. The three syringes of the quench-flow system contained (i) a solution of the target compound, 10 mM phosphate buffer (pH 7) and 10 mM *tert*-butanol, (ii) an ozone stock solution, stabilized at pH 4 with hydrochloric acid*, and (iii) an indigo solution. For the reaction, ten parts of the reaction solution (i) were mixed with one part of the ozone solution (ii) and pushed through the delay line. After the delay line, the indigo quenching solution (iii) is mixed with the reaction solution to quench the residual ozone. The resulting mixture was collected and the ozone concentration was determined by the decolorization of indigo, measured spectrophotometrically at 600 nm (ε_{600nm} = 22,070 $M^{-1}cm^{-1}$), compared to blank samples.[11,15] Every time point was measured at least twice. The observed pseudo first-order rate constant k_{obs} was determined from the slope of a linear regression of $\ln\left(\frac{[O_3]}{[O_3]_0}\right)$ vs t. Division by the concentration of the target compound leads to the second-order rate constant.

$$\ln\left(\frac{[O_3]}{[O_3]_0}\right) = -k_{O_3+TC,app}[TC] \times t = -k_{obs} \times t$$

* *Comment: The addition of hydrochloric acid has been taken over from a previous internal procedure. In retrospect, the addition of hydrochloric acid is not ideal, as it has the potential to react with $^{\bullet}OH$ and form a variety of other reactive species.[11] The second-order rate constant of this reaction is highly pH-dependent. At pH 4, it is $k_{\bullet OH+Cl^-}$ = 10^6 $M^{-1}s^{-1}$, while the second-order rate constant of ozone with $^{\bullet}OH$ is $k_{\bullet OH+ozone}$ = 10^8 $M^{-1}s^{-1}$.[11] Therefore, at similar concentrations in the ozone stock solution ([HCl] = 0.1 mM; [O_3] = 0.1 – 0.5 mM), a maximum of 1% of formed $^{\bullet}OH$ could react with chloride. The reaction of $^{\bullet}OH$ with Cl- forms chlorine radicals which react with ozone to chlorine monoxide radicals and other reactive chlorine species. This chemistry may become important at low pH. Thus, under the applied experimental conditions, changes in the outcome of these quench-flow experiments can be excluded. For that reason, the experiments have not been repeated. However, for pH << 4, this choice would have been problematic. In general, a better choice would have been sulfuric or phosphoric acid.*

S3.3 Competition kinetics for high second-order rate constants

For high second-order rate constants (k_{O_3} > 10^5 $M^{-1}s^{-1}$, only thioacetate in this study), competition kinetics with cinnamic acid was applied, following the abatement of target compound (TC, thioacetate)

and competitor (C) with different ozone doses as described previously (Dodd et al., Method IV)[3]. The apparent second-order rate constant was determined using the measured pH and the following species-specific second-order rate constants: $k_{O_3+\text{cinnamate}}$ = 7.6 × 10^5 $M^{-1}s^{-1}$, $k_{O_3+\text{cinnamic acid}}$ = 5.8 × 10^4 $M^{-1}s^{-1}$.[16,17] Different ozone dose (0.01 – 0.16 mM) were added to nine rapidly stirred mixtures of target compound and competitor cinnamic acid (20 mL, each 0.1 mM of T and C), containing 15 mM *tert*-butanol and 10 mM phosphate buffer (pH 7). The residual concentration of cinnamic acid was measured by LC-UV and the residual concentration of thioacetate was determined by IC-CD (see below). The second-order rate constant was determined from the slope of the plot of $\ln\left(\frac{[TC]}{[TC]_0}\right)$ vs $\frac{\ln\left(\frac{[C]}{[C]_0}\right)}{k_{O_3+C,app}}$. $[TC]_0$ and $[C]_0$ were determined by analyzing duplicate controls with $[O_3]$ = 0 mM.

$$\ln\left(\frac{[TC]}{[TC]_0}\right) = k_{O_3+TC,app} \times \frac{\ln\left(\frac{[C]}{[C]_0}\right)}{k_{O_3+C,app}}$$

S4 Ozonation experiments for substrate abatement and determination of transformation products

S4.1 Standard ozone dosing experiments

Ozonation experiments for the quantification of the abatement of substrate and the formation of transformation products were set up as follows: Solutions with the following concentrations were used: [azole] = ~0.1 mM; [*tert*-butanol] = 10 mM; [phosphate buffer (pH 7)] = 5 mM. The *tert*-butanol concentration was chosen, that >95% of formed hydroxyl radicals react with *tert*-butanol. 20 mL of the solutions were prepared and aqueous ozone was dosed (0–0.5 mM) to the reaction solution from a stock solution (1.6–1.9 mM). To determine the actual ozone dose as accurately as possible, for every specific ozone dose, a separate 20 mL reaction vessel with 2.5 mM cinnamic acid and 400 mM *tert*-butanol, to suppress any •OH reactions, was ozonated (unless stated otherwise), as described previously.[16] The product of the reaction of ozone and cinnamic acid, benzaldehyde, was quantified by LC-UV.

The reaction solutions of oxazole and thiazole were analyzed by LC-UV for the abatement of substrate and in a 1:5 dilution for IC-CD for the quantification of the ionic transformation products.

The reaction solutions of 2-methyloxazole and 2-methylthiazole were analyzed by LC-UV for the abatement of substrates, by LC-HRMS/MS for the quantification of acetamide, and in a 1:5 dilution for IC-CD for the quantification of the ionic transformation products.

The reaction solutions of 2-methylthiazoline were analyzed by LC-ICP-MS/MS for the quantification of the substrate and the sulfurous transformation products. In this experiment, a phosphate buffer concentration of [phosphate buffer (pH 7)] = 10 mM was used and the specific ozone dose was not explicitly measured with cinnamic acid but was calculated from the determined concentration of the ozone stock solution and the dosed volume.

S4.2 Ozonation of thiazole with varying excess of substrate

To elucidate the sulfate formation, an experiment with constant ozone dose ([ozone] = 0.1 mM), but varying concentrations of thiazole ([thiazole] = 0.2–10 mM) was conducted ([*tert*-butanol] = 10 mM; [phosphate buffer (pH 7)] = 5 mM). *tert*-butanol concentration was kept constant at 10 mM, although at higher concentration of thiazole, more than 5% of •OH could react with thiazole. Nevertheless, this reaction is negligible, as at these high thiazole concentrations, the self-decay of ozone does not play a significant role and the reaction of ozone with thiazole does not produce •OH. The experiment was analyzed by IC-CD to quantify the reaction products. The results are shown in Section S6.

S4.3 Ozonation of 2-methylthiazoline with varying concentration of ozone and substrate

Two additional ozone dosing experiments were conducted:

1) Experiment to elucidate the role of the dimerization reaction
2) Experiment to isolate the first step of the reaction

1) To elucidate the role of the dimerization reaction, a dosing experiment similar to the one described in Section S4.1 was conducted with the difference that the initial concentration of 2-methylthiazoline was varied: $[\text{2-methylthiazoline}]_0$ = 5–100 µM. The required ozone doses were adjusted proportionally. All experiments were conducted with [*tert*-butanol] = 10 mM; [phosphate buffer (pH 7)] = 10 mM.

2) To gather more information about the first step of the reaction, an experiment with constant ozone dose ([ozone] = 0.1 mM), but varying concentrations of 2-methylthiazoline ([2-methylthiazoline] = 0.1–10 mM) was conducted. *tert*-butanol concentration was kept constant at 10 mM, although at higher concentration of 2-methylthiazoline, more than 5% of $^{\bullet}$OH could react with thiazole. Nevertheless, this reaction is negligible, as at these high 2-methylthiazoline concentrations, the self-decay of ozone does not play a significant role and the reaction of ozone with thiazole does not produce $^{\bullet}$OH.

Both experiments were analyzed by LC-ICP-MS/MS to quantify the sulfurous reaction products. The results are shown in Section S8.

S4.4 Preparative-scale ozonation of 2-methylthiazoline for identification by NMR

For the NMR-analysis of the products of 2-methylthiazoline, preparative-scale experiments were conducted. 1L of an ozone stock solution (1.5 mM) was added slowly to 100 mL of a 15 mM solution of 2-methylthiazoline (without any additives). After an appropriate time for full ozone depletion, the resulting solution was concentrated with a rotary evaporator to a volume of about 20 mL and the resulting mixture was separated by reversed-phase LC, injecting 100 µL per run (ca. 100 runs, method A, Table S5.1). Two fractions were collected and after evaporation of the solvents, two white solids were isolated. These solids were analyzed by LC-UV (Chromatograms of the fractions: Figure S10.2b, and c, respectively) and NMR (Section S12).

S5 Analytical methods

S5.1 LC-UV

All analyses were performed by a LC (Ultimate 3000, Thermo) equipped with diode array detector (DAD) with a COSMOSIL 5C18-MS-II column (3.0 × 150 mm, 5 µm) equipped with a pre-column using the following methods at a flow-rate of 0.6 mL/min (except method F, Table S5.6). 100 µL of samples were injected. The used methods are described in Tables 5.1 to 5.7.

Table S5.1 – LC method A for quantification of some azoles (see Table S5.8).

Time / min	Eluent A<br>MeOH	Eluent B<br>10 mM $(NH_4)HCO_3$
0	5%	95%
7	5%	95%
15	80%	20%
17	5%	95%
20	Stop run	

Table S5.2 – LC method B of some azoles and benzaldehyde (see Table S5.8).

Time / min	Eluent A<br>MeOH	Eluent B<br>10 mM $(NH_4)HCO_3$
0	5%	95%
3	5%	95%
10	90%	10%
12	5%	95%
15	Stop run	

Table S5.3 – LC method C for benzaldehyde / cinnamic acid (see Table S5.8).

Time / min	Eluent A<br>MeOH	Eluent B<br>10 mM H_3PO_4
0	40%	60%
0.5	40%	60%
2.5	60%	40%
10	60%	40%
11	40%	60%
12	Stop run	

Table S5.4 – LC method D for quantification of 2methyloxazoline (see Table S5.8).

Time / min	Eluent A<br>MeOH	Eluent B<br>10 mM H_3PO_4
0	5%	95%
8	Stop run	

Table S5.5 – LC method E for quantification of methylthiazoline in isocratic conditions. The gradient is used to flush out nonpolar impurities at the end of the method (see Table S5.8).

Time / min	Eluent A<br>MeOH	Eluent B<br>10 mM $(NH_4)HCO_3$
0	15%	85%
13	15%	85%
15	80%	20%
17	80%	20%
17.5	15%	85%
20	Stop run	

Table S5.6 – LC method F for LC-HRMS/MS of ozonated samples of 2-methylthiazoline. The flow rate was 0.3 mL/min (see Table S5.8).

Time / min	Flow / mL/min	Eluent A<br>MeOH	Eluent B<br>10 mM $(NH_4)HCO_3$
0	0.3	5%	95%
17	0.3	5%	95%
40	0.3	50%	50%
42	0.3	100%	100%
47	0.3	100%	100%
50	0.3	5%	95%
65		Stop run	

Table S5.7 – LC method G for quantification of 2,4-dinitrophenylhydrazone (see Table S5.8).

Time / min	Eluent A<br>MeCN	Eluent B<br>Water
0	45%	55%
12	Stop run	

Table S5.8 – Compounds analyzed by LC-UV and the corresponding analytical conditions, LOQs and measuring ranges. n.d.: not determined.

Compound	Detection wavelength / nm	Method	RT / min	LOQ / µM	Measuring range / µM
oxazole	205	A (Table 5.1)	3.6	1	1 – 100
2-methyloxazole	205	A (Table 5.1)	8.0	1	1 – 100
oxazole-4-carboxamide	237	B (Table 5.2)	2.9	0.2	0.2 – 1
oxazole-4-carboxaldehyd	237	B (Table 5.2)	2.9	0.1	0.1 – 1
2-methyl-1,3-oxazole-4-carboxamide	n.d.	C (Table 5.3)	2.2	n.d.	n.d.
thiazole	237	A (Table 5.1)	6.6	2	2 – 100
2-methylthiazole	237	A (Table 5.1)	12.8	0.2	0.2 – 100
thiazole-4-carboxamide	237	B (Table 5.2)	4.9	0.1	0.1 – 20
thiazole-4-carboxaldehyd	250	B (Table 5.2)	4.9	0.5	0.5 – 20
2-methyl-1,3-thiazole-4-carboxamide	237	B (Table 5.2)	8.0	0.05	0.05 – 5
2-methyloxazoline	210	D (Table 5.4)	6.6	2	2 – 25
2-methylthiazoline	205	A (Table 5.1)	13.4	n.d.	n.d.
	205	E (Table 5.5)	11.3	5	5 – 100
I2	205	A (Table 5.1)	7.5	n.d.	n.d.
	205	E (Table 5.5)	2.6	n.d.	n.d.
P1	205	A (Table 5.1)	1.8	n.d.	n.d.
	205	E (Table 5.5)	1.6	n.d.	n.d.
	205	F (Table 5.6)	3.2	n.d.	n.d.
cinnamic Acid	n.d.	B (Table 5.2)	9.0	n.d.	n.d.
	275	C (Table 5.3)	6.5	<0.1	0.1 – 100
benzaldehyde	250	B (Table 5.2)	10.3	1	1 – 10
	250	C (Table 5.3)	4.8	0.1	0.1 – 40
2,4-dinitrophenylhydrazone	360	G (Table 5.7)	6.1	0.5	0.5 – 100

S5.2 IC-CD

For anion quantification, samples were diluted with ultra-purified water (1:5) to reduce column overloading from phosphate buffer. 100 µL samples were injected and analyzed by ion chromatography (IC) coupled with conductivity detection (CD) (Dionex™ Integrion™ HPIC™ System) using an anion exchange IC column (Dionex™ IonPac™ AS19-4µm IC Column 2 × 250mm with AG19-4 µm Guard Column 2 × 50mm). The eluent conditions (gradient of KOH) are shown in Table S5.9 and details of detectable anions are provided in Table S.5.10. A dynamically regenerated suppressor (Dionex™ DRS 600, Thermo Scientific™) was used at appropriate values of suppressor current to exchange cations by H^+ and thereby neutralizing the basic eluent.

Table S5.9 – IC method.

time	KOH / mM
0	10
10	10
30	30
30.1	100
37	100
37.1	10
42	Stop run

Table S5.10 – Compounds analyzed by IC-CD and the corresponding analytical condition, LOQ (absolute value) and measuring ranges.

Ion	RT / min	LOQ / µM	Measuring range / µM
acetate	6.0	0.02	0.1–20
formate	6.8	0.01	0.1–30
intermediate*	7.8	n.d.	n.d.
thioacetate	11.8	0.02	0.1–20
cyanate	12.7	0.04	0.1–20
nitrate	16.7	0.02	0.1–0.5
sulfate	21.5	0.01	0.1–20

* This intermediate is observed in the reaction of ozone with the substrates 2-methylthiazoline or thioacetate. For more information see Sections 3.2.2 (main manuscript), S8 (Figure S8.3) and S9 (Figures S9.2 and S9.3)

S5.3 LC-HRMS/MS

LC-HRMS/MS analyses were performed with a LC (Dionex Ultimate 3000 RS pump, Thermo Fischer Scientific) with a COSMOSIL 5C18-MS-II column (3.0 × 150 mm, 5 µm), coupled to a high-resolution tandem mass spectrometer (HRMS/MS, Orbitrap Exploris 240, ThermoFisher Scientific). 100 µL were injected and eluted using 10 mM ammonium bicarbonate solution and methanol.

2-Methyloxazole / 2-methylthiazole: A gradient elution was carried out, described in the previous section (method A, Table S5.1). HRMS/MS used electrospray ionization (ESI) with 320°C capillary temperature, and both positive and negative ionization modes with 3.5 kV and 2.5 kV electrospray voltage, respectively. Full scan from 40 to 500 *m/z* was used with a nominal resolution of 120000 at *m/z* 250, 1×10^6 automated gain control (AGC), 50 ms maximal injection time, and 1 ppm mass accuracy. Internal mass calibration was done on each run start (EASY-IC) to prevent mass drift overtime. Top 3 high-resolution MS^2 product ion spectra were obtained by normalized collision energies for HCD of 30%, 70%, 90% at a resolving power of 30000 at 400 *m/z*, 5×10^4 AGC, 70 ms maximal injection time, 2 *m/z* isolation window.

2-Methylthiazoline: A gradient elution was carried out, described in the previous Section (method E, Table S5.5; or Method F, Table S5.6). HRMS/MS used electrospray ionization (ESI) with 320°C capillary temperature, and both positive and negative ionization modes with 3.5 kV and 2.5 kV electrospray voltage, respectively. Full scan from 50 to 750 *m/z* was used with a nominal resolution of 120000 at *m/z* 250, 1×10^6 automated gain control (AGC), 50 ms maximal injection time, and 1 ppm mass accuracy. Internal mass calibration was done on each run start (EASY-IC) to prevent mass drift overtime. Top 3 high-resolution MS^2 product ion spectra were obtained by normalized collision energies for HCD of 50%, 70%, 90% at a resolving power of 15000 at 400 *m/z*, 5×10^4 AGC, 70 ms maximal injection time, 2 *m/z* isolation window.

Peak integration and structural identification: Skyline 22.2 (MacCoss LabSoftware) was used for the integration of the peaks. MS^1 filtering settings were the same as the measurement settings, i.e., *m/z* scan range between 40 and 500, and resolving power of 120000 at *m/z* 250.

The cumulated integration of the peak areas of up to M+3 isotopes was used. Structural identification of the products from the ozonation of 2-methyloxazole, 2-methylthiazole and 2-methylthiazoline was done by annotating fragments from the generated MS^2 spectra (Figures S11.2, S11.4, S11.6, S11.9 and S11.10) with the assistance of MetFrag Web.[18]

S5.4 IC-HRMS/MS

IC-HRMS/MS analyses were performed with a IC (ICS-5000+ system, Thermo Scientific™) with a Dionex™ IonPac™ AS19-4 µm column (2 × 250 mm) equipped using a 2 × 50 mm guard column of the same specifications, coupled to a high-resolution tandem mass spectrometer (HRMS/MS, QExactive Plus, ThermoFisher Scientific). Samples were diluted with ultra-purified water (1:5) to reduce column overloading from phosphate buffer. 100 µL were injected. The eluent conditions (gradient of KOH) are shown in Table S5.9. A dynamically regenerated suppressor (Dionex™ DRS 600, Thermo Scientific™) was used at appropriate values of suppressor current to exchange cations by H^+ and thereby neutralizing the basic eluent before entering the MS. The IC was run in external water mode, such that the suppressor was regenerated by a regeneration flow of 0.5 mL/min ultrapure water, delivered by an external pump (Ultimate 3000, RS Pump, Dionex™, Thermo scientific™). To protect the MS, the electrical conductivity was monitored between the suppressor and addition of the make-up flow. A trigger was programmed to stop flow to the MS in case the conductivity was higher than 10 µS/cm for longer than 240 s. The aforementioned external pump also delivered the make-up flow of 0.2 mL/min ethanol before entering the MS, resulting in 0.55 mL/min flow to the MS.

HRMS/MS used electrospray ionization (ESI) with 320°C capillary temperature, and negative ionization modes with 3.5 kV and 2.5 kV electrospray voltage, respectively. Full scan from 50 to 500 *m/z* was used with a nominal resolution of 140000 at *m/z* 250, 1×10^6 automated gain control (AGC), 50 ms maximal injection time, and 1 ppm mass accuracy. Top 5 high-resolution MS^2 product ion spectra were obtained by normalized collision energies for HCD of 30%, 70%, 90% at a resolving power of 30000 at 400 *m/z*, 5×10^4 AGC, 70 ms maximal injection time, 2 *m/z* isolation window. Skyline 22.2 (MacCoss LabSoftware) was used for the integration of the peaks. MS^1 filtering settings were the same as the measurement settings, i.e., *m/z* scan range between 50 and 500, and resolving power of 140000 at *m/z* 250.

S5.5 LC-ICP-MS/MS

An Infinity 1260 Bioinert LC (Agilent) coupled to an inductively coupled plasma tandem mass spectrometer (ICP-MS/MS; Agilent 8900) was used to detect and quantify 2-methylthiazoline (**S1**) and the **S1**-ozone reaction products by adapting operating conditions from previous studies.[19,20]

The ICP-MS/MS functioned with an argon plasma (RF power, 1550 W; plasma gas, 15 L/min; auxiliary gas, 0.9L/min), and was equipped with a quartz torch (1 mm i.d.) and platinum skimmer and sampler cones. To obtain sharp peaks (and thus increase the sensitivity of the detection of sulfur-containing transformation products), we used a PFA zero dead volume LC-nebulizer (Elemental Scientific, Omaha, NE, USA).[20] To remove carbon coming from the mobile phase (MeOH gradient) in the plasma and thus prevent soot deposition on the sample cone that results in important sensitivity suppression, oxygen (20% O_2 in Ar) was added to the carrier gas to reach i) 10% of total carrier gas flow for experiments shown in Fig. 3a and S10.4 (the LC method constantly used 15% MeOH); and ii) 20% of total carrier gas flow for experiments shown in Fig. 3b (the LC method involved a cleanup and reconditioning step up to 80% MeOH going to the plasma, as described below). As commonly done in previous studies (e.g., Balcaen et al. 2013, Tolu et al. 2022),[19,21] sulfur was detected in MS/MS mode using 30% O_2 in the collision/reaction cell (S reacts with O to form SO), with *m/z* 32 (MS^1) →48 (MS^2) and an acquisition time of 0.05 min. To check for stable sensitivity through the chromatographic separation and the run, an internal standard containing scandium (Sc), rhodium (Rh), and lutetium (Lu) was added post-column (just before the ICP-MS/MS nebulizer) using a T-piece and the peristaltic pump of the ICP-MS/MS (concentrations reaching the ICP-MS/MS were of 80 µg/L for Sc and 8 µg/L for Rh and Lu). These elements were detected with *m/z* 45→61 (Sc), *m/z* 103→103 (Rh), and *m/z* 175→191 (Lu) (acquisition time of 0.05 min).

As for the LC-UV and LC- HRMS/MS analysis, the chromatographic separation was performed using a COSMOSIL C18 column (3.0x100 mm, 5.0 µm), with an injection volume of 100 µL, and a mobile phase flow of 0.6 mL/min. An isocratic elution using 85% 10 mM NH_4HCO_3 and 15% MeOH as mobile phase (total run time: 15 min) was optimized and employed to elute the substrate (2-methylthiazoline; **S1**) and its transformation products in all experiments (elution is shown in Figure S5.1). For the experiment with very high substrate concentration (up to 10'000 µM, described in Section S4.3 2) and shown in Fig. 3b), a column clean-up and reconditioning step was used after all compounds eluted (i.e., after 15 min) to ensure low S-background for the next injection. This clean-up and reconditioning step included a MeOH gradient to 80% (from 15 to 15.1 min) followed by 80% MeOH for 1.9 min (cleaning) and then a gradient of MeOH back to 15% (from 17 to 17.1 min) followed by 15% MeOH for 3.9 min (reconditioning before next injection).

With isocratic elution of the compounds, the sensitivity of the ICP-MS/MS detection was stable over the chromatographic separation, as shown by signals of Sc, Rh, Lu (internal standards added post-column; Figure S5.2). This enables the quantification of the products of the reaction between 2-methylthiazoline (**S1**) and ozone, for which no analytical standards are commercially available, using calibration curves made with **S1** and *N*-acetyltaurine (**P1**) (compound independent calibration; Table S5.11). Thus, **S1** (retention time, RT=11.4 min) and **P1** (RT = 1.8 min) were quantified by compound-specific calibration curves. The products noted **I2** (thiosulfinate, RT = 2.9 min), **impurity of S1** (RT=4.1 min), and **I3** (RT=8.9 min) were quantified using the calibration curve of **P1** (Table S5.11). Finally, product **I1** (disulfide, RT=9.9 min) was quantified using the calibration curve of **S1** (Table S5.11). Note that the **impurity of S1** (RT=4.2 min) did not react with ozone and did not co-elute with any transformation product of the reaction between 2-methylthiazoline (**S1**) and ozone. Note also that, besides the **impurity of S1**, 2.1±0.2% of **I2** (RT= 2.9 min) and 0.9±0.4% of **I1** (RT= 9.8 min) were detected in the **S1** substrate (Figure S5.1a, quantified from the duplicate analysis of **S1** standards at 5 different concentrations, i.e., 5, 10, 20, 50 and 100 µM, so n=10). Peak integration and quantification were done using Agilent Masshunter software (version 4.6).

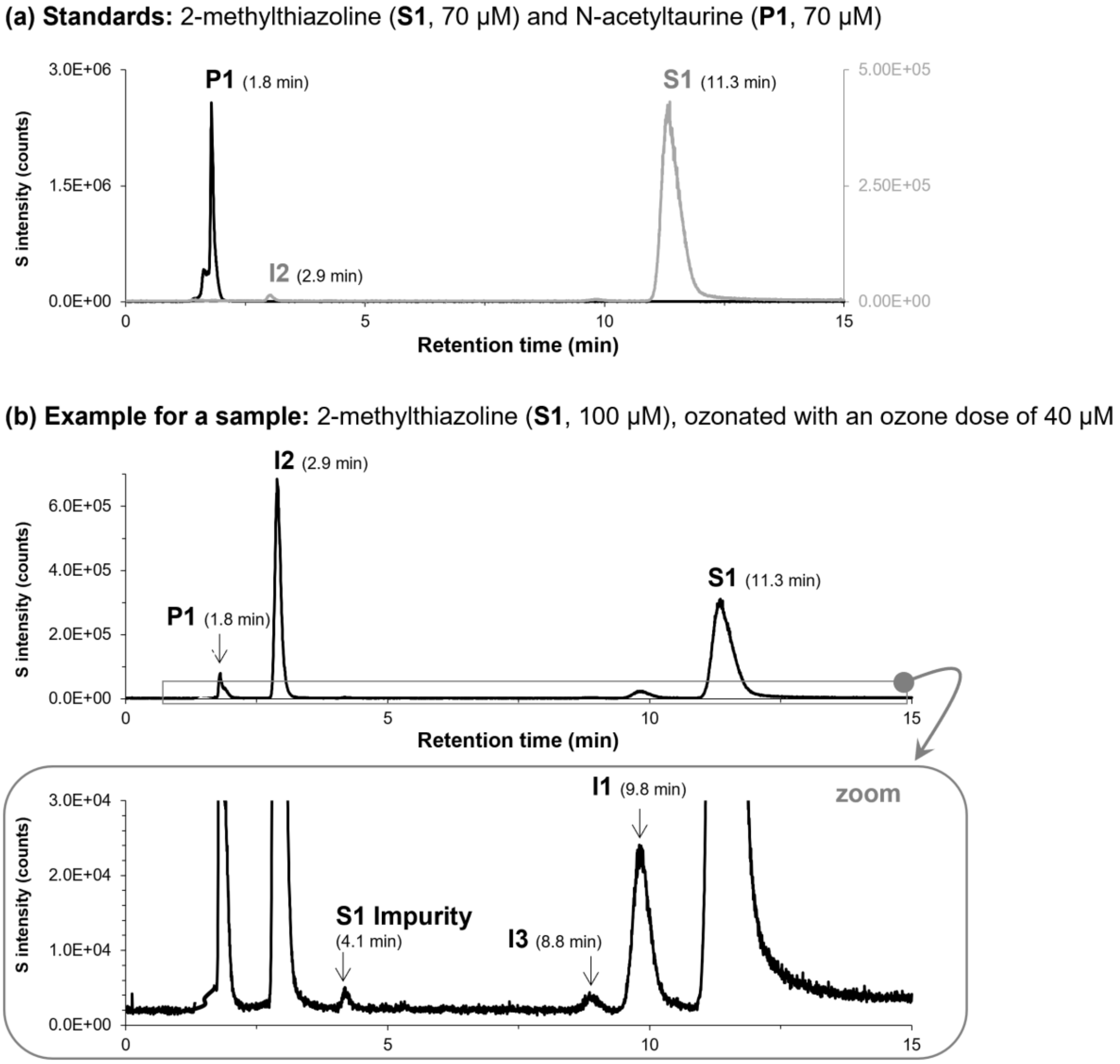

Figure S5.1 – LC-ICP-MS/MS sulfur-chromatograms of (a) the commercially available standards 2-methylthiazoline (**S1**, black) and *N*-acetyltaurine (**P1**, gray) at a concentration of 70 µM and (b) the chromatogram of the ozonated sample of **S1** at a molar [ozone]:[2-methylthiazoline] ratio of 0.4 (Figure 3a, main manuscript; experimental conditions: [2-methylthiazoline] = ~0.1 mM; [*tert*-butanol] = 10 mM; [phosphate buffer (pH 7)] = 5 mM; ozone doses = 0.04 mM). The lower chromatogram shows an enlargement of the upper chromatogram. Traces of **I2** (RT= 2.9 min) and **I1** were detected in **S1** representing 2.1±0.2% and 0.9±0.4% of **S1** concentration, respectively. The **impurity of S1** eluting at 4.1 min did not react with ozone and did not co-elute with any transformation product of the reaction between 2-methylthiazoline (**S1**) and ozone.

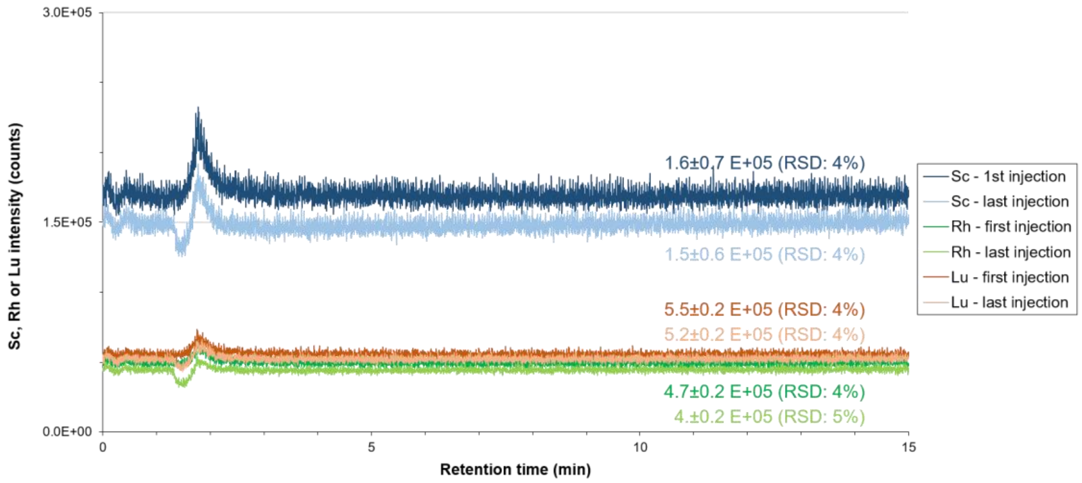

Figure S5.2 – Signal stability for the internal standard elements (scandium (Sc), rhodium (Rh), and lutetium (Lu)) used during the LC-ICP-MS/MS analysis of sulfur-containing substrate and transformation products, demonstrating stability of the ICP-MS/MS detection during the used isocratic chromatographic separation and across an analysis batch. The shown internal standard signals were from the batch in which we analyzed the ozonated substrate (2-methylthiazoline) as a function of the molar specific ozone dose [data shown in Figure 3a, main manuscript]. The 1st injection was a blank ([*tert*-butanol] = 10 mM; [phosphate buffer (pH 7)] = 5 mM) and the last injection was the last sample (i.e., substrate ozonated with a molar [ozone]:[2-methylthiazoline] ratio of 0.4)

Limit of quantification (LOQs,) were calculated as recommended by IUPAC as shown in the equation below,[22] and are provided in Table S5.11.

$$LOQ = \frac{10 \times SD}{slope}$$

with LOQ being quantification limit in µM sulfur; SD is the standard deviation of the background signal of a blank measurement in reconstituted sample matrix (([tert-butanol] = 10 mM; [phosphate buffer (pH 7)] = 5 mM) in counts; slope is the calibration slope in peak height (counts)/µM (sulfur) obtained with standards of **S1** (for LOQs of **S1** and **I1)**, or with standards of **P1** (for LOQs of **P1**, **I2**, **I3**, and **impurity of S1**).

Table S5.11 – Sulfur-containing peaks detected by LC-ICP-MS/MS and the corresponding retention times, method for quantification and measuring ranges as well as limit of quantifications (LOQs; in µM sulfur) for experiments and subsequent analysis shown in Figures 3a and 3b (main manuscript). The LOQs were determined based on the analysis of 6 blanks for the experiments shown in Fig. 3a and S10.4, and 3 blanks for the experiment shown in Fig. 3b, respectively. The higher LOQs for the experiment in Fig. 3b resulted from the lower sensitivity of the S-detection by ICP-MS/MS. The S-background in the injected blanks were similar for all experiments. The lower sensitivity of S-detection in the experiment shown in Figure 3b resulted from the higher percentage of oxygen gas used (20% of total carrier gas flow versus 10% for the other experiments). This higher oxygen gas percentage was required for the use of 80% MeOH in the gradient for the LC clean-up step, as described above.

			**Experiment Fig. 3a and S10.4**		**Experiment Fig. 3b**	
**S peaks**	**RT / min**	**Quantification method**	**Measuring range / µM**	**LOQ / µM (n=6 blanks)**	**Measuring range / µM**	**LOQ / µM (n=2 blanks)**
**S1** (2-methylthiazoline)	11.3	Compound-specific calibration curves	10-100	0.34±0.06	10-10'000	2.6±0.5
**P1** (*N*-acetyltaurine)	1.8	Compound-specific calibration curves	10-100	0.17±0.06	10-100	1.1±0.3
**I1**	9.8	Compound independent (S1) calibration	10-100	0.37±0.08	10-10'000	2.6±0.5
**I2**	2.9	Compound independent (P1) calibration	10-100	0.08±0.01	10-100	1.0±0.7
**I3**	8.8	Compound independent (P1) calibration	10-100	0.077±0.007	10-100	0.52±0.05
**S1 Impurity**	4.1	Compound independent (P1) calibration	10-100	Not detected	10-100	0.8±0.3

S5.6 NMR spectroscopy

^{1}H, ^{13}C and ^{15}N NMR data were recorded on a Bruker AV-III 400 spectrometer (Bruker BioSpin AG, Switzerland) at 400.2, 100.6 and 40.5 MHz, respectively. The 1D ^{1}H and ^{13}C NMR spectra, as well as the 2D correlated ^{1}H-^{13}C HSQC, ^{1}H-^{13}C HMBC, ^{1}H-^{15}N HMBC and ^{1}H-^{1}H DQF-COSY NMR experiments were performed at 298 K using the Bruker standard pulse programs and parameter sets on a 5 mm CryoProbe™ Prodigy probe. The ^{1}H and ^{13}C NMR chemical shifts are calibrated with respect to the resonances of the DMSO solvent at 2.49 and 39.5 ppm, respectively and the ^{15}N NMR chemical shifts are reported on the NH_3 chemical shift scale. Coupling constants *J* are reported in Hz and for ^{1}H NMR data coupling patterns are described as *s* = singlet, *d* = doublet, *t* = triplet, *q* = quartet, *m* = multiplet, *br* = broad and for ^{13}C NMR data *s* = quaternary carbon, *d* = CH, *t* = CH_2, and *q* = CH_3.

S6 Ozonation of thiazole in excess of t

To investigate the first step of the ozone attack on thiazole, experiments were conducted with molar [thiazole]:[ozone] ratios from 2 to 100. The yields of the anionic products were determined with IC-CD (Section S5.2). The results are displayed in Figure S6.1.

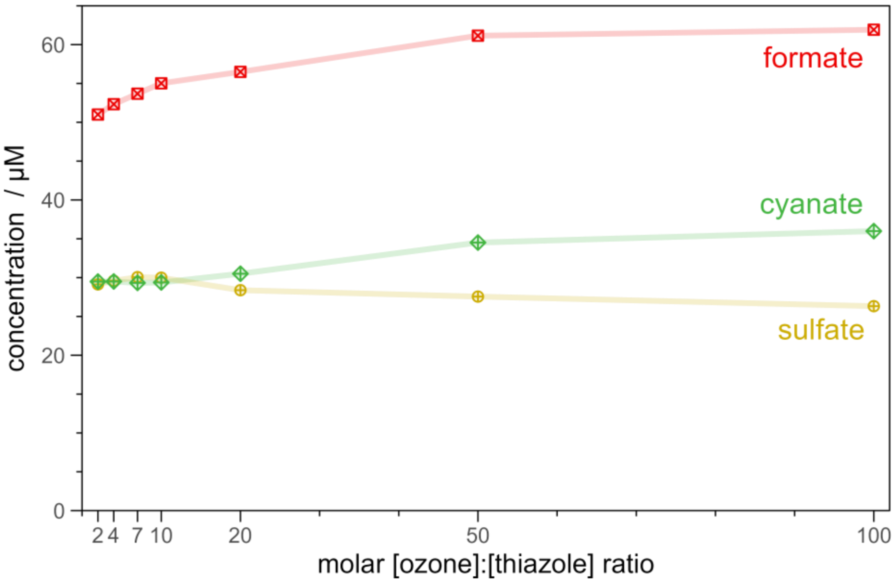

Figure S6.1 – Ozonation of thiazole in presence of the $^{\bullet}OH$ scavenger *tert*-butanol with varying molar [thiazole]:[ozone] ratios: measured concentrations of formate, cyanate and sulfate after ozonation. Experimental conditions: [thiazole] = ~0.1 to 10 mM; [*tert*-butanol] = 10 mM; [phosphate buffer (pH 7)] = 5 mM; ozone dose = 0.1 mM. Results from single experiments are shown.

S7 Reaction of oxazole-4-carboxamide with ozone

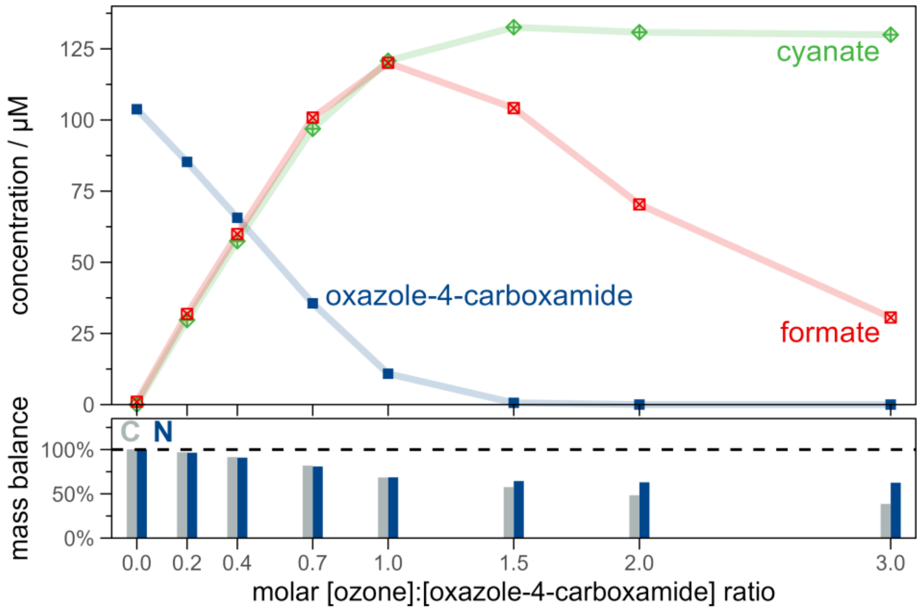

Figure S7.1 – Abatement of oxazole-4-carboxamide during ozonation in presence of the •OH scavenger *tert*-butanol and the formation of the detected transformation products formate and cyanate. The C and N mass balances for the transformation is also shown (bottom). Experimental conditions: [azole] = ~0.1 mM; [*tert*-butanol] = 10 mM; [phosphate buffer (pH 7)] = 5 mM; ozone doses = 0–0.5 mM. Results from a single experiment are shown. Ozone doses were not measured separately (see Section 2.2, main manuscript).

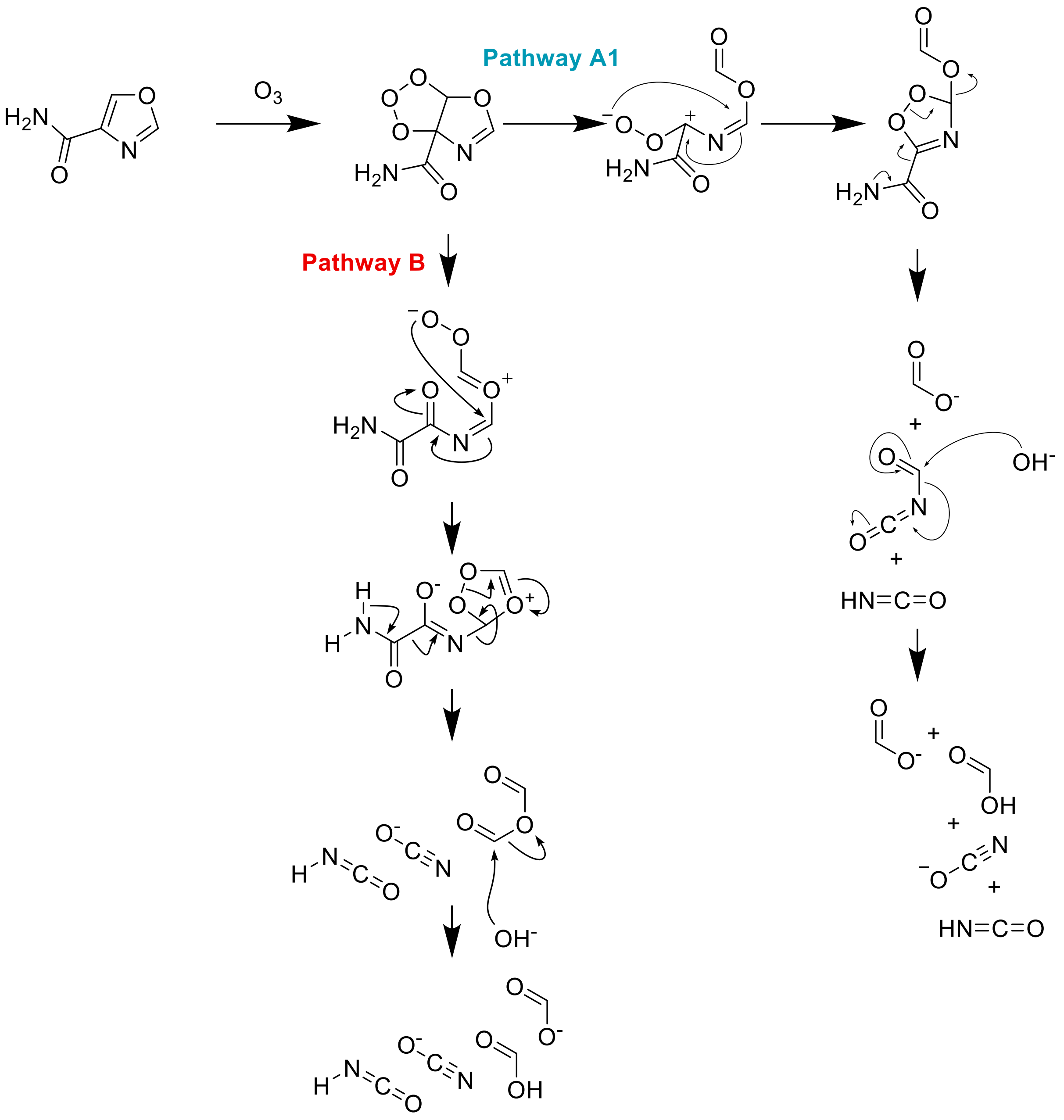

Scheme S7.1 – Proposed reaction mechanisms for the reactions of oxazole-4-carboxamide with ozone based on the identified reaction products, proceeding through pathway A1 or B (Scheme 1, main manuscript). Both pathways lead to the same final reaction products formate and cyanate.

S8 Reaction of 2-methylthiazole with ozone: detection of thioacetate, thioformate with LC-HRMS and an intermediate with IC-CD and IC-HRMS

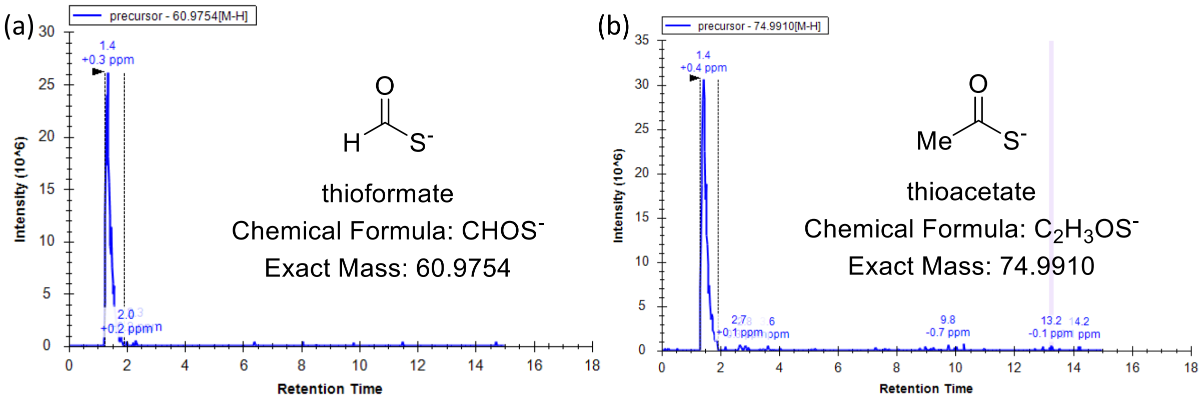

Figure S8.1 – Ozonation of 2-methylthiazole in in presence of the •OH scavenger *tert*-butanol: LC-MS[1] chromatograms of (a) thioformate (M–H$^+$: 60.9754) and (b) thioacetate (M–H$^+$: 74.9910). Experimental conditions: [2-methylthiazole] = ~0.1 mM; [*tert*-butanol] = 10 mM; [phosphate buffer (pH 7)] = 5 mM; ozone doses = 0.2 mM.

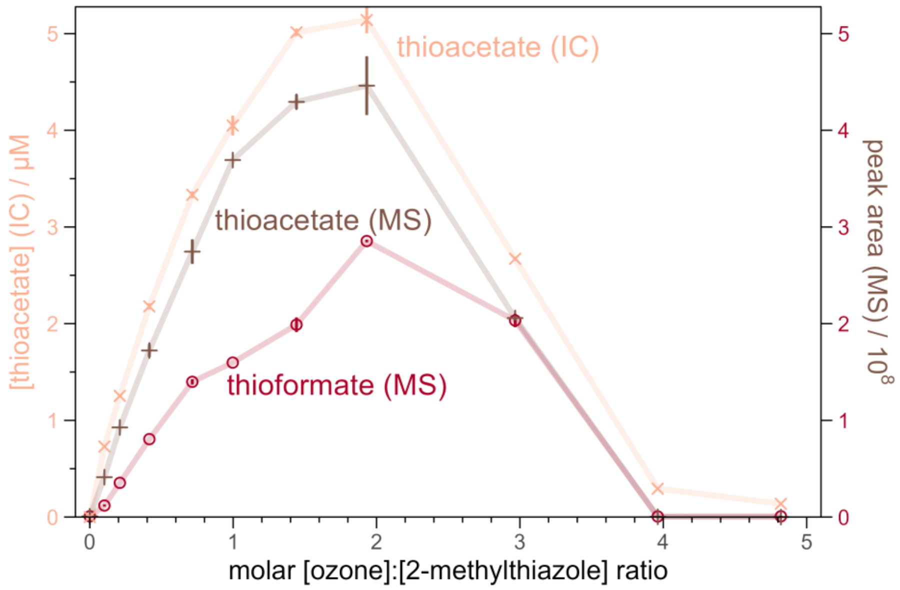

Figure S8.2 – Ozonation of 2-methylthiazole in presence of the •OH scavenger *tert*-butanol: measured concentration of thioacetate with IC-CD (orange, primary y-axis) and observed peak areas for thioacetate (brown, secondary y-axis) and thioformate (red, secondary axis) as a function of the molar [ozone]:[2-methylthiazole] ratio. Experimental conditions: [2-methylthiazole] = ~0.1 mM; [*tert*-butanol] = 10 mM; [phosphate buffer (pH 7)] = 5 mM; ozone doses = 0–0.5 mM. Results are shown as average from duplicate experiments, error bars represent the upper and the lower of the measured values (if not visible, the range falls within the symbol size).

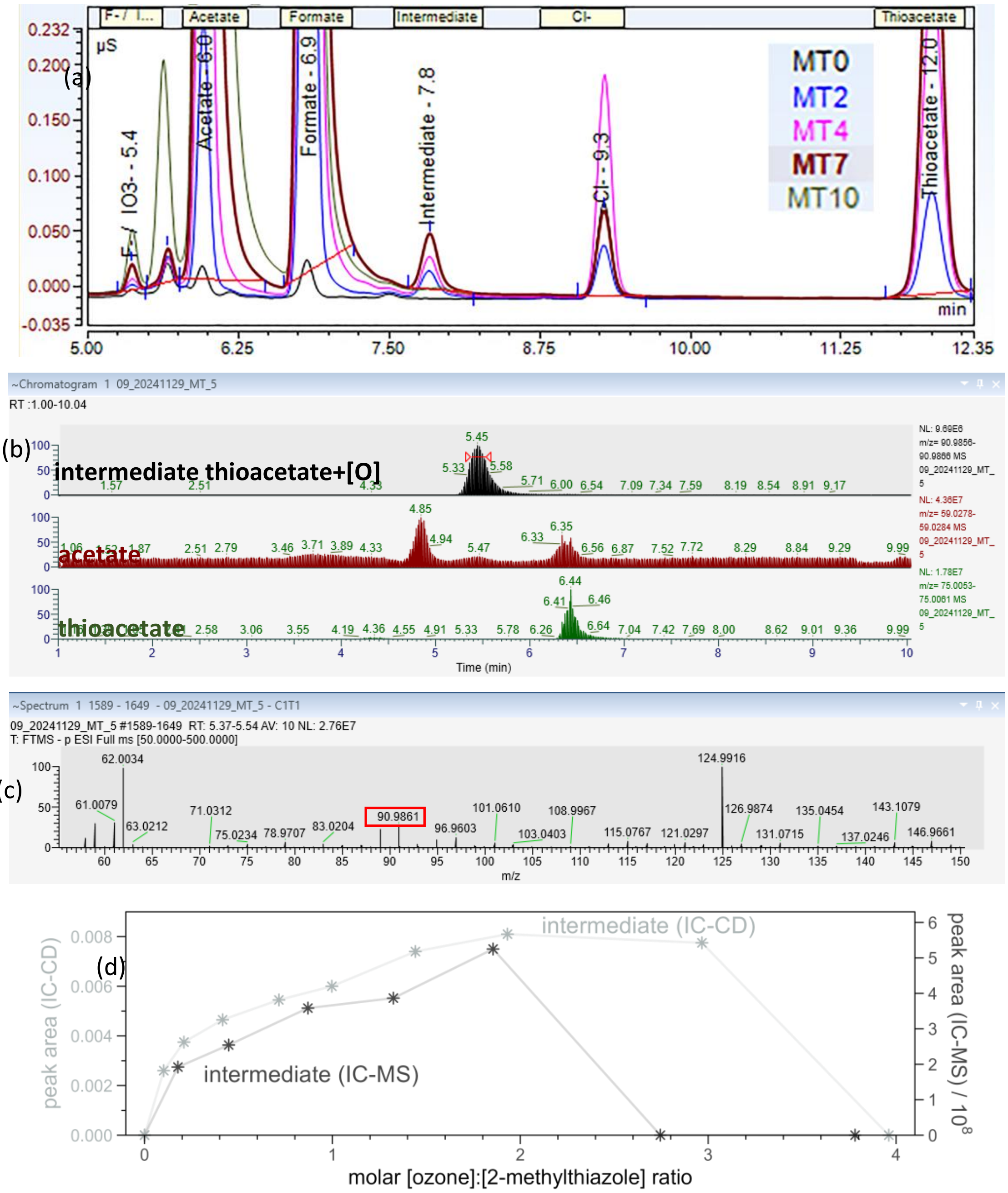

Figure S8.3 – Ozonation of 2-methylthiazoline (MT) in presence of the $^{\bullet}$OH scavenger *tert*-butanol: detection of an anionic intermediate with a retention time of 7.8 min (IC-CD) or 5.45 min (IC-HRMS). (a) IC-CD chromatograms of the reaction solutions with ozone doses of 0 mM (MT0), 0.02 mM (MT2), 0.07 mM (MT4), 0.18 mM (MT7) and 0.45 mM (MT10). Shown is the section with RT 5–12 min, with the peaks of the intermediate, the products acetate, formate, and thioacetate, as well as the trace chloride. (b) IC-HRMS chromatogram of the reaction solution with ozone dose of 0.19 mM, filtered by *m/z* = 90.9861 (thioacetate+[O], black, RT = 5.5 min), *m/z* = 59.0281 (acetate, red, RT = 4.8 min, the second peak at 6.4 min is likely due to in-source fragmentation of thioacetate), and *m/z* = 75.0058 (thioacetate, green, RT = 6.5 min). The retention time of thioacetate+[O] is in between the retention times of acetate and thioacetate, as observed for IC-CD. (c) MS^1 spectrum of the peak at a retention time of 5.5 min. (d) Detected area of the intermediate peak with IC-CD (RT = 7.8 min) and IC-MS (RT = 5.5 min), as a function of the molar [ozone]:[2-methylthiazole] ratio. Note: the two data sets were acquired in two separate experiments. Experimental conditions: [2-methylthiazole] = ~0.1 mM; [*tert*-butanol] = 10 mM; [phosphate buffer (pH 7)] = 5 mM; ozone doses = 0–0.5 mM. Results are shown from single experiments.

S9 Reaction of thioacetate with ozone

Reaction kinetics: The apparent second-order rate constant of the reaction of ozone with thioacetate was determined by competition kinetics with cinnamic acid as a competitor as described in Section S3.3, with a determined $k_{O_3+thioacetate}$ = 1.54×10^6 M^{-1}s^{-1}. No second-order rate constants have yet been reported for similar compounds with ozone. Other sulfurous compounds have second-order rate constants in a similar range, e.g. thioethers (10^5 – 10^6 M^{-1}s^{-1})[23–25] or sulfinates (10^6 M^{-1}s^{-1}).[26]

Reactive oxygen species: For the reaction of thioacetate with ozone, only singlet oxygen ($^1O_2$) was measured, because it can be assumed that $^{\bullet}OH$ and H_2O_2 are not products of the reaction of thioacetate with ozone. This is, because in the reaction of 2-methylthiazole, only trace amounts of $^{\bullet}OH$ and 2% yield of H_2O_2 were detected and thioacetate is a major intermediate in the oxidation of 2-methylthiazole.

In order to obtain more detailed information about the different steps of the reaction, the yields of $^1O_2$ were determined for a large excess of substrate, as well as for a large excess of ozone. Since two equivalents of ozone are required for the complete oxidation of thioacetate (see below), the measured $^1O_2$ yield under conditions with a molar [thioacetate]:[ozone] ratio of ≤ 0.5 is representative for the sum of both reaction steps. With increasing molar [thioacetate]:[ozone] ratios, the first of the two reaction steps is increasingly dominant in the $^1O_2$ yield. Because the second-order rate constant of the second reaction step is unknown, the exact $^1O_2$ yields of the first and second reaction steps cannot be individually determined. All experiments were performed in triplicates. The results are shown in Table S9.1 and Figure S9.1.

Table S9.1 – $^1O_2$ yield for the reaction of ozone with thioacetate at different molar [ozone]:[thioacetate] ratios. Experimental conditions: [*tert*-butanol] = 100 mM; [phosphate buffer (pH 7)] = 10 mM. The two different $^1O_2$ yields shown are relative to the substrate concentration and relative to ozone concentration, respectively.

[thioacetate] / µM	[O_3] / µM	[thioacetate]/[O_3]	[O_3]/[thioacetate]	$^1O_2$ yield / [thioacetate]	$^1O_2$ yield / [O_3]
10	45	0.22	4.45	122%	
20	46	0.44	2.29	92%	
25	47	0.54	1.87	71%	38%
30	48	0.63	1.59	60%	38%
50	48	1.05	0.96	29%	30%
500	398	1.26	0.80		23%
1000	377	2.65	0.38		15%
2000	354	5.65	0.18		17%
4000	333	12.02	0.08		14%
7000	295	23.77	0.04		15%

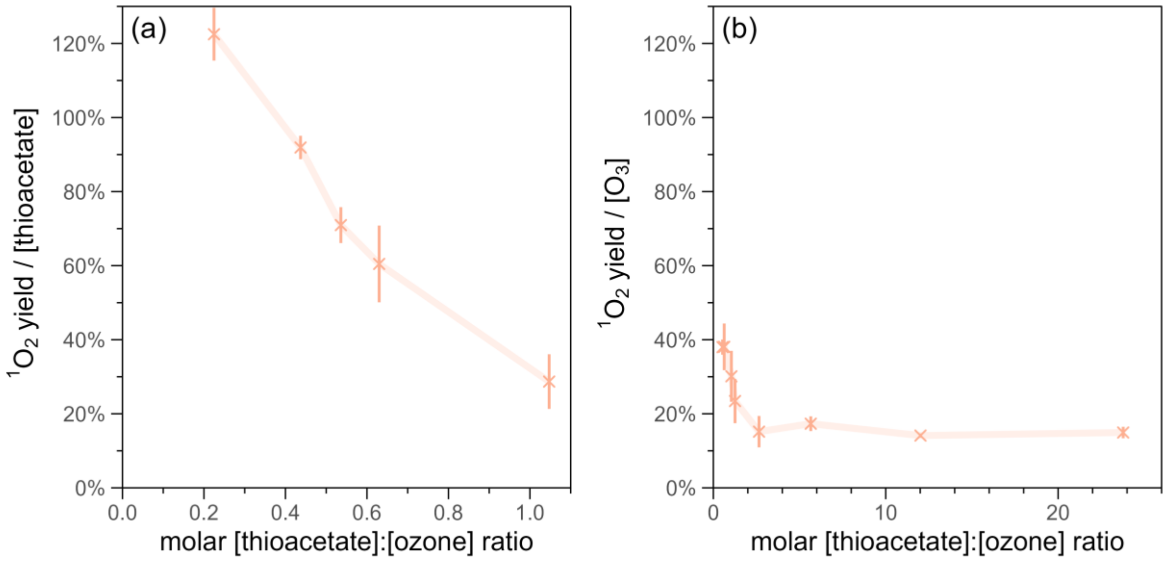

Figure S9.1 – $^1O_2$ yields for the reaction of ozone with thioacetate in presence of the $^{\bullet}OH$ scavenger *tert*-butanol with varying molar [ozone]:[thioacetate] ratios. (a) $^1O_2$ yield relative to the concentration of thioacetate as a function of the molar [ozone]:[thioacetate] ratio for ratios ≤ 1, because with excess ozone, thioacetate is the limiting factor for the $^1O_2$ yield. (b) $^1O_2$ yield relative to the ozone dose as a function of the molar [ozone]:[thioacetate] ratio for ratios ≥ 0.5, because with excess substrate, the ozone dose is the limiting factor for the $^1O_2$ yield. Experimental conditions: [*tert*-butanol] = 100 mM; [phosphate buffer (pH 7)] = 10 mM; [thioacetate] and ozone doses are described in Table 9.1. Results are shown as average from triplicate experiments, error bars represent the standard deviations (if not visible, the error falls within the symbol size).

Figure S9.1a shows higher $^1O_2$ yields (relative to [thioacetate]) with increasing molar excess of ozone. At a molar [thioacetate]:[ozone] ratio of 1, the $^1O_2$ yield is only 29% and with increasing excess of thioacetate (Figure S9.1b), the $^1O_2$ yield (relative to [ozone]) converges to 15%. A $^1O_2$ yield following this trajectory could be the result of a multi-step process, if the initial reaction with ozone has a lower $^1O_2$ yield, and is followed by one or several $^1O_2$ producing reactions.

Oxidation products and intermediates: The abatement of thioacetate during ozonation and the formation of transformation products is shown in Figure S9.2. Thioacetate is fully abated with two equivalents of ozone. Based on the slopes of the curves at low molar [ozone]:[thioacetate] ratios, yields of 100 % and 77 % were determined for the two products acetate and sulfate, respectively. Despite the initially lower yield determined for sulfate, concentrations similar to acetate are detected at higher ozone doses. This can be attributed to the presence of sulfur-containing intermediates after the initial cleavage of the C-S-bond.

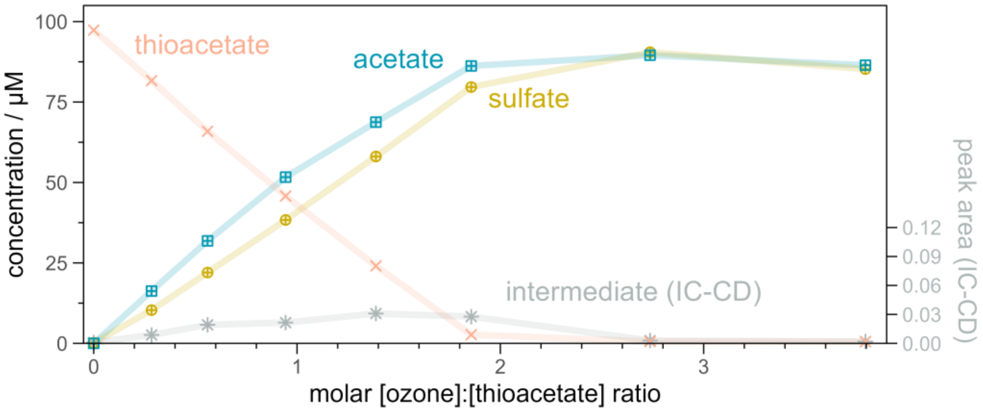

Figure S9.2 – Abatement of thioacetate during ozonation in presence of the •OH scavenger *tert*-butanol and the formation of the transformation products acetate and sulfate, determined by IC-CD. Included is also the peak area of the intermediate at a retention time of 7.8 min (grey, secondary axis, see Figure S9.4a for more details). Experimental conditions: [thioacetate] = ~0.1 mM; [*tert*-butanol] = 10 mM; [phosphate buffer (pH 7)] = 5 mM; ozone doses = 0–0.4 mM. Results from a single experiment are shown.

One possible intermediate product on the reaction path to sulfate could be sulfite. In reaction solutions with moderate ozone doses, a shoulder appears at a slightly increased retention time adjacent to the sulfate peak, which vanishes at higher ozone doses (Figure S9.3). To verify this experimentally, a 5 µM sulfite standard was measured (Figure S9.3, gray), corresponding to a 25% sulfite yield in the reaction (samples are 1:5 diluted, as described in Section S5.2). However, as the peak shoulder in the reaction solution is visually much smaller, the sulfite formed cannot fully account for the difference between acetate and sulfate yields, suggesting it is not the only sulfur-containing intermediate. Given the stoichiometry indicates a two-step reaction, it is plausible that sulfite formation represents just one of several pathways ultimately leading to sulfate.

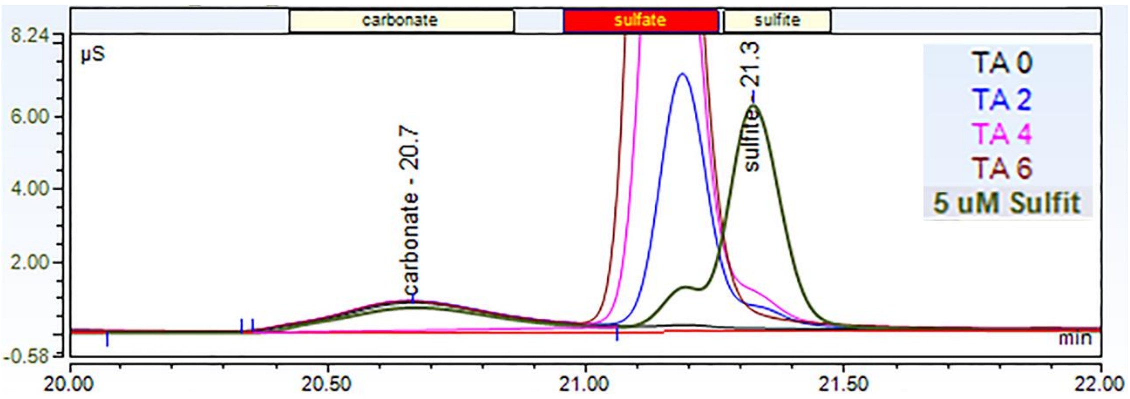

Figure S9.3 – Ozonation of thioacetate (TA) in presence of the •OH scavenger *tert*-butanol and detection of intermediates: IC-CD chromatograms of the reaction solutions, with ozone doses of 0 mM (TA0), 0.06 mM (TA2), 0.14 mM (TA4) and 2.7 mM (TA6). Quantitative results are shown in Figure S9.2. Shown is the section with RT 20–22 min, showing the sulfate peak at 21.2 min with a slight shoulder at higher RT. The chromatogram of 5 µM sulfite is included (grey, RT = 21.3 min). Experimental conditions: [thioacetate] = ~0.1 mM; [*tert*-butanol] = 10 mM; [phosphate buffer (pH 7)] = 5 mM; ozone doses = 0–0.27 mM.

In the IC-CD measurements, a peak attributed to an unidentified chemical species emerges at a retention time of 7.8 minutes (Figure S9.4a). This peak appears at low ozone doses, then decreases at higher ozone doses (see the evolution of the peak area in Figure S9.2, grey, secondary axis, IC chromatogram in Figure S9.4a). The same pattern was already observed during ozonation of 2-methylthiazole (Section 3.2.2 of the main manuscript, and Figure S8.3). In IC-HRMS-measurements, a peak with the same formation pattern (Figure S9.4d) and a retention time also between the retention times of acetate and thioacetate has been observed (Figure S9.4b). The IC-CD and the IC-HRMS measurements were performed on different IC instruments. Given the congruent formation patterns and similar relative retention times, it is reasonable to infer that these peaks represent the same compound. The peak has an *m/z* of 90.9861 (Figure S9.4c), corresponding to an oxygen-atom transfer to thioacetate. This indicates acetylsulfenic acid as a possible intermediate, since sulfur is the only plausible reaction site for ozone. (Scheme S9.1). MS^2 measurements were not triggered under these conditions.

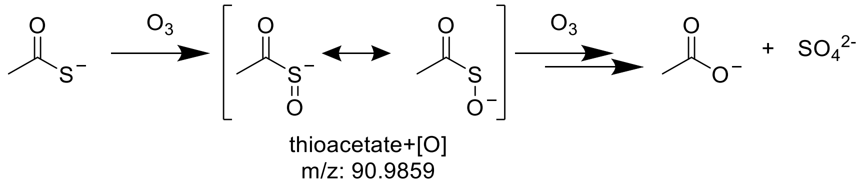

Scheme S9.1 – Possible formation of the observed intermediate thioacetate+[O] as intermediate of the ozonation of thioacetate to acetate and sulfate.

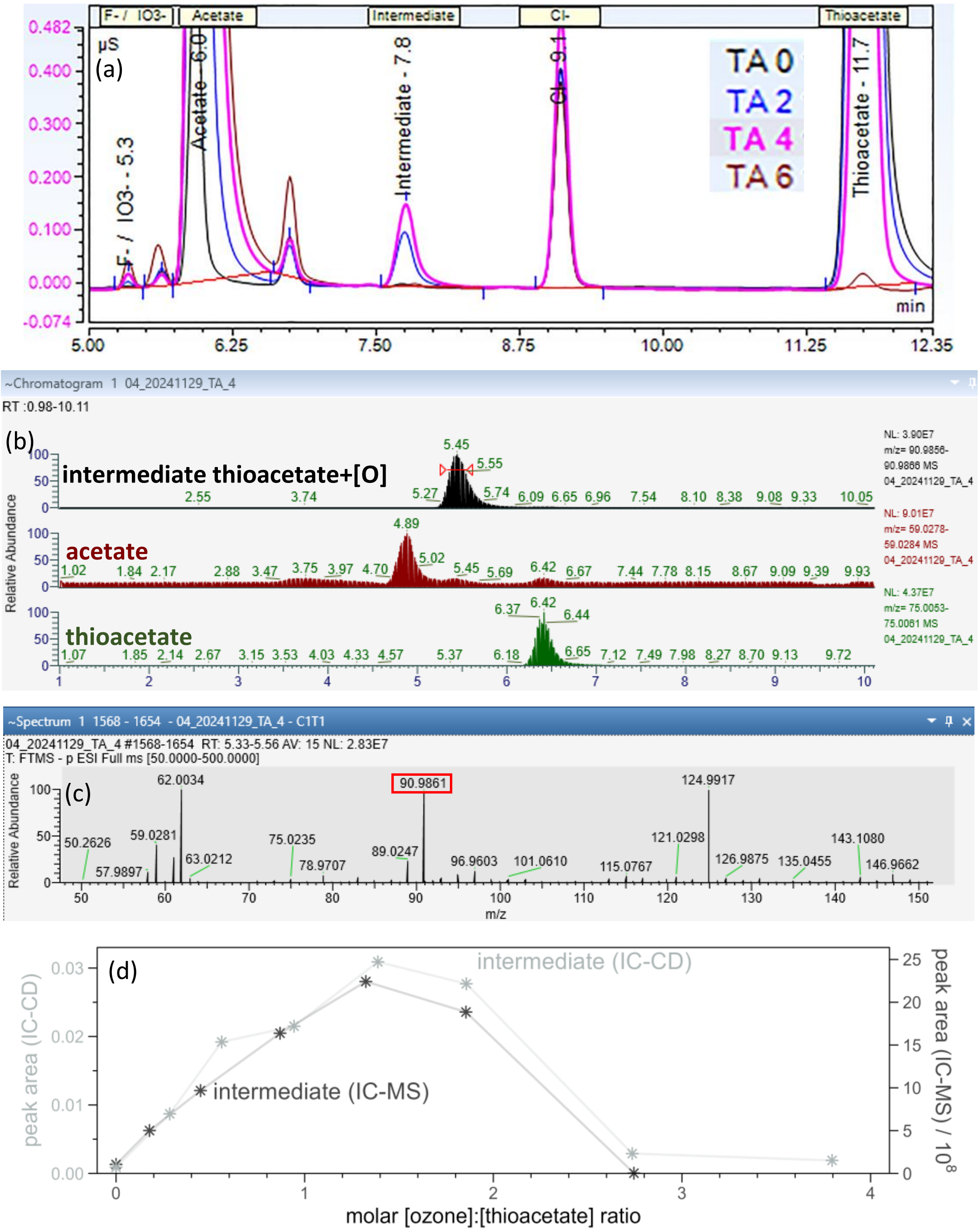

Figure S9.4 – Ozonation of thioacetate (TA) in presence of the •OH scavenger *tert*-butanol: detection of an intermediate with a retention time of 7.8 min (IC-CD) or 5.45 min (IC-MS): (a) IC-CD chromatogram section with RT 5–12 min of the reaction solutions, with ozone doses of 0 mM (TA0), 0.06 mM (TA2), 0.14 mM (TA4) and 2.7 mM (TA6), showing the peak of an intermediate at RT = 7.8 min, only visible in the chromatograms of TA2 and TA4. (b) IC-HRMS chromatogram of the reaction solution with ozone dose of 0.19 mM, filtered by *m/z* = 90.9861 (thioacetate+[O], black, RT = 5.5 min), *m/z* = 59.0281 (acetate, red, RT = 4.8 min, the second peak at 6.4 min is likely due to in-source fragmentation of thioacetate), and *m/z* = 75.0058 (thioacetate, green, RT = 6.5 min). The retention time of this intermediate is between the retention times of acetate and thioacetate, like in the IC-CD measurement. (c) MS^1 spectrum of the peak at the retention time of 5.5 min. (d) Detected area of the product peak with IC-CD (RT = 7.8 min) and IC-MS (RT = 5.5 min), as a function of the molar [ozone]:[2-methylthiazole] ratio. Note: the two data sets were acquired in two separate experiments. Experimental conditions: [thioacetate] = ~0.1 mM; [*tert*-butanol] = 10 mM; [phosphate buffer (pH 7)] = 5 mM; ozone doses = 0–0.27 mM.

S10 Additional experiments on the reaction of 2-methylthiazoline with ozone

S10.1 Measurement of singlet oxygen ($^1O_2$) yield for the ozonation of 2-methylthiazoline at different molar ratios

Given that the ozonation of 2-methylthiazoline involves multiple steps (see Scheme 2a, main manuscript), we conducted measurements of the $^1O_2$ yields for different molar [2-methylthiazoline]:[ozone] ratios. This approach aims to elucidate the $^1O_2$ yield associated with each reaction step (results shown in Figure S10.1). For the chosen experimental conditions, it is expected that ozone is fully consumed by several ozonation steps (Scheme 2a, main manuscript). Therefore, ozone is the limiting reagent and the $^1O_2$ yield is plotted relative to the ozone dose (unlike in Figure S9.1, where the limiting reagent depends on the molar [ozone]:[thioacetate] ratio).

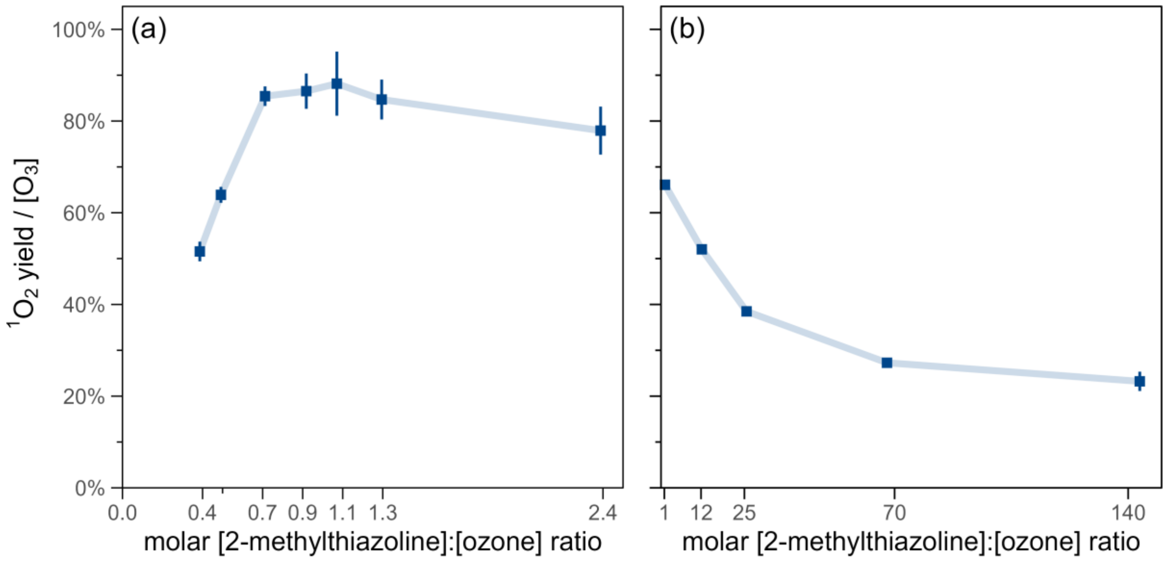

Figure S10.1 – $^1O_2$ yield normalized to the ozone dose from the reaction of 2-methylthiazoline with ozone as a function of the molar [2-methylthiazoline]:[ozone] ratio with (a) experiments with low molar [2-methylthiazoline]:[ozone] ratios; (b) experiments in excess of 2-methylthiazoline. Results are shown as averages of 3-10 replicates per data point. The error bars represent standard deviations (if not visible, the range falls within the symbol size). Experimental conditions: (a) [2-methylthiazoline] = ~0.125–1 mM; ozone doses = 0.3–1 mM; [*tert*-butanol] = 100 mM; [phosphate buffer (pH 7)] = 10 mM. (b) [2-methylthiazoline] = ~0.1–10 mM; [*tert*-butanol] = 100 mM; [phosphate buffer (pH 7)] = 10 mM; ozone doses = 0.07–0.1 mM.

According to the results shown in Section 3.2.4 (main manuscript), at a molar [2-methylthiazoline]:[ozone] ratio <5, the mainly formed reaction product is the thiosulfinate ester **I2** (Figures 3a and 3b, upper right, main manuscript). Thus, in the results displayed in Figure S10.1a, it can be assumed that the measured $^1O_2$ yield represents the $^1O_2$ yield of reactions (1) and (2) (Scheme 2a,

main manuscript). A consistent measurement of ~85 % $^1O_2$ at molar [2-methylthiazoline]:[ozone] ratio between 0.7 and 1.3 indicates that both reaction steps produce $^1O_2$. At lower molar [2-methylthiazoline]:[ozone] ratios (e.g. 0.5 and 0.4), further oxidation of the formed intermediate **I2** to **P1** partly consumes ozone. The decreasing $^1O_2$ yield for these small molar [2-methylthiazoline]:[ozone] ratio indicates that these reaction steps at least partly do not produce $^1O_2$.

Experiments with a high excess of substrate (at a lower ozone dose of ~0.01 mM) were performed to isolate the first reaction step. The measured $^1O_2$ yield per consumed ozone decreased continuously from 66% to 26% (Figure S10.1b). This is unexpected, as the former results indicate that also the initial reaction of ozone with 2-methylthiazoline produces $^1O_2$. However, this might be explained by $^1O_2$-scavenging of 2-methylthiazoline itself, due to its nucleophilicity. Reported second-order rate-constants for the reactions of sulfur-compounds with $^1O_2$ are in the range of $k_{^1O_2+\mathrm{sulfurcompounds}} = 10^6$–$10^8$ $M^{-1}s^{-1}$ (e.g. diethylsulfide: 1.8×10^7).[27,28] Thus, at 2-methylthiazoline concentrations of up to 10 mM, apparent $^1O_2$ consumption rate-constants could be in the range of 10^4–10^6 s^{-1}, which is similar to the first-order decay rate of $^1O_2$ in water ($k_{^1O_2} = 2.5\times10^5$ s^{-1}).[29]

S10.2 LC-UV Chromatograms of the ozonation of 2-methylthiazoline

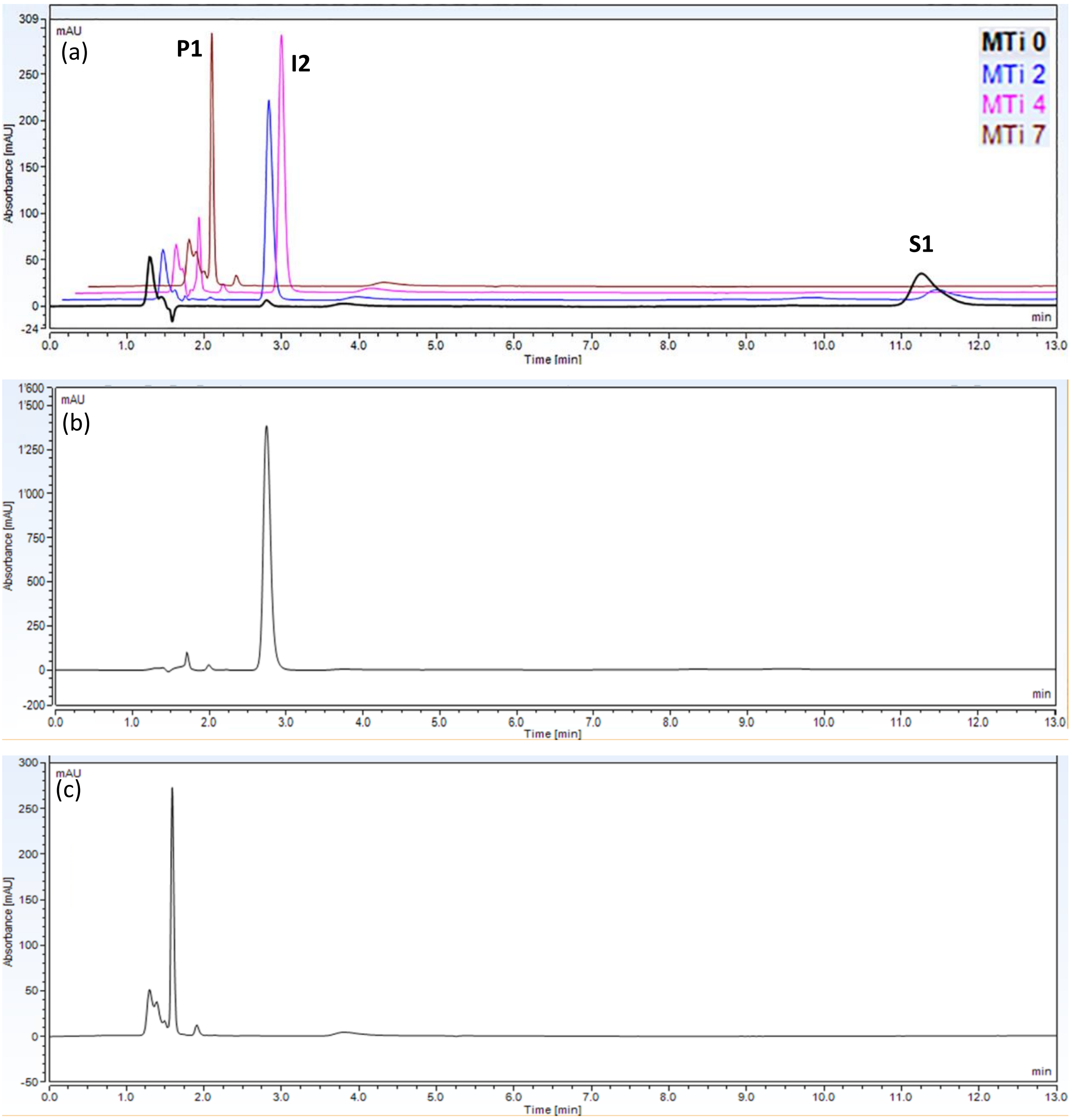

Figure S10.2 – LC-UV chromatograms of the ozonation of 2-methylthiazoline (**S1**). (a) LC-UV chromatograms of the reaction solutions with ozone doses of 0 mM (MTi0), 0.04 mM (MTi2), 0.10 mM (MTi4) and 0.30 mM (MTi7). Experimental conditions: [2-methylthiazoline] = ~0.1 mM; [*tert*-butanol] = 10 mM; [phosphate buffer (pH 7)] = 5 mM; ozone doses = 0–0.3 mM. (b) LC-UV chromatogram of the isolated compound **I2**. (c) LC-UV chromatogram of the isolated compound **P1**.

S10.3 Replicate experiment of the reaction of 2-methylthiazoline with ozone

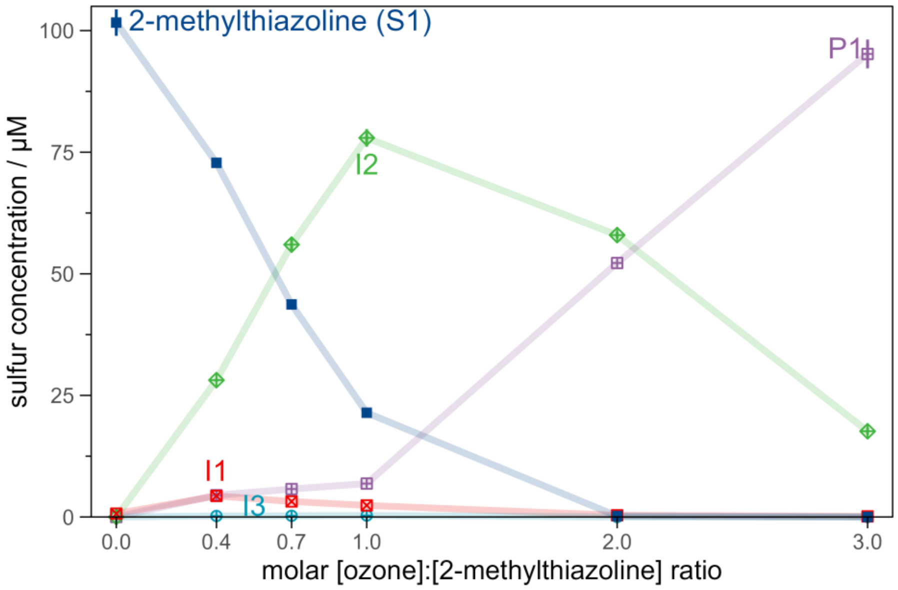

Figure S10.3 – Abatement of 2-methylthiazoline (**S1**) and formation of the intermediates **I1**, **I2** and **I3** and the final product **P1** as a function of the molar specific ozone dose in presence of the •OH scavenger *tert*-butanol quantified by LC-ICP-MS/MS. Experimental conditions: [2-methylthiazoline] = ~0.1 mM; [*tert*-butanol] = 10 mM; [phosphate buffer (pH 7)] = 5 mM; ozone doses = 0–0.3 mM. Results are shown from one experiment. Error bars represent the upper and lower of the measured values of two injections. The results shown here were obtained according to the experiments shown in Figure 3a. Although slightly different ozone doses were used, the overall trends of all quantified products in the two experiments are consistent.

S10.4 Ozonation of 2-methylthiazoline with variation of initial substrate and ozone concentrations

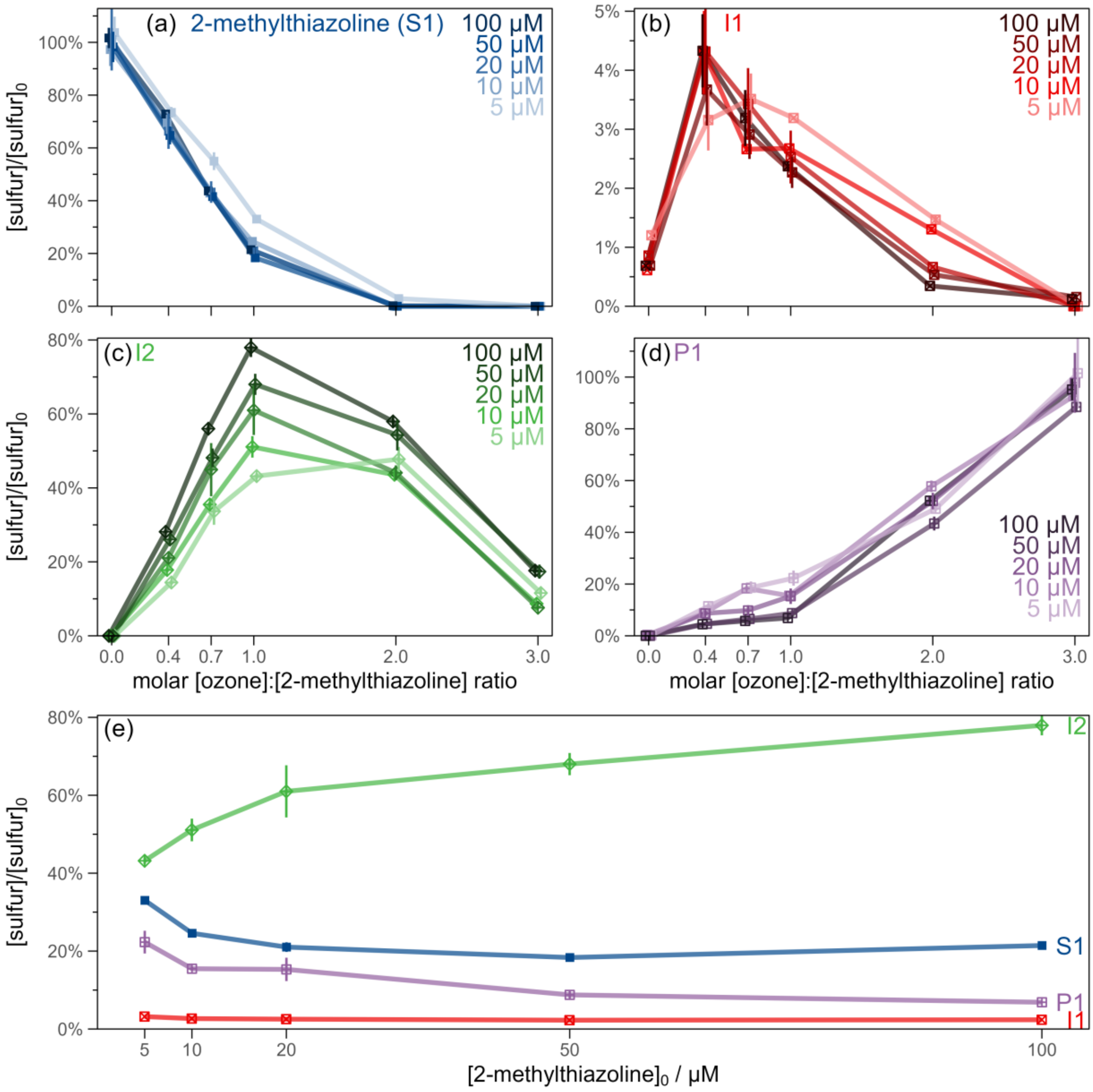

Figure S10.4 – Abatement of 2-methylthiazoline (**S1**) and formation of the intermediates **I1**, **I2** and the final product **P1** at different initial concentrations of 2-methylthiazoline in presence of the •OH scavenger *tert*-butanol, quantified by LC-ICP-MS/MS. Experimental conditions: [2-methylthiazoline] = 5–100 µM; [*tert*-butanol] = 10 mM; [phosphate buffer (pH 7)] = 5 mM; ozone doses = (0 to 3) × [2-methylthiazoline]. Relative concentrations of (a) the substrate **S1**, (b) the intermediates **I1** and (c) **I2** and (d) the product **P1**, as a function of the molar [ozone]:[2-methylthiazoline] ratio. Darkest: highest concentration (100 µM) to lightest: lowest concentration (5 µM). (e) Relative concentrations of **S1** (blue), **I1** (red), **I2** (green) and **P1** (purple) at a molar ozone:[**S1**] molar ratio = 1 as a function of the substrate concentration. Results are shown as average from the LC-ICP-MS/MS analysis in duplicate of each sample from this experiment. Error bars represent the upper and lower of the measured values of two injections.

S11 LC-HRMS/MS-data for product identification of 2-methylthiazoline

The following HRMS/MS data of the four product peaks have been measured from reaction solutions of 2-methylthiazoline with ozone with different molar [2-methylthiazoline]:[ozone] ratios. The exact experimental conditions are provided in the figure captions. The retention times of the products match with the LC-ICP-MS/MS experiments (Figure S5.1). The chemical structure of compounds **I2** has been elucidated by HRMS/MS (Section S11.2) and NMR (Section S12.1). The HRMS/MS data of **P1** (Section S11.4) has been compared to previously reported data of *N*-acetyltaurine (Figure S11.9),[30] and the NMR data (Section 12.2) has been compared to a commercially available standard.

The identification of **I1** relies on the comparison of its molecular formula and MS^2 spectra to **I2** and is described in more detail in Section S11.1.

The chemical structure of **I3** has not been identified, but similarities to the other products detected in the MS data of **I3** are discussed in Section S11.3.

S11.1 LC-HRMS/MS data of I1

The MS^1 spectrum of **I1** provides a *m/z* of 237.0726, corresponding to the molecular formula $C_8H_{16}N_2O_2S_2{+}H^+$ (Figure S11.1), which is equivalent to the molecular formula of **I2**-[O] (Figure S11.3, see below). The isotopic pattern matches that of a S_2-species. One in source-fragment with *m/z* of 118.0322 is detected, corresponding to the molecular formula $C_4H_8NOS{+}H^+$, thus being exactly half of the compound, suggesting a symmetrical molecule. In the MS^1 spectrum of **I2**, this fragment is also observed, but additionally, a fragment at 134.0271, corresponding to 118.0322 plus oxygen is detected. From this observation alone, it can already be proposed that **I1** is a symmetric disulfide.

Comparing the MS^2 spectra of **I1** and **I2** (Figure 11.2 and 11.4, respectively), many fragments can be detected for both compounds. Distinct differences, confirming the structure of I1 as a disulfide, are the observation of fragment 150.0042, and the absence of fragment 134.0271. The fragment with *m/z* of 150.0042 contains a non-oxidized disulfide bond (Figure S11.2), while the fragment with *m/z* of 134.0271 contains an oxidized sulfur. This can be considered sufficient evidence to propose the disulfide structure for compound **I1**, as shown in Figure S11.2.

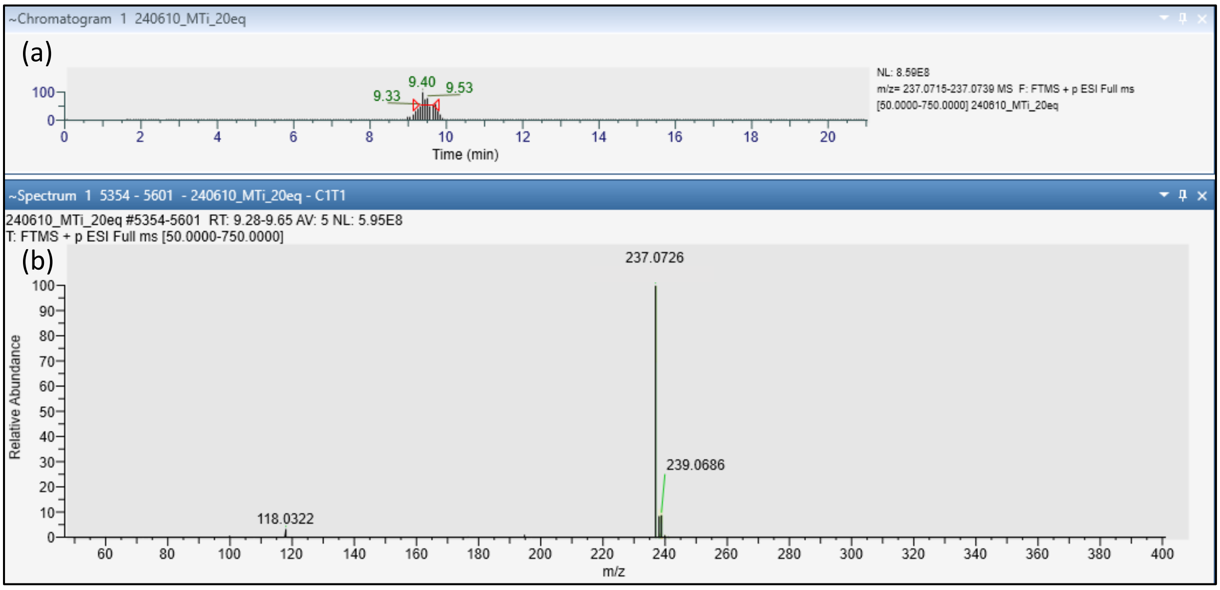

Figure S11.1 – (a) LC-HRMS/MS chromatogram (LC method E, Table S5.5) for an ozonated sample of 2-methylthiazoline in presence of the $^{\bullet}OH$ scavenger *tert*-butanol, filtered by *m/z* = 237.0726 (**I1**+H^+) and (b) MS^1 spectrum of the peak at the retention time of 9.4 min. Experimental conditions: [2-methylthiazoline] = 2 mM; ozone dose = 0.1 mM; [*tert*-butanol] = 10 mM; [phosphate buffer (pH 7)] = 10 mM

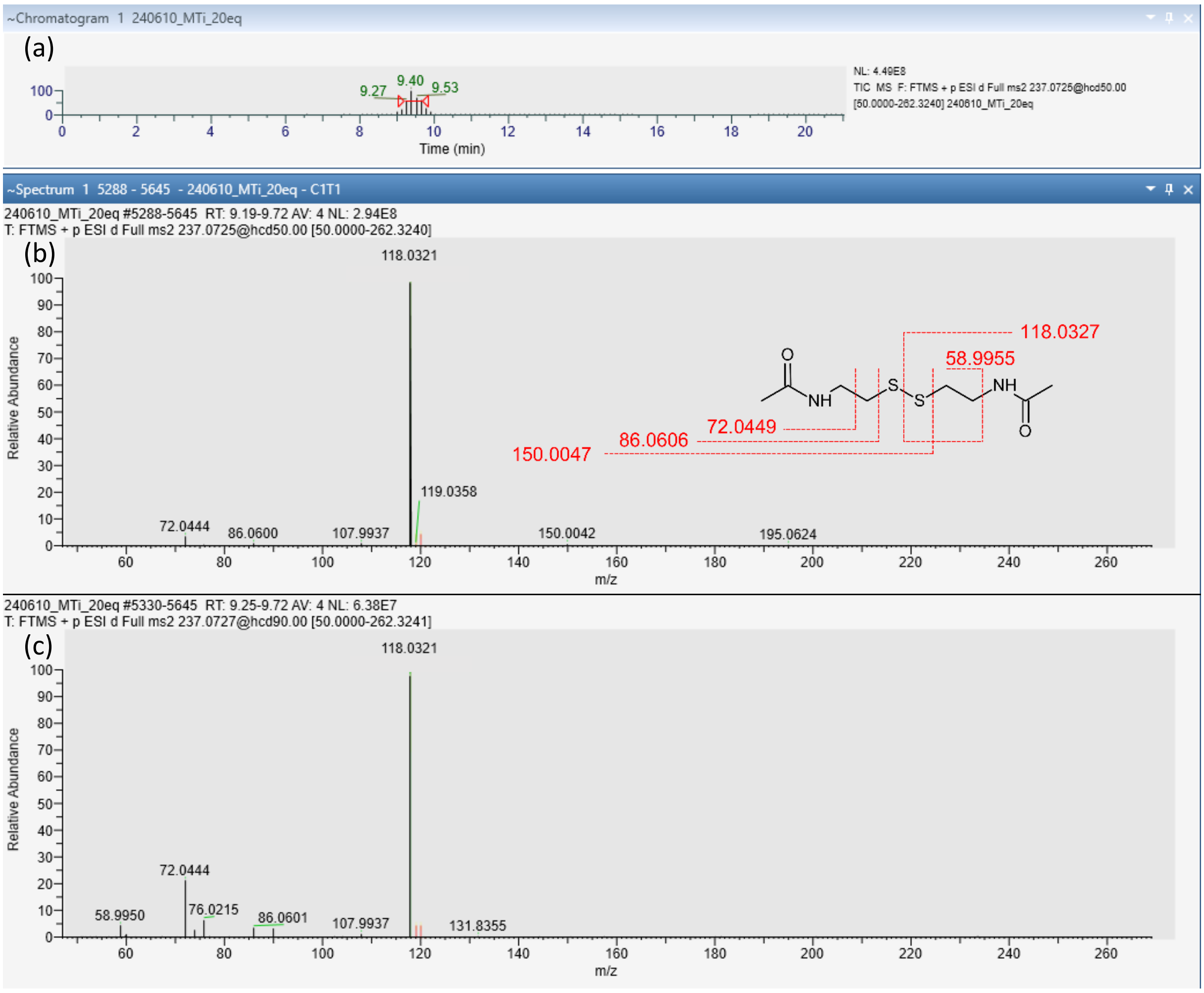

Figure S11.2 – (a) LC-HRMS/MS chromatogram (LC method E, Table S5.5) for an ozonated sample of 2-methylthiazoline in presence of the •OH scavenger *tert*-butanol, filtered by *m/z* = 237.0726 (**I1**+H⁺) and (b) and (c) MS^2 spectra of *m/z* = 237.0726 (**I1**+H⁺) at an HCD of 50% and 90%, respectively. The observed fragments can be assigned to the proposed structure of **I1** ((b), red fragmentation lines). The fragmentation is discussed in the text above. Experimental conditions: [2-methylthiazoline] = 2 mM; ozone dose = 0.1 mM; [*tert*-butanol] = 10 mM; [phosphate buffer (pH 7)] = 10 mM

S11.2 LC-HRMS/MS data of **I2**

The chemical structure of **I2** has been elucidated, using the following HRMS/MS data and NMR data, as discussed in Sections 3.2.4 (main manuscript) and S12.1. The isotopic pattern matches that of a S_2-species.

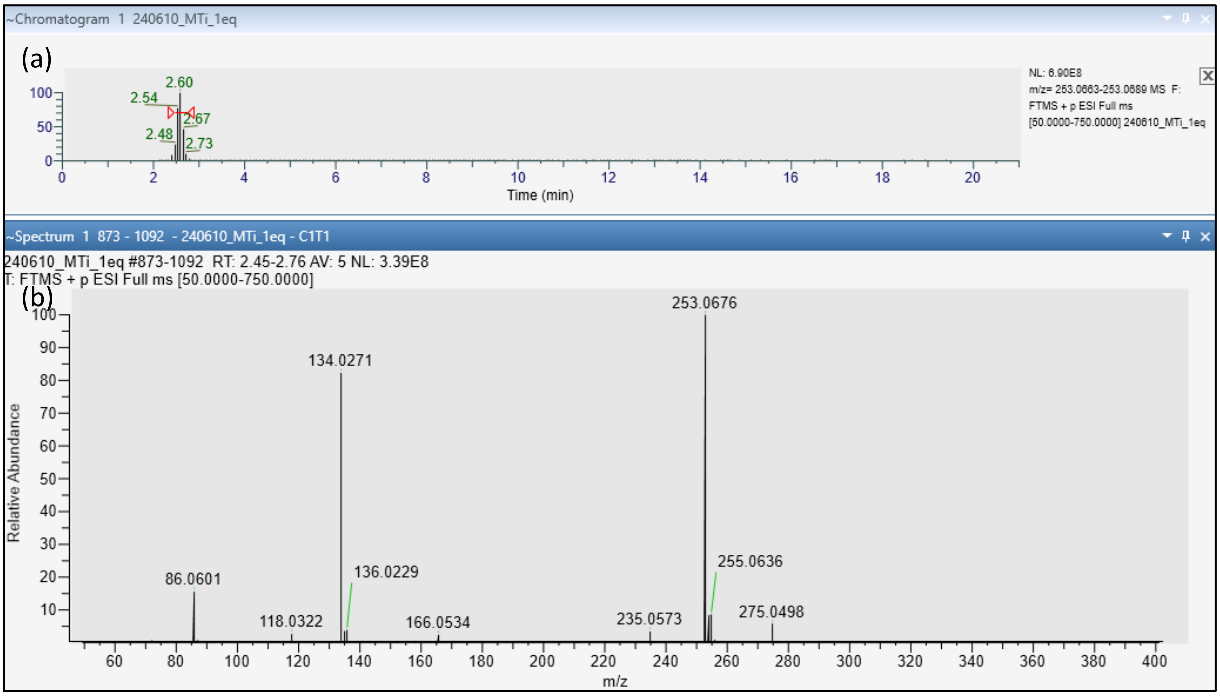

Figure S11.3 – (a) LC-HRMS/MS chromatogram (LC method E, Table S5.5) for an ozonated sample of 2-methylthiazoline in presence of the •OH scavenger *tert*-butanol, filtered by *m/z* = 253.0676 (**I2**+H$^+$) and (b) MS^1 spectrum of the peak at the retention time of 2.6 min. Experimental conditions: [2-methylthiazoline] = 0.1 mM; ozone dose = 0.1 mM; [*tert*-butanol] = 10 mM; [phosphate buffer (pH 7)] = 10 mM

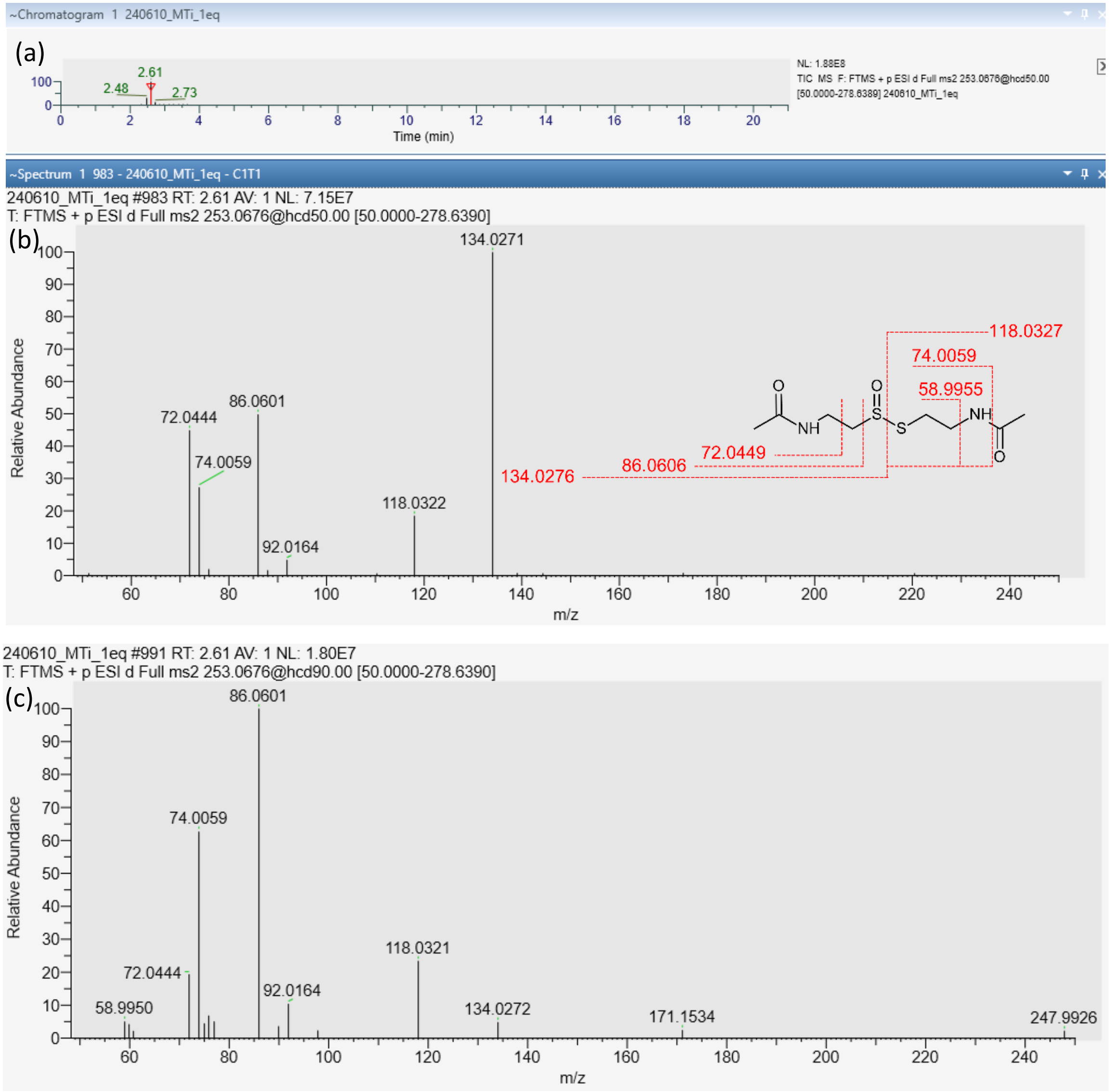

Figure S11.4 – (a) LC-HRMS/MS chromatogram (LC method E, Table S5.5) for an ozonated sample of 2-methylthiazoline in presence of the $^{\bullet}$OH scavenger *tert*-butanol, filtered by *m/z* = 253.0676 (**I2**+H$^+$) and (b) and (c) MS^2 spectra of *m/z* = 253.0676 (**I2**+H$^+$) at an HCD of 50% and 90%, respectively. The observed fragments can be assigned to the proposed structure of **I2** ((b), red fragmentation lines). The fragmentation is discussed in Section 3.2.4 (main manuscript). Experimental conditions: [2-methylthiazoline] = 0.1 mM; ozone dose = 0.1 mM; [*tert*-butanol] = 10 mM; [phosphate buffer (pH 7)] = 10 mM

S11.3 LC-HRMS/MS data of **I3**

The structure of **I3** has not been elucidated. The MS[1] spectrum (Figure S11.5) suggests the molecular formula $C_8H_{14}N_2O_3S_2$ for **I3**. The isotopic pattern matches that of a S_2-species. The *m/z* of several fragments in the MS[2] spectra have been observed for compound **I1** and **I2**. Fragment 150.0043 could suggest a non-oxidized disulfide-2-acetimido species (as seen for **I1**, Figure S11.2), which is also supported by the fragments at 118.0321, 107.9937, 90.0009, 86.0600, and 72.0444, all observed as well for disulfide **I1**. The additional fragments that have not been observed for **I1** or **I2** are 132.0114 ($C_4H_5NO_2S+H^+$), 120.0478 ($C_4H_9NOS+H^+$), 103.0212 ($C_4H_6OS+H^+$) and 78.0372 ($C_2H_7NS+H^+$). At higher collision energy, more fragments are observed. Attempts to propose a structure did not lead to satisfactory results.

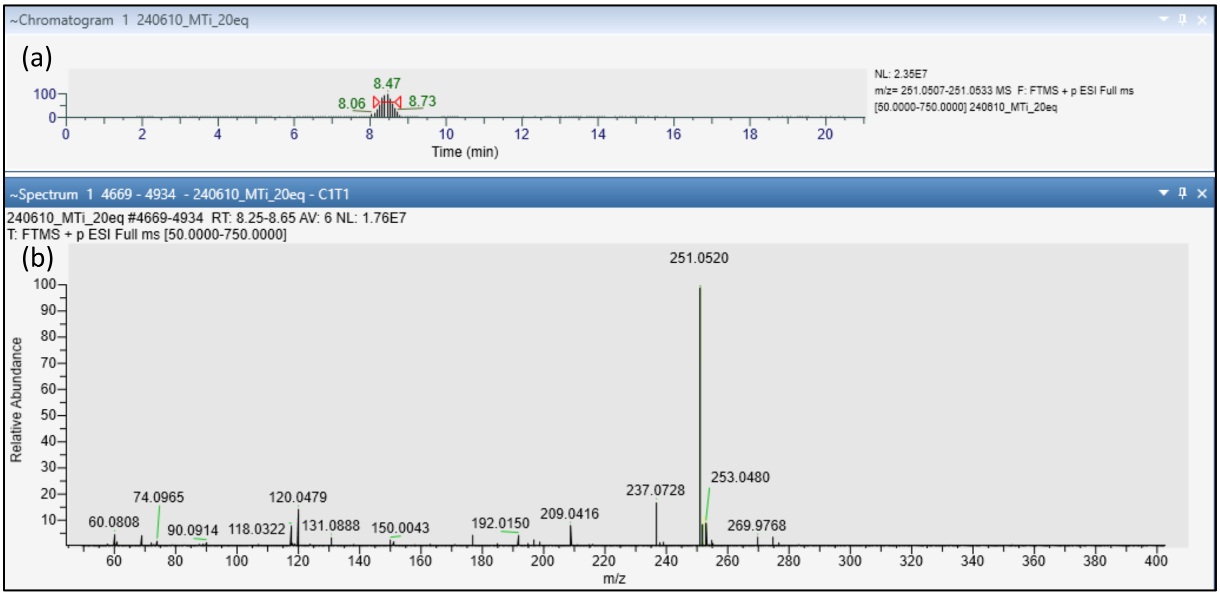

Figure S11.5 – (a) LC-HRMS/MS chromatogram (LC method E, Table S5.5) for an ozonated sample of 2-methylthiazoline, filtered by *m/z* = 251.0520 (**I3**+H$^+$) and (b) MS[1] spectrum of the peak at the retention time of 8.5 min. Experimental conditions: [2-methylthiazoline] = 2 mM; ozone dose = 0.1 mM; [*tert*-butanol] = 10 mM; [phosphate buffer (pH 7)] = 10 mM

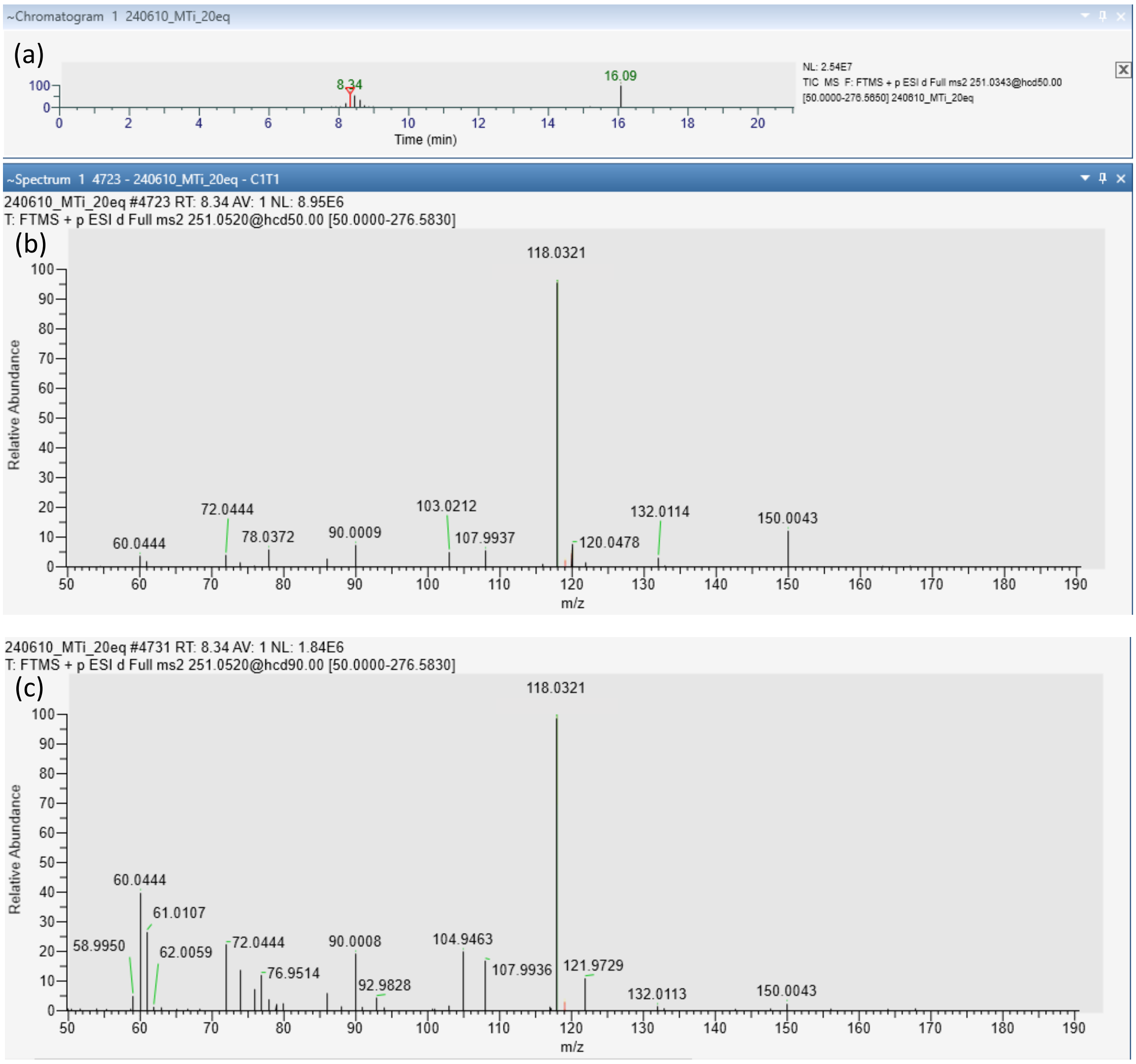

Figure S11.6 – (a) LC-HRMS/MS chromatogram (LC method E, Table S5.5) for an ozonated sample of 2-methylthiazoline, filtered by *m/z* = 251.0520 (**I3**+H$^+$) and (b) and (c) MS^2 spectra of *m/z* = 251.0520 (**I3**+H$^+$)at an HCD of 50% and 90%, respectively. The observed fragments have not been assigned to a structure. The fragmentation is discussed in Section 3.2.4 (main manuscript) and the text above. Experimental conditions: [2-methylthiazoline] = 2 mM; ozone dose = 0.1 mM; [*tert*-butanol] = 10 mM; [phosphate buffer (pH 7)] = 10 mM.

S11.4 LC-HRMS/MS data of **P1** (*N*-acetyltaurine) and co-eluting substances

P1 has been conclusively identified as *N*-acetyltaurine, using the HRMS/MS-data below and the NMR data, as described in Section S12.2. The isotopic pattern matches that of a S_1-species. The MS^2 spectrum (negative mode, Figure S11.9d) shows the same fragmentation pattern, as reported in the literature (Figure 11.9e). The role of the sulfinic acid **P1**–O (Figure S11.7, S11.8, S11.10 and S11.11) is discussed in Section 3.2.4 (main manuscript).

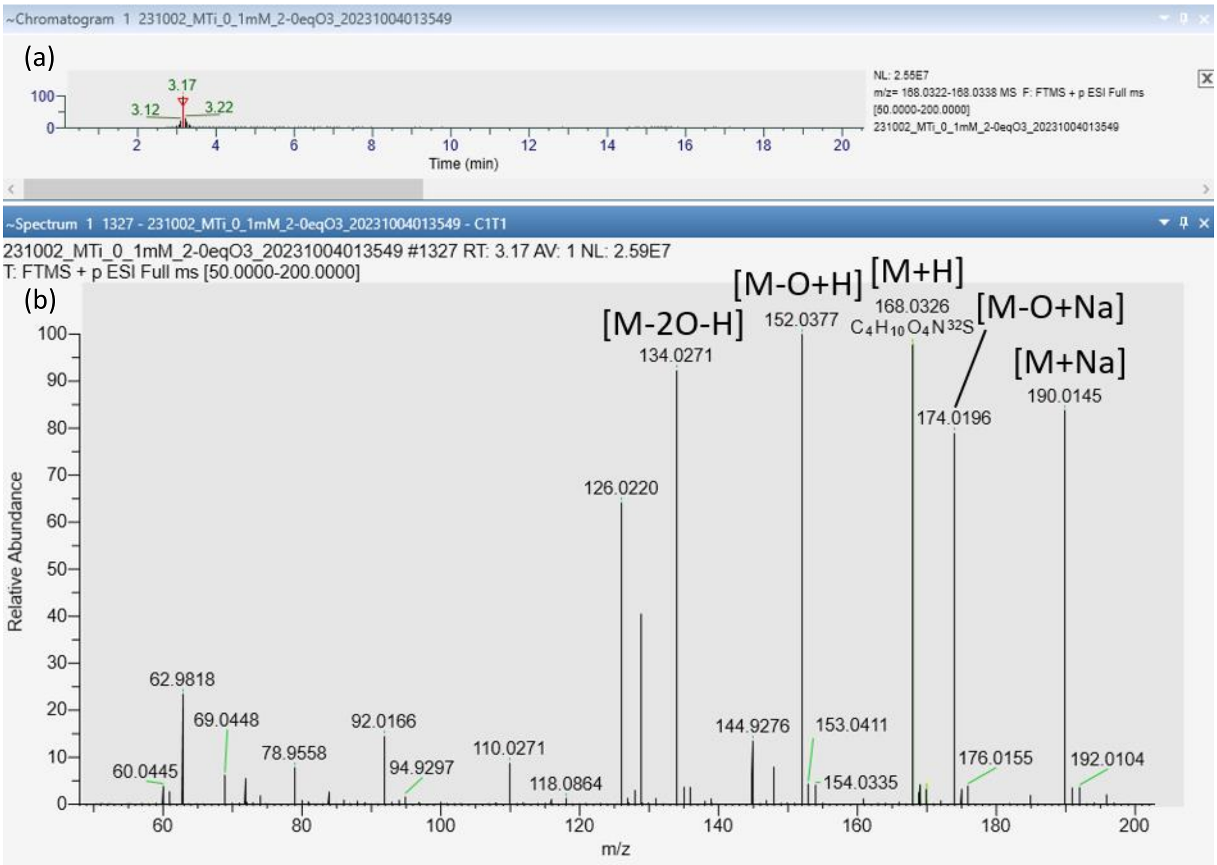

Figure S11.7 – (a) LC-HRMS/MS chromatogram (LC method F, Table S5.6) for an ozonated sample of 2-methylthiazoline, filtered by *m/z* = 168.0326 (**P1**+H$^+$) and (b) MS^1 spectrum of the peak at the retention time of 3.2 min. Experimental conditions: [2-methylthiazoline] = 0.1 mM; ozone dose = 0.2 mM; [*tert*-butanol] = 10 mM; [phosphate buffer (pH 7)] = 10 mM.

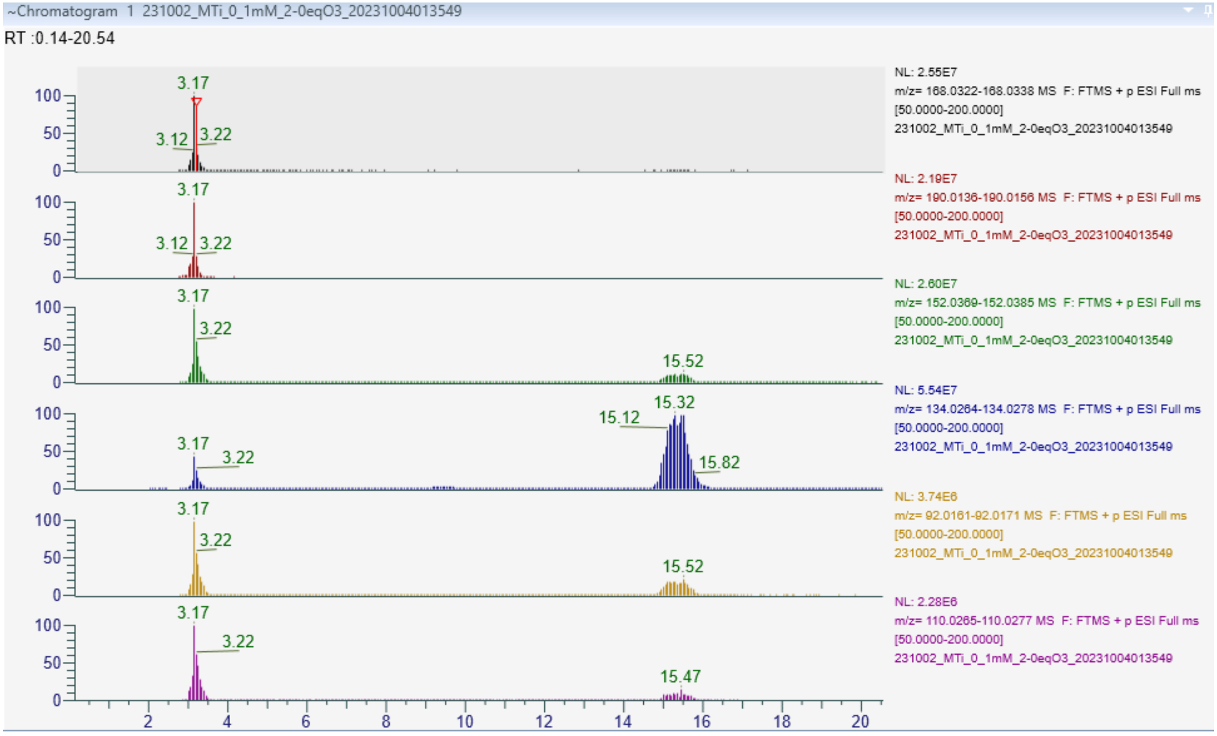

Figure S11.8 – Overlap of adducts and (in-source) fragmentations of **P1**. LC-HRMS/MS chromatogram (LC method F, Table S5.6) for an ozonated sample of 2-methylthiazoline, filtered by (from top to bottom) *m/z* = 168.0326 (**P1**+H$^+$); 190.0146 (**P1**+Na$^+$); 152.0377 (**P1**–O+H$^+$); 134.0271 (**P1**–2O+H$^+$); 92.0166; 110.0271. Experimental conditions: [2-methylthiazoline] = 0.1 mM; ozone dose = 0.2 mM; [*tert*-butanol] = 10 mM; [phosphate buffer (pH 7)] = 10 mM.

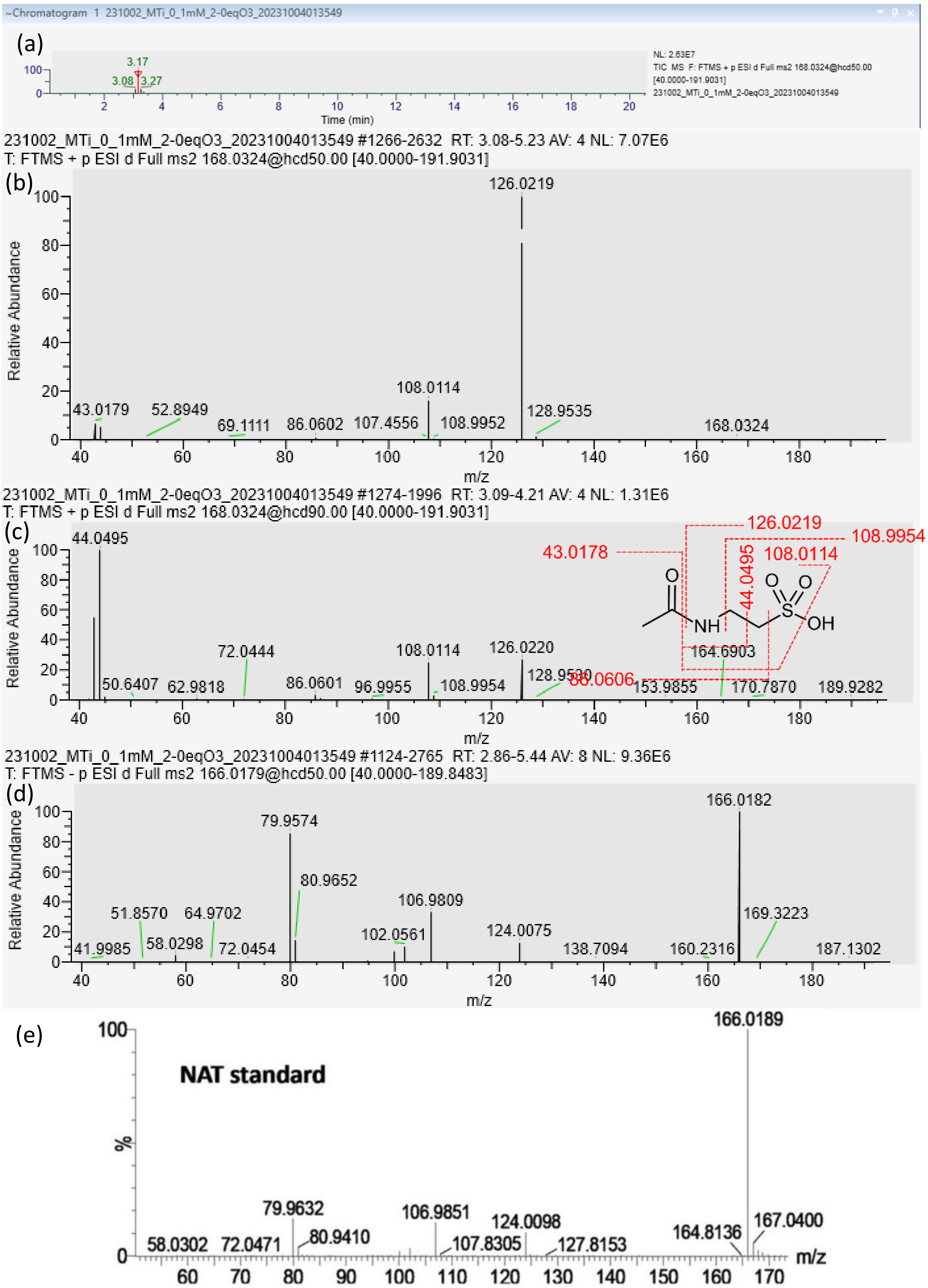

Figure S11.9 – (a) LC-HRMS/MS chromatogram (LC method F, Table S5.6) for an ozonated sample of 2-methylthiazoline, filtered by *m/z* = 168.0326 (**P1**+H$^+$) and (b) and (c) MS^2 spectra of *m/z* = 168.0326 (**P1**+H$^+$) at an HCD of 50% and 90%, respectively. The observed fragments can be assigned to the proposed structure of **P1** ((b), red fragmentation lines). (d) MS^2 spectra of *m/z* = 166.0182 (**P1**–H$^+$) at an HCD of 50%, for comparison with the literature. Experimental conditions: [2-methylthiazoline] = 0.1 mM; ozone dose = 0.2 mM; [*tert*-butanol] = 10 mM; [phosphate buffer (pH 7)] = 10 mM. (e) MS^2 spectra of *N*-acetyltaurine (NAT, *m/z* = 166.0182 (**P1**–H$^+$)), measured by Shi et al. (Reprinted with permission (no changes) from Ref. 30; Copyright 2012 ASBMB. Currently published by Elsevier Inc; originally published by American Society for Biochemistry and Molecular Biology, https://creativecommons.org/licenses/by/3.0/).[30]

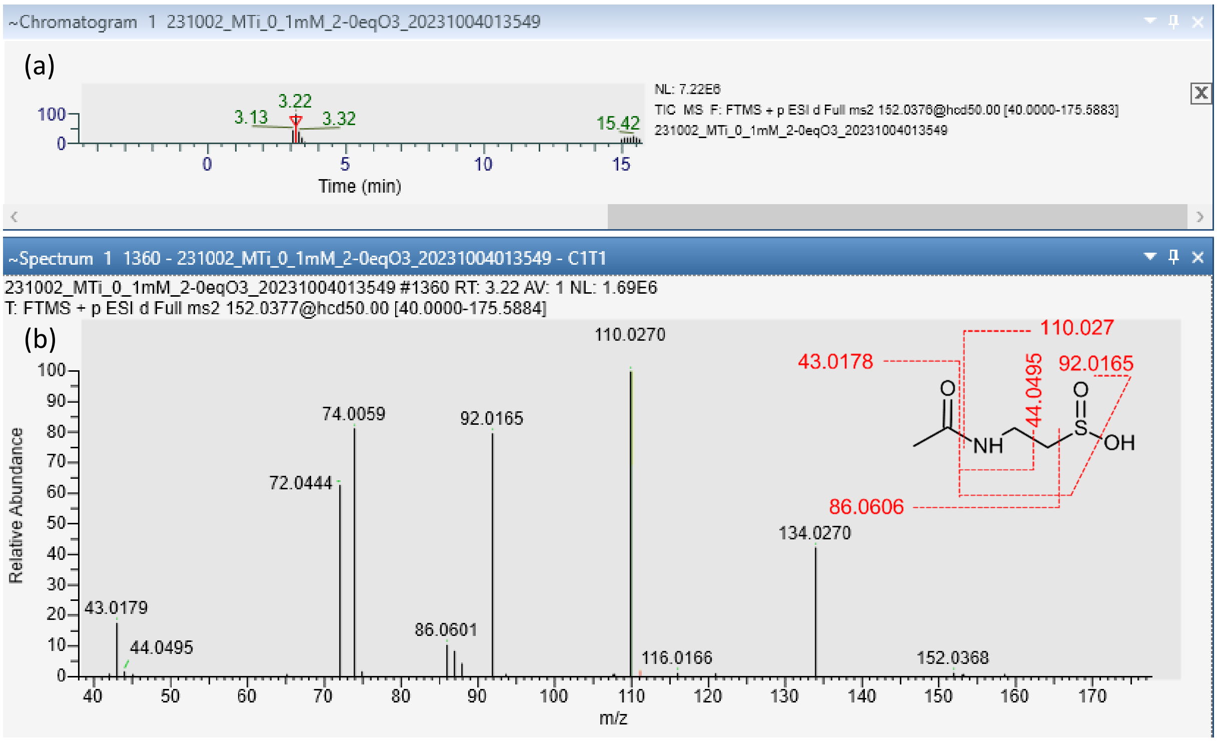

Figure S11.10 – (a) LC-HRMS/MS chromatogram (LC method F, Table S5.6) for an ozonated sample of 2-methylthiazoline, filtered by *m/z* = 152.0368 (**P1**–O+H$^+$) and (b) MS^2 spectra of *m/z* = 152.0368 (**P1**–O+H$^+$), corresponding to the sulfinic acid at an HCD of 50%. The observed fragments can be assigned to the proposed structure of **P1**–O ((b), red fragmentation lines). Experimental conditions: [2-methylthiazoline] = 0.1 mM; ozone dose = 0.2 mM; [*tert*-butanol] = 10 mM; [phosphate buffer (pH 7)] = 10 mM.

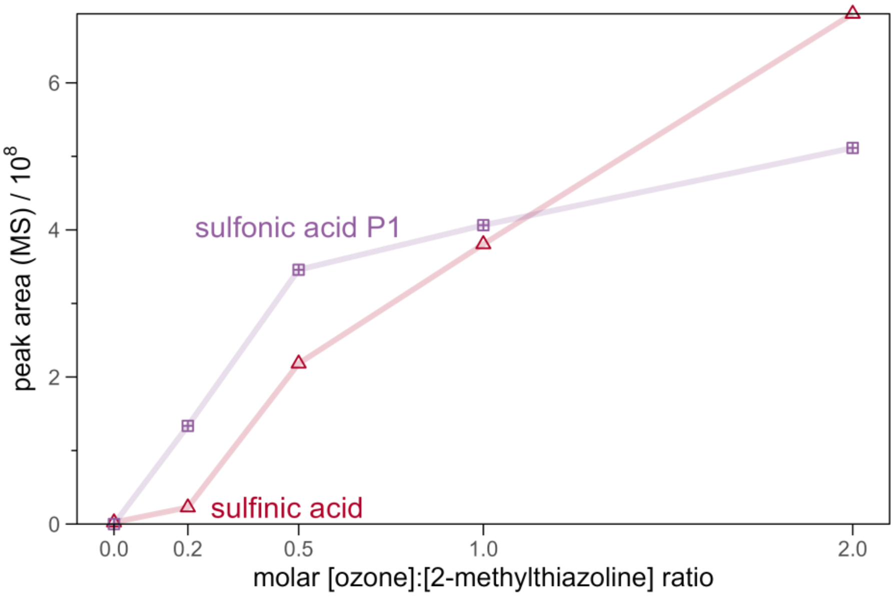

Figure S11.11 – Formation trend of sulfinic acid **P1**–O and sulfonic acid **P1** (*N*-acetyltaurine), measured by LC-HRMS/MS. Experimental conditions: [2-methylthiazoline] = 0.1 mM; ozone dose = 0–0.2 mM; [*tert*-butanol] = 10 mM; [phosphate buffer (pH 7)] = 10 mM.

S12 NMR-data for the identification of compounds **I2** and **P1**

In the samples from the ozonolysis of 2-methylthiazoline described here, the presence of several 2-acetamidoethane species in mixtures were observed with the aid of NMR data (data not shown), which were not easily identified at that time. Initially, it was suspected that the 2-acetamidoethane residue must be coupled to differently oxidized sulfur residues. In order to obtain more clearly identifiable analytical data (NMR, MS), a mixture of the ozonolysis products was purified using LC with sample collection as described Section S4.3. Below, the assignment of the observed NMR data of the two samples is shown, corresponding to the products **P1** and **I2**, as confirmed by LC-UV and LC-MS to the chemical species *N*-acetyltaurine (**P1**) and S-(2-acetamidoethyl) 2-acetamidoethane-1-sulfinothioate (**I2**), respectively.

S12.1 NMR data of **I2** (S-(2-acetamidoethyl) 2-acetamidoethane-1-sulfinothioate)

In the 1D and 2D NMR spectra (Figures S12.1 and S12.2) recorded for **I2**, the resonances assignable to two 2-acetamidoethane species were identified, with the ^{1}H, ^{13}C and ^{15}N chemical shifts of the acetimido chemical species being very close to each other. The two *N*-ethane residues differ mainly in the ^{13}C NMR chemical shifts of 55.2 and 40.0 ppm of positions 1 and 6, respectively (see chemical structure in Scheme S12.1). A closer look at the 1D ^{1}H and ^{13}C NMR spectra (Figure S12.1) reveals that the relative signal intensities of the two described 2-acetamidoethane species are approximately the same and that these residues could therefore both belong to the same but larger molecule. From the MS data (exact mass M+H^{+} = 253.0677, Figure S11.4) the molecular formula $C_8H_{16}N_2O_3S_2$ can be derived, whereby two S and one O remain after subtracting two acetamidoethane residues. Based on the chemical structure (Scheme S12.1), one part of the molecule with the sulfoxide moiety due to ^{13}C NMR chemical shift reasons belongs to the methylene carbon at 55.2 ppm, and the other part must belong to the -S-CH$_2$- group at δ^{13}C = 40.0 ppm.

^{1}H NMR (DMSO-d$_6$, 400.2 MHz): δ 8.15 (*t(br)*, *J* = 4.9 Hz, 1H, H-3); 8.07 (*t(br)*, *J* = 5.0 Hz, 1H, H-8); 3.44 (*m*, 2H, H-2); 3.33 (*m*, 2H, H-7); 3.28 (*m*, 2H, H-2); 3.14 (*m*, 2H, H-6); 1.80 (*s*, 3H, H-5); 1.79 (*s*, 3H, H-10). ^{13}C NMR (DMSO-d$_6$, 100.6 MHz): δ 169.6 (*s*, C-4); 169.4 (*s*, C-9); 55.2 (*t*, C-1); 40.0 (*t*, C-7); 33.2 (*t*, C-2); 31.8 (*t*, C-6); 22.5 (*q*, C-5); 22.5 (*q*, C-10). ^{15}N NMR (DMSO-d$_6$, 40.6 MHz): δ 116.9 (N-9); 114.4 (N-3).

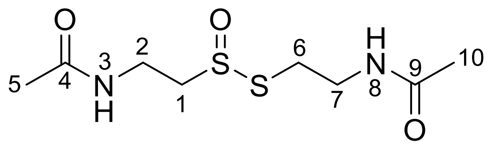

Scheme S12.1 – Chemical structure of **I2** with numbering of positions.

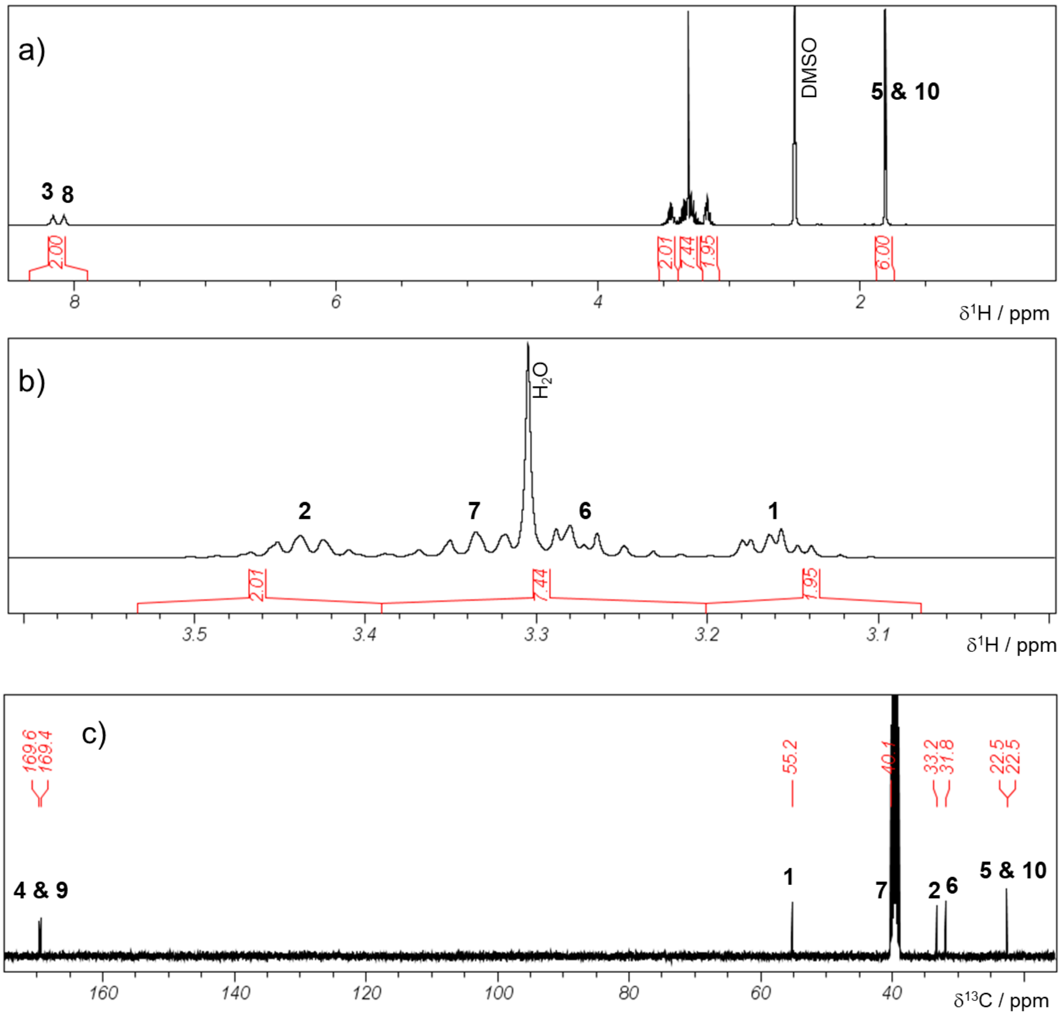

Figure S12.1 – ^{1}H and ^{13}C NMR spectra of **I2** with assignment of resonances to the chemical structure (Scheme S12.2). (a) ^{1}H NMR full spectral region, (b) enlarged chemical shift region and (c) ^{13}C NMR spectrum.

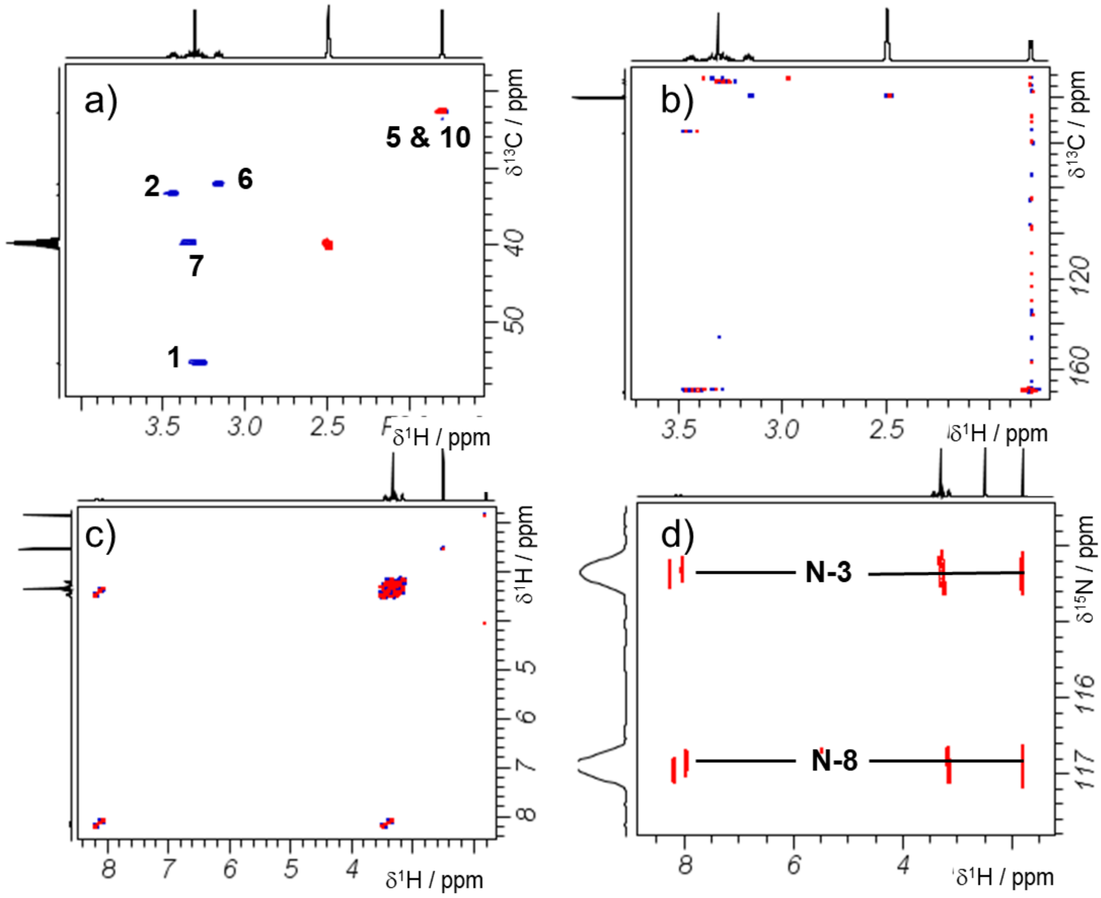

Figure S12.2 – 2D NMR spectra of **I2** with assignment of resonances to chemical structure (Scheme S12.1). Regions of interest of the (a) ^{1}H-^{13}C HSQC (b) ^{1}H-^{13}C HMBC, (c) ^{1}H-^{1}H DQF-COSY and (d) ^{1}H-^{15}N HMBC NMR spectra.

S12.2 NMR data of **P1** (*N*-acetyltaurine)

In the 1D and 2D ^{1}H, ^{13}C and ^{15}N NMR spectra (Figures S12.3 to S12.6) recorded for **P1**, the resonances assignable to one 2-acetamidoethane species were identified and for a solution of 1 mg of the reference compound "*N*-acetyltaurine" dissolved in 600 μL of DMSO all observed resonances / cross signals appeared at merely identical positions.

Therefore, the main compound identified in isolated **P1** unambiguously must be *N*-acetyltaurine (Scheme 12.2).

^{1}H NMR (DMSO-d$_6$, 400.2 MHz): δ 7.75 (*s(br)*, 1H, H-3); 3.26 (*m*, 2H, H-2); 2.54 (*t*, *J* = 7.4 Hz, 2H, H-1); 1.76 (*s*, 3H, H-5). ^{13}C NMR (DMSO-d$_6$, 100.6 MHz): δ 168.8 (*s*, C-4); 50.6 (*t*, C-1); 35.5 (*t*, C-2); 22.7 (*q*, C-5). ^{15}N NMR (DMSO-d$_6$, 40.6 MHz): δ 117.3 (N-3).

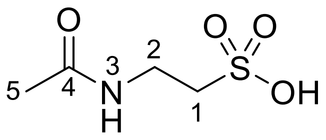

Scheme S12.2 – Chemical structure of **P1** and/or reference compound *N*-acetyltaurine with numbering of positions.

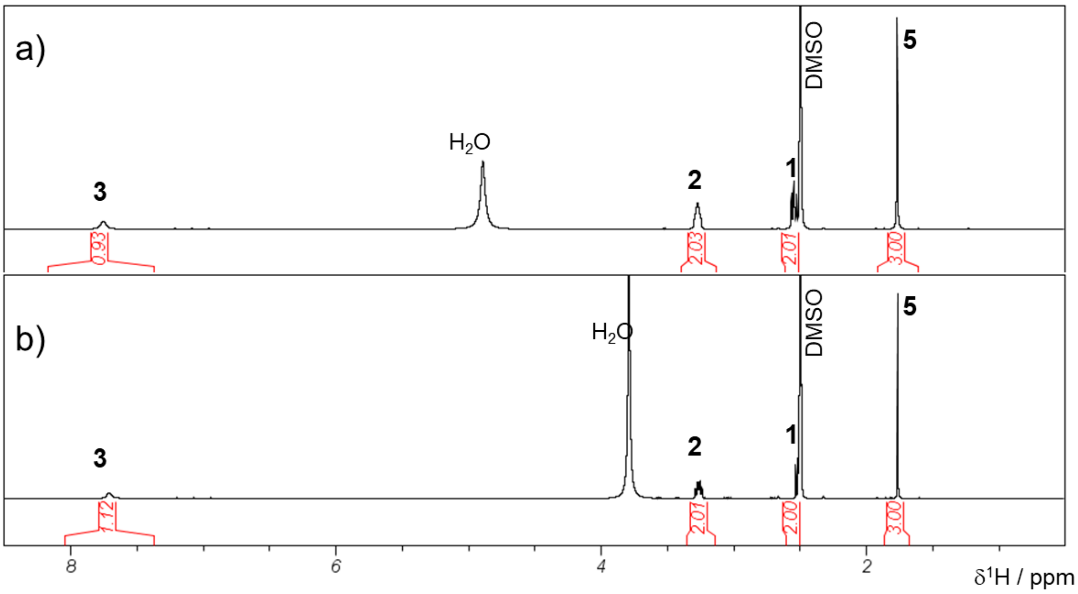

Figure S12.3 – Regions of interest of the ^{1}H NMR spectra of (a) **P1** and (b) reference compound *N*-acetyltaurine with assignment of resonances to chemical structure (Scheme S12.2).

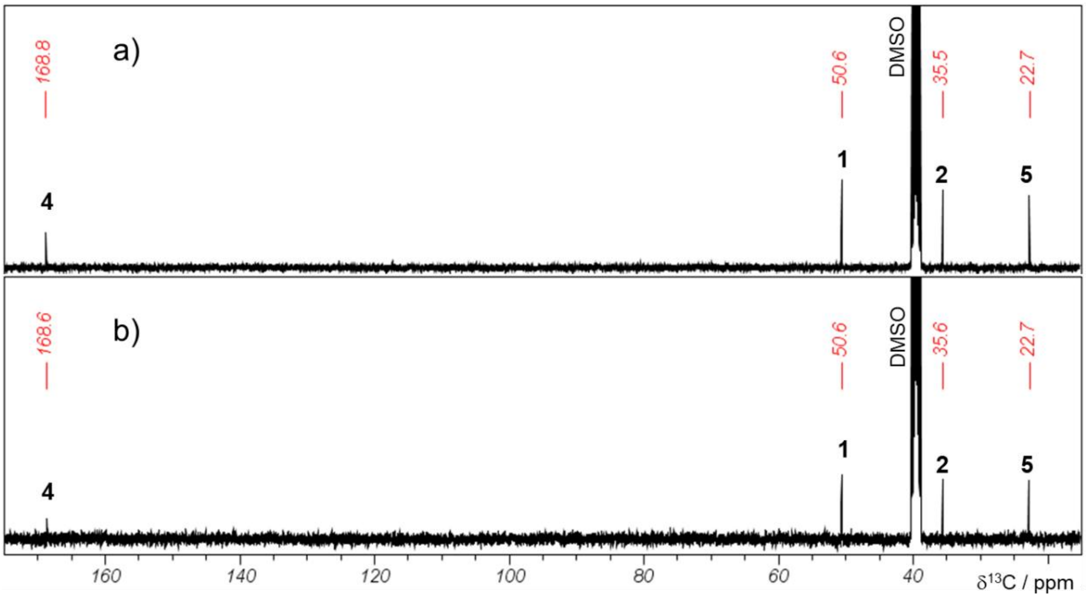

Figure 12.4 – Regions of interest of the ^{13}C NMR spectra of (a) **P1** and (b) reference compound *N*-acetyltaurine with assignment of resonances to chemical structure (Scheme S12.2).

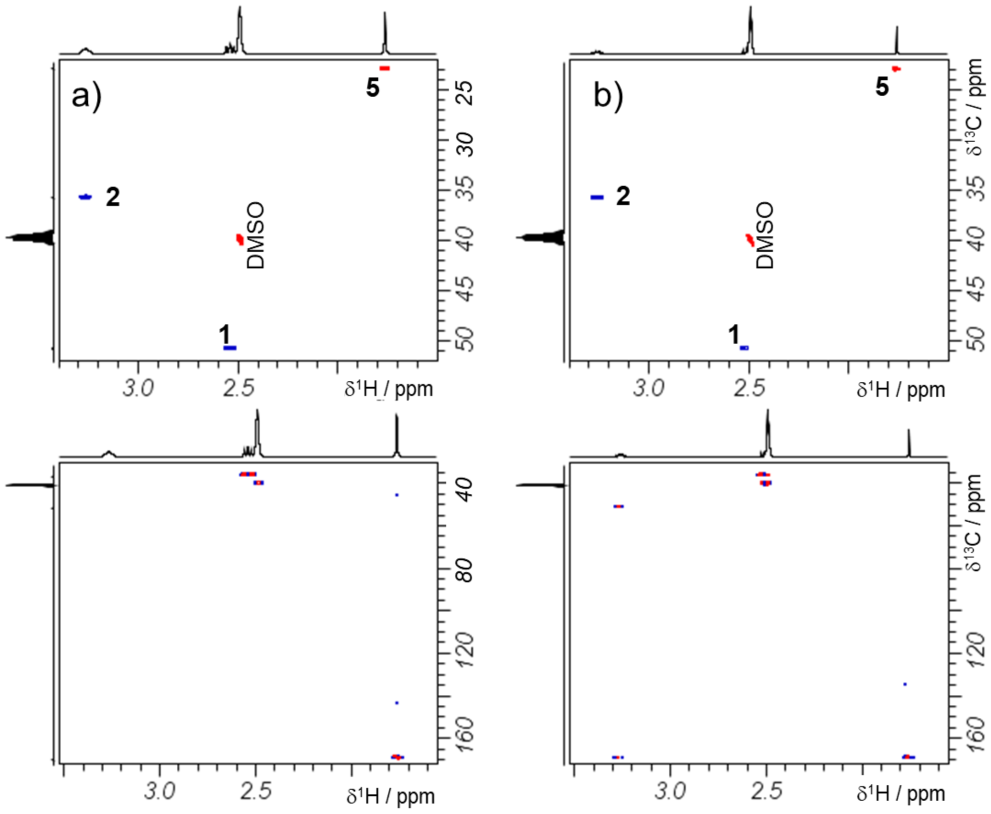

Figure S12.5 – Regions of interest of the ^{1}H-^{13}C HSQC (top) and HMBC (bottom) NMR spectra of (a) **P1** and (b) reference compound *N*-acetyltaurine with assignment of resonances to chemical structure (Scheme S12.2).

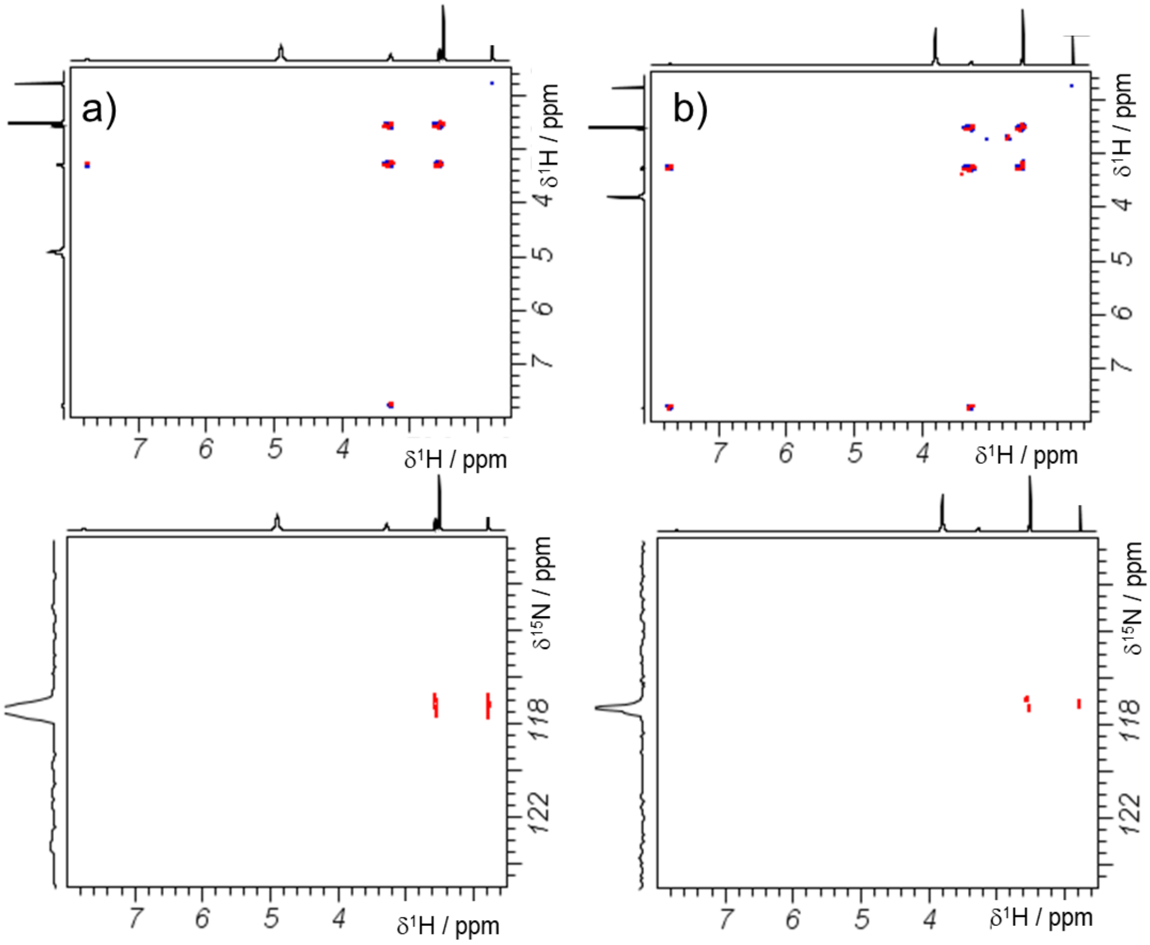

Figure S12.6 – Regions of interest of the ^{1}H-^{1}H DQF-COSY (top) and ^{1}H-^{15}N HMBC (bottom) NMR spectra of (a) **P1** and (b) reference compound *N*-acetyltaurine.

S13 References

(1) Tekle-Röttering, A.; Reisz, E.; Jewell, K. S.; Lutze, H. V.; Ternes, T. A.; Schmidt, W.; Schmidt, T. C. Ozonation of Pyridine and Other *N*-Heterocyclic Aromatic Compounds: Kinetics, Stoichiometry, Identification of Products and Elucidation of Pathways. *Water Res.* **2016**, *102*, 582–593. https://doi.org/10.1016/j.watres.2016.06.021.

(2) Tekle-Röttering, A.; Lim, S.; Reisz, E.; Lutze, H. V.; Abdighahroudi, M. S.; Willach, S.; Schmidt, W.; Tentscher, P. R.; Rentsch, D.; McArdell, C. S.; Schmidt, T. C.; von Gunten, U. Reactions of Pyrrole, Imidazole, and Pyrazole with Ozone: Kinetics and Mechanisms. *Environ. Sci. (Camb.)* **2020**, *6* (4), 976–992. https://doi.org/10.1039/C9EW01078E.

(3) Dodd, M. C.; Buffle, M.-O.; von Gunten, U. Oxidation of Antibacterial Molecules by Aqueous Ozone: Moiety-Specific Reaction Kinetics and Application to Ozone-Based Wastewater Treatment. *Environ. Sci. Technol.* **2006**, *40* (6), 1969–1977. https://doi.org/10.1021/es051369x.

(4) Valdés, H.; Zaror, C. A.; Jekel, M. Kinetic Study of Reactions between Ozone and Benzothiazole in Water. *Water Science and Technology* **2004**, *48* (11–12), 505–510. https://doi.org/10.2166/wst.2004.0900.

(5) Rougé, V.; von Gunten, U.; Janssen, E. M. L. Reactivity of Cyanobacteria Metabolites with Ozone: Multicompound Competition Kinetics. *Environ. Sci. Technol.* **2024**, *58* (26), 11802–11811. https://doi.org/10.1021/acs.est.4c02242.

(6) Jeon, D.; Kim, J.; Shin, J.; Hidayat, Z. R.; Na, S.; Lee, Y. Transformation of Ranitidine during Water Chlorination and Ozonation: Moiety-Specific Reaction Kinetics and Elimination Efficiency of NDMA Formation Potential. *J. Hazard. Mater.* **2016**, *318*, 802–809. https://doi.org/10.1016/j.jhazmat.2016.06.039.

(7) Zoumpouli, G. A.; Zhang, Z.; Wenk, J.; Prasse, C. Aqueous Ozonation of Furans: Kinetics and Transformation Mechanisms Leading to the Formation of α,β-Unsaturated Dicarbonyl Compounds. *Water Res.* **2021**, *203*, 117487. https://doi.org/10.1016/j.watres.2021.117487.

(8) Chen, W. R.; Wu, C.; Elovitz, M. S.; Linden, K. G.; (Mel) Suffet, I. H. Reactions of Thiocarbamate, Triazine and Urea Herbicides, RDX and Benzenes on EPA Contaminant Candidate List with Ozone and with Hydroxyl Radicals. *Water Res.* **2008**, *42* (1–2), 137–144. https://doi.org/10.1016/j.watres.2007.07.037.

(9) Hand, V. C.; Margerum, D. W. Kinetics and Mechanisms of the Decomposition of Dichloramine in Aqueous Solution. *Inorg. Chem.* **1983**, *22* (10), 1449–1456. https://doi.org/10.1021/ic00152a007.

(10) Bader, H.; Hoigné, J. Determination of Ozone in Water by the Indigo Method. *Water Res.* **1981**, *15* (4), 449–456. https://doi.org/10.1016/0043-1354(81)90054-3.

(11) von Sonntag, C.; von Gunten, U. *Chemistry of Ozone in Water and Wastewater Treatment: From Basic Principles to Applications*; IWA Publishing: London, 2012. https://doi.org/10.2166/9781780400839.

(12) Hoigné, J.; Bader, H. Characterization Of Water Quality Criteria for Ozonation Processes. Part II: Lifetime of Added Ozone. *Ozone Sci. Eng.* **1994**, *16* (2), 121–134. https://doi.org/10.1080/01919519408552417.

(13) Ra, J.; Huang, K.; Mohn, J.; Hofstetter, T. B.; Muck, E.; von Gunten, U. Characterization of Natural Organic Matter by Chlorination, Ozonation, and Stable Isotope Analysis of Nitrate. *Environ. Sci. Technol.* submitted.

(14) Criquet, J.; Rodriguez, E. M.; Allard, S.; Wellauer, S.; Salhi, E.; Joll, C. A.; von Gunten, U. Reaction of Bromine and Chlorine with Phenolic Compounds and Natural Organic Matter Extracts – Electrophilic Aromatic Substitution and Oxidation. *Water Res.* **2015**, *85*, 476–486. https://doi.org/10.1016/j.watres.2015.08.051.

(15) Rath, S. A.; von Gunten, U. Achieving Realistic Ozonation Conditions with Synthetic Water Matrices Comprising Low-Molecular-Weight Scavenger Compounds. *Water Res.* **2024**, *261*, 121917. https://doi.org/10.1016/J.WATRES.2024.121917.

(16) Wolf, C.; von Gunten, U.; Kohn, T. Kinetics of Inactivation of Waterborne Enteric Viruses by Ozone. *Environ. Sci. Technol.* **2018**, *52* (4), 2170–2177. https://doi.org/10.1021/acs.est.7b05111.

(17) Kim, M. S.; Lee, C. Ozonation of Microcystins: Kinetics and Toxicity Decrease. *Environ. Sci. Technol.* **2019**, *53* (11), 6427–6435. https://doi.org/10.1021/acs.est.8b06645.

(18) Ruttkies, C.; Schymanski, E. L.; Wolf, S.; Hollender, J.; Neumann, S. MetFrag Relaunched: Incorporating Strategies beyond in Silico Fragmentation. *J. Cheminform.* **2016**, *8* (1), 3. https://doi.org/10.1186/s13321-016-0115-9.

(19) Balcaen, L.; Woods, G.; Resano, M.; Vanhaecke, F. Accurate Determination of S in Organic Matrices Using Isotope Dilution ICP-MS/MS. *J. Anal. At. Spectrom.* **2013**, *28* (1), 33–39. https://doi.org/10.1039/C2JA30265A.

(20) Klencsár, B.; Sánchez, C.; Balcaen, L.; Todolí, J.; Lynen, F.; Vanhaecke, F. Comparative Evaluation of ICP Sample Introduction Systems to Be Used in the Metabolite Profiling of Chlorine-Containing Pharmaceuticals via HPLC-ICP-MS. *J. Pharm. Biomed. Anal.* **2018**, *153*, 135–144. https://doi.org/10.1016/j.jpba.2018.02.031.

(21) Tolu, J.; Bouchet, S.; Helfenstein, J.; Hausheer, O.; Chékifi, S.; Frossard, E.; Tamburini, F.; Chadwick, O. A.; Winkel, L. H. E. Understanding Soil Selenium Accumulation and Bioavailability through Size Resolved and Elemental Characterization of Soil Extracts. *Nat. Commun.* **2022**, *13* (1), 6974. https://doi.org/10.1038/s41467-022-34731-6.

(22) Templeton, D. M.; Ariese, F.; Cornelis, R.; Danielsson, L.-G.; Muntau, H.; van Leeuwen, H. P.; Lobinski, R. Guidelines for Terms Related to Chemical Speciation and Fractionation of Elements. Definitions, Structural Aspects, and Methodological Approaches (IUPAC Recommendations 2000). *Pure and Applied Chemistry* **2000**, *72* (8), 1453–1470. https://doi.org/10.1351/pac200072081453.

(23) Hoigné, J.; Bader, H. Rate Constants of Reactions of Ozone with Organic and Inorganic Compounds in Water-II. Dissociating Organic Compounds. *Water Res.* **1983**, *17* (2), 185–194. https://doi.org/10.1016/0043-1354(83)90099-4.

(24) Pryor, W. A.; Giamalva, D. H.; Church, D. F. Kinetics of Ozonation. 2. Amino Acids and Model Compounds in Water and Comparisons to Rates in Nonpolar Solvents. *J. Am. Chem. Soc.* **1984**, *106* (23), 7094–7100. https://doi.org/10.1021/ja00335a038.

(25) Yao, C. C. D.; Haag, W. R. Rate Constants for Direct Reactions of Ozone with Several Drinking Water Contaminants. *Water Res.* **1991**, *25* (7), 761–773. https://doi.org/10.1016/0043-1354(91)90155-J.

(26) Flyunt, R.; Makogon, O.; Schuchmann, M. N.; Asmus, K. D.; Von Sonntag, C. OH-Radical-Induced Oxidation of Methanesulfinic Acid. The Reactions of the Methanesulfonyl Radical in the Absence and Presence of Dioxygen. *Journal of the Chemical Society, Perkin Transactions 2* **2001**, No. 5, 787–792. https://doi.org/10.1039/B009631H.

(27) Wilkinson, F.; Helman, W. P.; Ross, A. B. Rate Constants for the Decay and Reactions of the Lowest Electronically Excited Singlet State of Molecular Oxygen in Solution. An Expanded and Revised Compilation. *J. Phys. Chem. Ref. Data* **1995**, *24* (2), 663–677. https://doi.org/10.1063/1.555965.

(28) Scully, F. E.; Hoigné, J. Rate Constants for Reactions of Singlet Oxygen with Phenols and Other Compounds in Water. *Chemosphere* **1987**, *16* (4), 681–694. https://doi.org/10.1016/0045-6535(87)90004-X.

(29) Ossola, R.; Jönsson, O. M.; Moor, K.; McNeill, K. Singlet Oxygen Quantum Yields in Environmental Waters. *Chem. Rev.* **2021**, *121* (7), 4100–4146. https://doi.org/10.1021/acs.chemrev.0c00781.

(30) Shi, X.; Yao, D.; Chen, C. Identification of *N*-Acetyltaurine as a Novel Metabolite of Ethanol through Metabolomics-Guided Biochemical Analysis. *Journal of Biological Chemistry* **2012**, *287* (9), 6336–6349. https://doi.org/10.1074/JBC.M111.312199.
